# Classical quantum chemical solution of the FeMo-cofactor model to chemical accuracy and its implications

**DOI:** 10.1126/sciadv.aeg7106

**Published:** 2026-10-21

**Authors:** Huanchen Zhai, Chenghan Li, Xing Zhang, Zhendong Li, Seunghoon Lee, Garnet Kin-Lic Chan

**Affiliations:** ^1^ Division of Chemistry and Chemical Engineering, California Institute of Technology, Pasadena, CA 91125, USA.; ^2^ Initiative for Computational Catalysis, Flatiron Institute, 160 Fifth Avenue, New York, NY 10010, USA.; ^3^ Marcus Center for Theoretical Chemistry, California Institute of Technology, Pasadena, CA 91125, USA.; ^4^ Key Laboratory of Theoretical and Computational Photochemistry, Ministry of Education, College of Chemistry, Beijing Normal University, Beijing 100875, China.; ^5^ Department of Chemistry, Seoul National University, Seoul 08826, Republic of Korea.

## Abstract

We use classical quantum chemistry methods to estimate the ground-state energy to chemical accuracy in a model of the FeMo cofactor of nitrogenase, which is widely studied as a target of quantum computing. Our result relies on the insight that the ground-state problem can be characterized as one of ranking many competing, but largely single-configuration, states. This allows a combination of systematic high-order coupled cluster and density matrix renormalization group calculations together with an extrapolation protocol to obtain an accurate energy. Within the model, we identify several spin isomer candidates for the ground state that are degenerate to chemical accuracy. Beyond this model, we characterize the impact of additional electronic excitations and the cluster and protein geometric fluctuations on the low-lying electronic landscape. We find that many features of the landscape are retained in more detailed representations of nitrogenase, which points to the complexity of spectroscopic interpretations of the electronic structure of the FeMo cofactor.

## INTRODUCTION

The main source of reduced nitrogen for living things comes from nitrogenase, which converts N_2_ to NH_3_ at the FeMo cofactor (FeMo-co) (*1*, *2*). Because of its role in supporting life, the uncertainty surrounding the catalytic cycle, and its compositional richness with eight transition metal ions, FeMo-co has fascinated scientists for decades. After much effort (*3*–*5*), the complete atomic structure was resolved in (*6*, *7*). However, its electronic structure, central to reactivity, remains under intense debate.

FeMo-co’s complexity, arising from many unpaired electrons, has led to suggestions that it lies beyond the reach of classical quantum chemical simulation. Consequently, there has been much interest in the potential of quantum algorithms to compute its electronic structure. Estimating the cost for a quantum computer to obtain the ground-state energy to chemical accuracy (∼1 kcal/mol) within one or more FeMo-co models (*8*, *9*) is a common benchmark of quantum algorithms in quantum chemistry, with numerous resource estimates in the literature (*10*–*13*).

Here, we address how to perform the same task using classical quantum chemistry methods. We use a 76-orbital/152-qubit resting state model (*9*), the subject of most quantum resource estimates. On the basis of insight into the multiple configuration nature of the states, we devise classical protocols based on systematic high-order coupled cluster (CC) and density matrix renormalization group (DMRG) calculations that yield rigorous or empirical upper bounds to the ground-state energy. Extrapolating these, we predict the ground-state energy with an estimated uncertainty on the order of chemical accuracy. Having performed this long-discussed computational task, we next consider implications beyond the model. We distill a simpler computational procedure, which we apply to reveal the electronic landscape in realistic representations of the cofactor. We thus illustrate a path to a precise computational understanding of FeMo-co electronic structure.

## RESULTS

### FeMo-co models and the structure of the states

#### Structure and active space model

The atomic structure of the FeMo-co is shown in Fig. 1A with a core structure of Fe_7_MoS_9_C. We consider only the *E*_0_ resting state, where spectroscopic evidence supports a cluster total charge of −1 (*14*) and a ground state with *S* = 3/2 (*15*).

**Fig. 1.**
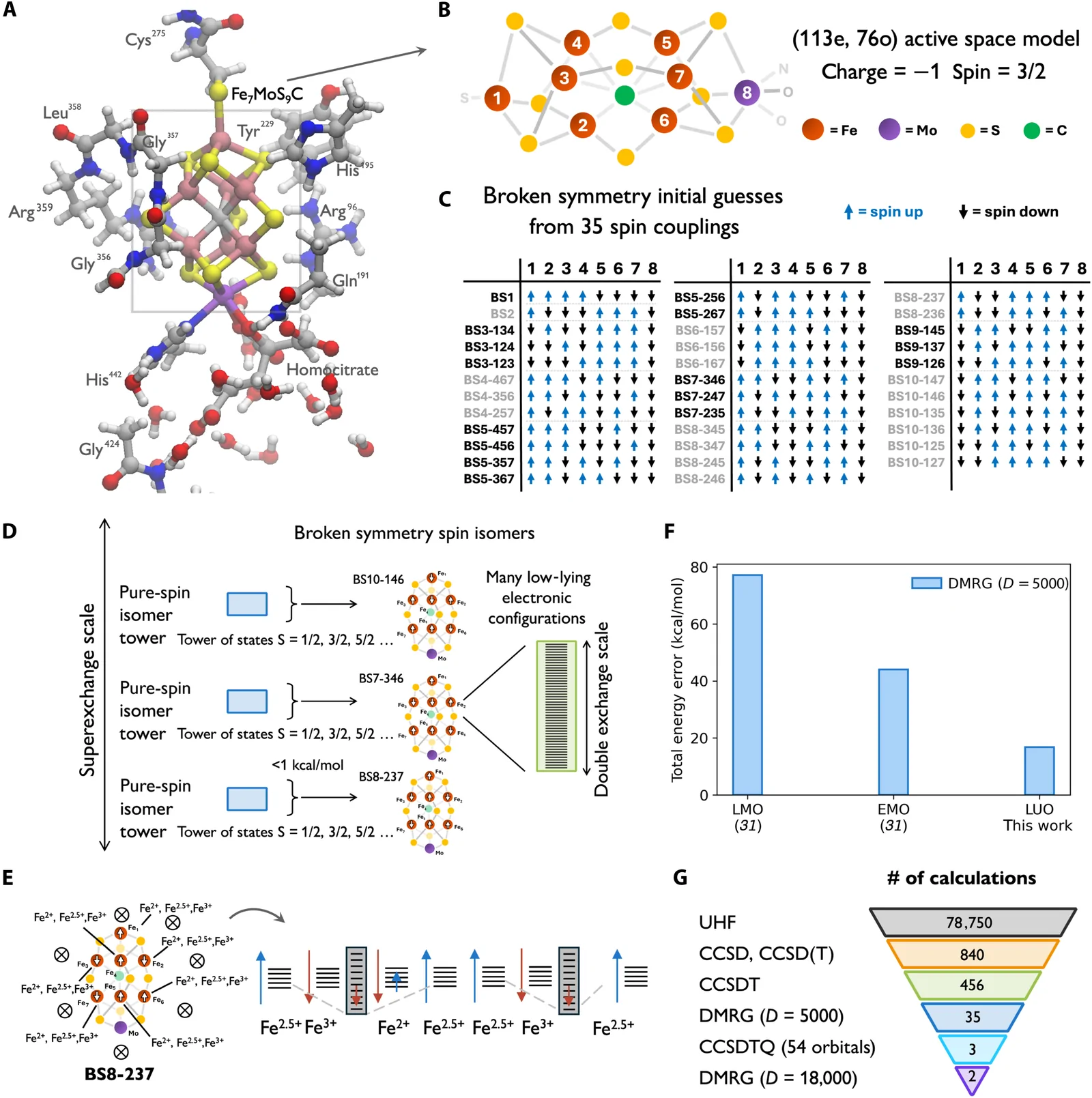
Structure, active space model, and states of FeMo-co. (**A**) Geometry of the FeMo-co core and surroundings, built from Protein Data Bank (PDB) 3U7Q (*6*, *9*). (**B**) Active space model with 113 electrons and 76 spatial orbitals from 22 atoms (*9*), with transition metals labeled by numbers (1 to 7 correspond to the crystallographic Fe nomenclature). (The model contains more atoms as shown in (A); this figure only shows atoms where the active space orbitals are located.) (**C**) Thirty-five broken-symmetry initial guesses representing different spin couplings. (**D**) Schematic showing the origin of broken-symmetry isomers from a tower of pure spin isomers; each broken-symmetry isomer further splits because of electron delocalization on the superexchange energy scale. The spin gap in each tower of states is expected to be <1 kcal/mol. (**E**) Schematic showing the interaction between potential electron delocalization and spin coupling in spin isomer BS8-237. (**F**) DMRG calculations with a bond dimension of 5000 using localized restricted spatial orbitals (LMO), entanglement-minimized restricted spatial orbitals (EMO) (*31*), and localized unrestricted orbitals (LUO; this work). The estimated ground state total energy of −22140.409107 hartrees is used as the energy reference. (**G**) Ranking and filtering of low-energy broken-symmetry configurations at different levels of theory.

The full structure we use is based on the crystal cutout in (*16*) and contains the cofactor, several protein residues, and nearby crystal water (Fig. 1A). We study two kinds of electronic models of this structure. The first uses the 113-electron, 76-orbital active space (Fig. 1B) of Li, Li, Dattani, Umrigar, and Chan (LLDUC) (*9*) with a continuum solvation treatment of the remaining protein environment. The active space spans the valence orbitals of the atoms in the FeMo-co core, i.e., Fe 3d, Mo 4d, S 3p, and C 2s, 2p orbitals, and some ligand 2p orbitals. We focus on this model because it has commonly served as the Hamiltonian for resource estimates for a quantum computer to compute the ground state to “chemical accuracy” [1.6 millihartrees (mH) or 1 kcal/mol] (*10*–*13*). Although this total energy is not physically measurable, it is a simple way to assess algorithmic cost. Our first goal will be to produce the best classical estimate of the total energy of this model.

The LLDUC model is not a quantitative model of FeMo-co electronic structure: The cofactor involves orbitals beyond the active space, a protein environment, and the atoms are moving. The second type of model we study extends the LLDUC model to account for these effects: to large active spaces (up to 404 orbitals); and to include protein fluctuations, modeled by molecular dynamics.

#### Classical spin and electronic configurations of FeMo-co

FeMo-co supports many low-energy states. One origin is the competing spin configurations. In the formal (II) and (III) oxidation states, the local spins of the Fe ions are *S* = 2 or *S* = 5/2, respectively, and Mo(III) has been suggested to be *S* = 1/2 with an unconventional configuration (*17*, *18*). Within a 1/*S* approximate expansion, the large Fe spins can be approximated as classical (*19*), and classical product states of spins (typically collinear) have been used to enumerate low-energy broken-symmetry states in density functional theory (DFT) calculations (*16*, *20*, *21*) and to seed more accurate wave function calculations (*22*–*24*). Assuming collinear spins (noncollinearity is considered in Supplementary Text) and a *S_z_* = 3/2 ground state, there are 35 spin isomers (Fig. 1C). These enumerate the arrangement of up and down spins, neglecting the distinction between Fe(II) and Fe(III). Further assuming threefold symmetry through the Fe1-Mo axis, these reduce to 10 isomer families labeled BS*n* (*n* = 1,…,10); the 35 spin isomers are labeled BS*n-ijk* where *ijk* labels the iron sites with the minority spin (see Supplementary Text for other nomenclature) (*16*). Each broken-symmetry spin isomer can be understood as arising from mixing a tower of pure-spin states for a given isomer (Fig. 1D).

The next source of complexity is the electronic degrees of freedom, due to the additional electron in Fe(II) versus Fe(III). Neglecting mixed valence configurations, each of the 35 spin isomers has 18 assignments of Fe(II) and Fe(III), and for each Fe(II), there are 5d orbitals for the extra electron. This generates 78,750 electronic configurations and more if one then includes mixed valence configurations (Fig. 1E). Navigating this complexity is the key challenge in the electronic structure of this system.

#### Prior computational and spectroscopic studies of FeMo-co

There have been many prior computational studies of FeMo-co (see review in Supplementary Text). Most (*21*, *25*) have used broken-symmetry DFT based on classical spin configurations. While the DFT total energies are not useful for comparison with the models here, the observed relative energy electronic landscape is relevant. Studies, e.g., of protonation energies, or the relative stability of spin isomers, find that these are exquisitely sensitive to the choice of density functional (*21*, *26*). Despite this sensitivity, recent BS-DFT studies have focused on the BS7-235 spin isomer as the ground state (*21*, *25*) because its optimized DFT geometry has been argued to give the best agreement with the x-ray structure (*25*).

Experimental studies of the FeMo-co (see review in Supplementary Text) have used electron paramagnetic resonance (*15*), Mössbauer spectroscopy (*27*), and x-ray diffraction analysis, (*6*, *7*), but there is only very indirect insight into the FeMo-co ground state. In most cases, it is difficult to resolve which part of the signal comes from which ion/unpaired electron, and one requires electronic structure calculations to deconvolve the signal (*16*). Some recent constraining evidence comes from site-selective ^57^Fe labeling of the FeMo-co cluster, which constrains the Fe1 spin to be aligned with the total spin of the ground state, and further argues, from a mixed valence assignment of Fe1, that a neighboring iron (Fe2, Fe3, or Fe4) should be spin aligned with Fe1 (*28*) [although this relies on a picture of resonance delocalization that is not always satisfied (*29*); see also Supplementary Text]. Another notable study, involving Se substitution coupled to x-ray absorption, suggested that Fe2 and Fe6 are more oxidized and antiferromagnetically coupled (*29*). Last, spatially resolved anomalous diffraction refinement has been used to infer iron oxidation states, although the connection of the signal to oxidation state is highly complex (*30*).

Beyond DFT calculations, FeS cluster electronic structure within active space models has received much attention (*8*, *22*–*24*, *31*–*37*). These studies, often using the DMRG (*38*), have highlighted the richness of the electronic landscape in the iron-cubane and P-cluster (*22*, *23*, *32*, *34*, *35*). However, in the FeMo-co LLDUC model [e.g., (*24*)], these calculations have been less accurate and have fallen far short of the accuracy of 1 kcal/mol of the quantum resource estimation task (*8*, *24*, *31*, *36*, *37*). For example, supplementary figure 8 in (*24*) estimates a residual error in the variational DMRG energy of ∼100 mH (63 kcal/mol), far too large to extrapolate the energy to chemical accuracy.

#### Classical electronic structure methods

The main methods we use here are the DMRG and CC theory. We combine them to obtain accurate estimates of the energy and observables. DMRG is a variational method controlled by a parameter (bond dimension) *D*, and the calculation can be made arbitrarily accurate by increasing *D* (*36*, *38*–*45*). CC theory provides a nonvariational estimate of the ground-state energy using excitations from a starting mean-field state (singles, doubles, and so on, giving CCSD, CCSDT, CCSDTQ, etc.); as the excitation level increases, the method becomes exact (*46*, *47*). The resource limiting the accuracy is different in the two methods: For DMRG, it is the entanglement, while for CC, it is the closeness to the initial mean-field configuration. Consensus of estimates from the two methods is thus a stringent consistency test.

In this work, we developed new or customized our existing DMRG and CC implementations. For example, for the CC calculations, we developed a new memory efficient, parallel implementation of arbitrary order CC (see Supplementary Text). However, the main methodological innovation is the special protocol we use in the calculations, rooted in the nature of the FeMo-co states (see details in Supplementary Text), which we now discuss.

### Qualitative nature of FeMo-co electronic states

We perform calculations that break the *S*^2^ spin symmetry of the system. This reduces the amount of entanglement in the modeled states and increases their mean-field character. Symmetry breaking is artifactual in a finite system, but in FeMo-co, the many coupled spins generate a small spin gap Δ. The relevant gap is that within the tower of pure-spin states of a fixed spin isomer (*48*) [expected to be <1 kcal/mol (*23*); for additional discussion see Supplementary Text], not the gap between spin isomers, and the pattern of spins in the broken-symmetry solution reflects the pattern of spin correlations in the tower from which it is derived; see Fig. 1D. If our desired energy precision >Δ, then the symmetry broken solution can represent the ground state to such precision (see Supplementary Text) and reflect the true correlations in the state. The caveat is that (in large systems) classical calculations initialized as one symmetry broken solution will not (due to ansatz restrictions) “tunnel” into another. Thus, we must start from different initial states, which then either converge to the (symmetry broken) ground state or a low-lying excited state.

Beyond spin excitations, we must also carefully consider the orbital/charge degrees of freedom (Fig. 1E). The spin and charge are strongly coupled: In a classical unrestricted mean field, orbitals for spin-up and spin-down delocalize differently because of the Pauli principle and double exchange (*49*). Consequently, different broken-symmetry states have very different spin-up and spin-down mean-field orbitals.

Prior DMRG calculations on FeS clusters have always used a single (“restricted”) orbital basis, where the spin-charge correlation must be entirely captured by the DMRG wave function. However, if we allow different orbitals for different spins (see Fig. 1F), then we find a large improvement in the DMRG energy at a given bond dimension, surpassing all previous choices of orbitals, as much of the spin-charge correlation is captured in the basis itself. Using this unusual “unrestricted orbital” DMRG (denoted as UDMRG) formed the basis for progress in this work. Correspondingly, we perform CC with spin-unrestricted HF reference (denoted as UCC).

Even after spin symmetry breaking, the many classical electronic configurations [e.g., 78,750 for localized Fe(II) and Fe(III) charges] pose a challenge. For example, if one starts from one local electronic configuration as a reference state for UCC and the actual ground state is another, then the reference is orthogonal to the ground state and UCC will fail (although UDMRG does not suffer from this problem; see Supplementary Text). Similarly, the possibility for delocalized versus localized electrons creates a multiconfigurational problem: Even in a simplified model where delocalized electrons only explore one valence orbital per Fe, the squared overlap of a reference mean-field state with only local Fe charges with the true ground state decreases by $∼\frac{1}{2}$ for every delocalized pair in the exact state. Thus, UCC calculations that start from the wrong initial electronic configuration and charge distribution cannot reliably describe the ground state. However, this multiconfigurational nature is arguably trivial because it can be removed by properly choosing the initial orbitals. The challenge is one of multiple competing configurations rather than an intrinsic multiconfigurational problem.

The ranking of electronic configurations and the balance between localized and delocalized configurations is intimately tied to the level of electron correlation. To obtain a reliable UCC description, we perform hundreds of UCCSD calculations to rank low-energy initial unrestricted Hartree-Fock (UHF) configurations and then further rank these downselected configurations using higher levels of UCC (up to quadruples, and even beyond, in the LLDUC model) and UDMRG (see Supplementary Text). This filtering funnel is illustrated in Fig. 1G. Recognizing that the multiconfigurational problem reduces to a ranking problem was the key to successfully using UCC to describe FeMo-co.

### The electronic landscape of the LLDUC model of the FeMo-co

We carried out UCC and UDMRG calculations using the filtering procedure (see Supplementary Text). In Fig. 2A, we show the energy landscape of the 35 spin isomers in the LLDUC model with UHF, UCC up to triples, and UDMRG with a moderate bond dimension *D* = 5000.

**Fig. 2.**
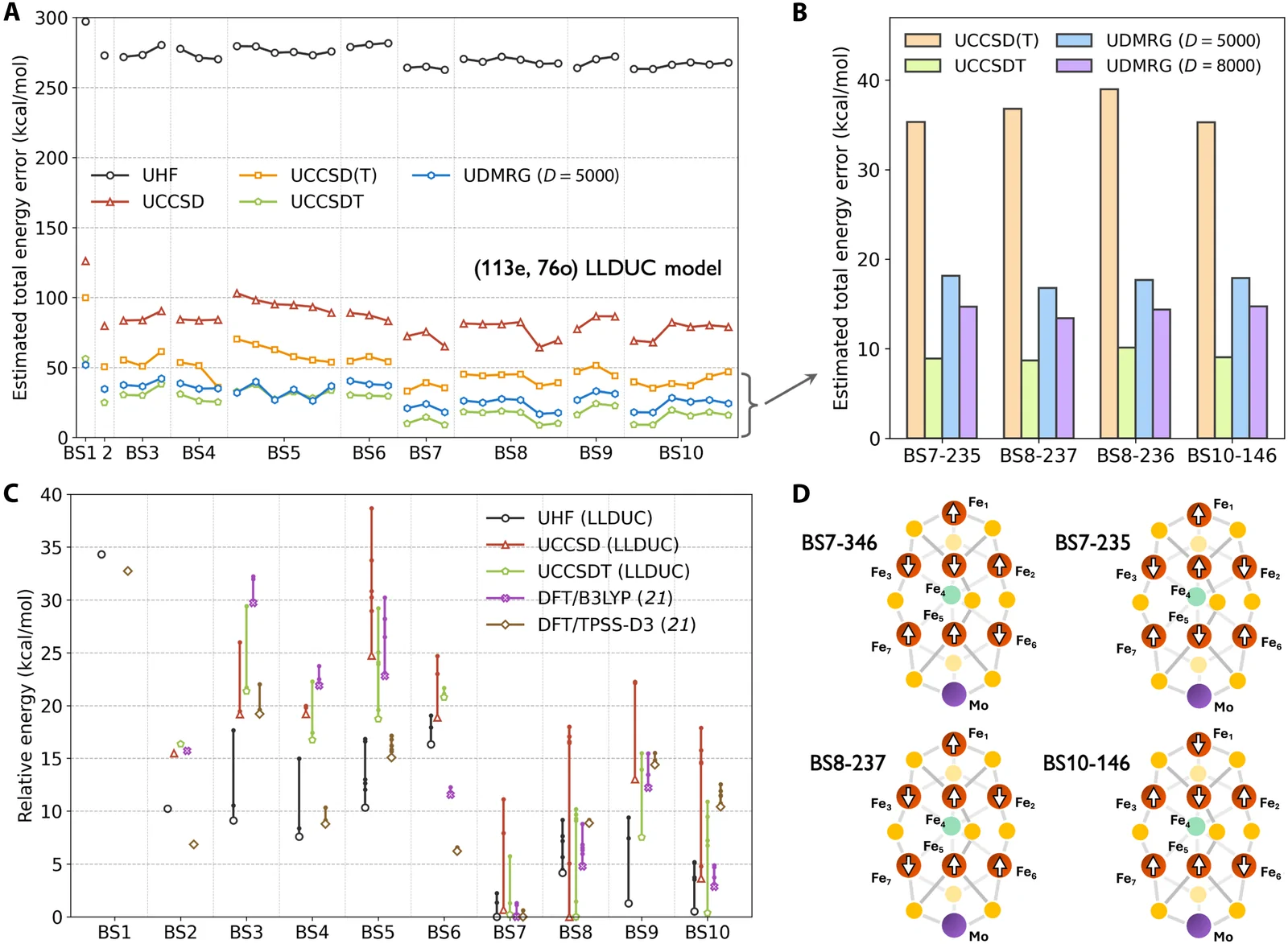
Electronic landscape of FeMo-co active space model. (**A** and **B**) Energies of different spin isomers computed using UHF, UCCSD, UCCSD(T), UCCSDT, and UDMRG with bond dimension *D* = 5000 and 8000 for the (113e, 76o) LLDUC model. The estimated ground state total energy of −22140.409107 hartrees is used as the energy reference. (**C**) Relative energies computed using UHF, UCCSD, and UCCSDT for the LLDUC model, compared with DFT results with B3LYP/def2-TZVPD and TPSS-D3/def2-TZVPD from (*21*). The minimum energy across all isomers for each theory is used as the energy reference. The lowest energy in each BS family is shown as a larger symbol. (**D**) Noodleman’s schematic for the BS states BS7-346, BS7-235, BS8-237, and BS10-146 showing the spin coupling (*21*, *66*).

We find convergent trends as the correlation level increases. At the highest levels of correlation in the figure, UCCSDT and UDMRG (*D* = 5000), the relative orderings between BS families and within them are consistent. (T) shows moderately strong reference dependence, reflecting the importance of self-consistent UCC amplitudes; see Supplementary Text. In addition, we note that the relative energy of the spin isomers within each broken-symmetry family can be different, mainly due to deviations from *C*_3_ symmetry in the geometry, for example, the energy of BS7-247 is much higher than that of the other two spin isomers in the BS7 family. Nonetheless, all the states correspond to (largely) single reference states (for various diagnostics, see Supplementary Text), confirming our qualitative insight into the states and the effectiveness of the filtering procedure.

It is assumed and to some extent observed in DFT calculations that within each BS*n* class, spin isomers are similar in energy (*16*, *21*). We find the variation between spin isomers for the same BS*n* class to be comparable to the variation between BS*n* classes. The quantitative variations are sensitive to the level of correlation (see Fig. 2C).

In the LLDUC model, while BS7 is the lowest energy class at the mean-field level (similar to as found in prior DFT studies), at the correlated wave function level, BS7, BS8, and BS10 are all low in energy, with BS8 becoming almost degenerate with (or lower in energy than) BS7 as the correlation level increases. BS7 maximizes the number of antiferromagnetic (AFM) couplings across Fe─S─Fe bonds, which is sometimes used to justify its low energy in DFT studies (*16*, *21*), but BS8 and BS10 do not (Fig. 2D). The qualitative origin of the relative energies thus requires balancing both exchange and double exchange, where double exchange modifies the charge distribution and delocalization across the cluster. The latter is a large energy scale, which we note has strong functional dependence in DFT (*21*). While there are several spin isomers of BS7, BS8, and BS10 that are very close in energy (see Supplementary Text), we next focus on BS7-235 and BS8-237 as candidates for the LLDUC ground state.

### Estimating the ground-state energy of the LLDUC model to chemical accuracy

To obtain a high accuracy estimate of the ground-state energy of FeMo-co in the LLDUC model, we push the level of the coupled cluster calculations up to quadruples (UCCSDTQ) with a limited estimate of pentuples and beyond and also extend the UDMRG calculations to a bond dimension of *D* = 18,000 for BS7-235 and BS8-237. We then separately extrapolate the UDMRG and UCC data to provide multiple estimates of the exact energy as shown in Fig. 3A (total energies are reported here in hartrees for easier reproducibility).

**Fig. 3.**
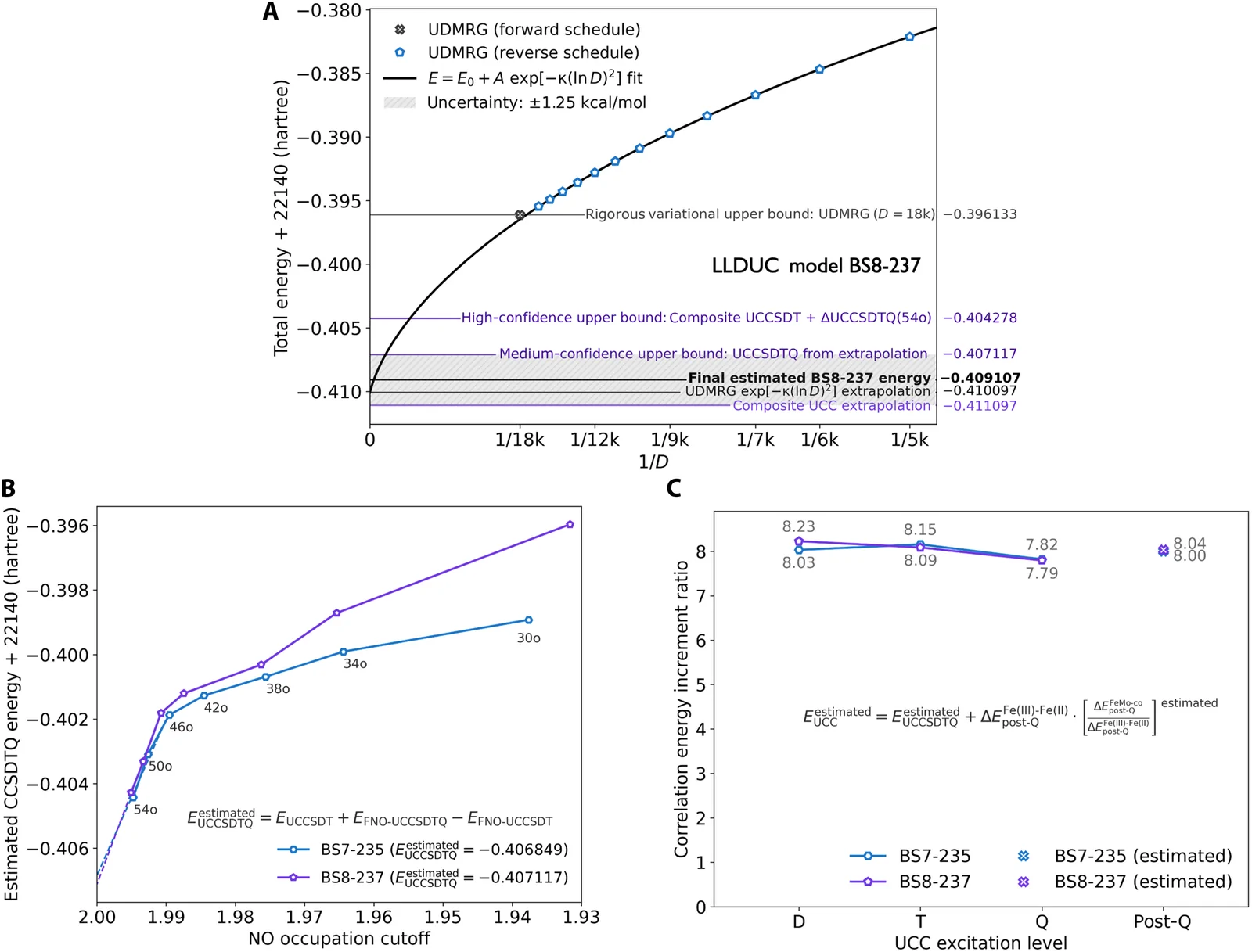
Ground-state energy estimation of the FeMo-co active space model. (**A**) The $\Delta E∼\text{exp}\left[-\kappa {\left(\text{log}D\right)}^{2}\right]$ fitting of UDMRG energies for the BS8-237 state in the LLDUC model. The *D* = 18,000 UDMRG energy from forward schedule is not fully converged and is not included in the extrapolation. (**B**) Composite UCCSDT and frozen natural orbital (FNO)-UCCSDTQ energies and extrapolation (using linear fitting for 46o, 50o, and 54o energies) with respect to UCCSD natural orbital occupation cutoff for the BS7-235 and BS8-237 states in the LLDUC model. (**C**) Estimation of the exact UCC energy using correlation energy increment ratios between the Fe(III)-Fe(II) dimer and FeMo-co LLDUC model for the BS7-235 and BS8-237 states.

Our first prediction of the ground-state energy, based on BS8-237, is −22140.4101 hartrees from extrapolating the UDMRG energy. UDMRG energies can typically be extrapolated in one of a few different ways: using the discarded “weight” during the variational calculation (which estimates the remaining error in the wave function) or using a function of the bond dimension $\Delta E∼\text{exp}\left[-\kappa {\left(\text{log}D\right)}^{2}\right]$ (see Supplementary Text for an analysis in the iron cubane). Here, we use the latter approach, whose fit is shown in Fig. 3A.

The UDMRG energy at the largest bond dimension *D* = 18,000 is a strict variational upper bound, about 8.76 kcal/mol above the estimated ground-state energy. Another upper bound is obtained from UCC calculations. Using our resources, we could not carry out UCCSDTQ calculations in the full LLDUC active space but instead used a sequence of smaller active subspaces up to 54 orbitals [chosen using a natural orbital cutoff, i.e., frozen natural orbital (FNO)-UCCSDTQ], together with a composite correction from the UCCSDT energy in the full space, to approximate the UCCSDTQ full active space energy (see Supplementary Text). The convergence of this with respect to the cutoff is shown in Fig. 3B, where it converges to the full UCCSDTQ from above. Our analysis of post-Q (pentuples and beyond) contributions from these FNO calculations and from other FeS clusters shows that it is always either close to zero (i.e., <1 kcal/mol) or negative (e.g., see Supplementary Text). Thus, the UCCSDTQ composite energy from the largest subspace, namely, −22140.4043 hartrees, is (to an uncertainty within chemical accuracy) an upper bound with high confidence. We can further extrapolate the FNO-UCCSDTQ results with respect to the cutoff to estimate the full UCCSDTQ energy. Here, there is strong curvature (connected to the restoration of spin symmetry; see Supplementary Text) that complicates the extrapolation. Using the last three points (Fig. 3B) for the natural orbital extrapolation yields −22140.4071 hartrees. This is the tightest upper bound we produce but with medium confidence, due to the (small amount of) extrapolation.

To predict an exact energy from UCC requires a precise value of the post-Q contributions. We find that in FeMo-co, the D, T, and Q contributions are roughly in a constant ratio ∼ 8 (Fig. 3C) with the corresponding contributions from a mixed-valence Fe-S dimer that serves as a correlation model (see Supplementary Text). Scaling the post-Q correlation of the dimer accordingly yields a FeMo-co post-Q increment of −0.0040 hartrees for a predicted ground-state energy of −22140.4111 hartrees.

The UCC- and UDMRG-predicted exact energies are in good agreement, and as a consensus value, we can take the average, obtaining *E*_0_ = −22140.4106 ± 0.0005 hartrees (or ±0.31 kcal/mol). However, as there is no formal theory of these extrapolations, we can also take the more conservative view that the energy lies somewhere between the UCC empirical upper bound and the lowest extrapolated energy, i.e., [−22140.4071, −22140.4111] hartrees or *E*_0_ = −22140.4091 ± 0.0020 hartrees (or ±1.25 kcal/mol). Using a similar procedure (see Supplementary Text) for BS7-C, we obtain *E*_0_ = −22140.4089 ± 0.0021 hartrees (or ±1.29 kcal/mol). Considering the above estimates and uncertainties, we thus conclude that we predict the ground-state energy with an error ∼ chemical accuracy for both spin isomers. Within this accuracy, we find BS7-235 and BS8-237 (and potentially BS8-236; see Supplementary Text) to be degenerate.

### Cost of classical methods and quantum advantage

Given this classical estimation of the FeMo-co ground-state energy to chemical accuracy, we now place it in the context of quantum resource estimates. Many discussions of quantum versus classical costs compare the hypothetical runtime of the classical algorithm on a leading supercomputer to the hypothetical quantum execution time. While we have not run our calculations using a large supercomputer, we follow the analogous procedure to contextualize our costs. These estimates do not account for important practical factors of implementation, but as these are (usually) ignored in other estimates, we also do not consider them here.

The most expensive steps in the classical energy estimate arise from generating the data to extrapolate the UCCSDTQ composite upper bound and the UDMRG energies. The largest UCCSDTQ calculation took ∼400 CPU core hours per iteration and 100 iterations to converge to 10^−8^ hartrees, totaling 40,000 CPU core hours. For the variational UDMRG simulations, a single *D* = 18,000 sweep took 317,440 CPU core hours (aggregated over multiple nodes), and we performed two sweeps. Including the cost of extrapolation using the 12 points at *D* = 16,000, 15,000,…, and 5000, the total UDMRG cost was then 2,768,994 CPU core hours.

We can also estimate the cost of higher accuracy calculations. A variational UDMRG estimate to an accuracy of 1 kcal/mol with respect to the extrapolated UDMRG energy requires *D* ∼ 393,000. Using the time-scaling *T* ∼ *D*^3.05^ (see Supplementary Text), this requires 4.08 × 10^9^ CPU core hours (for two sweeps). Similarly, a UCCSDTQ calculation in the full LLDUC space, using the leading scaling (allowing frozen orbitals to be partially occupied, with the observed occupied orbital scaling $T∼N_{\text{occ}}^{6.58}$; see Supplementary Text), would use 8.94 × 10^5^ CPU core hours (assuming 100 iterations).

We now translate the above into the theoretical ideal cost of running the calculations on the Frontier supercomputer, shown in Fig. 4. For this, we estimated the number of floating point operations (FLOP) associated with the above calculations and divided them by the number of FLOP achievable per second (from combined CPU + GPU resources) on the Frontier supercomputer (1.102 exaFLOPS). Using this metric, the calculations take from 1.90 s for the largest UCCSDTQ calculation in this work and 46.3 s for the UDMRG extrapolations to 21.0 s for the estimated full space UCCSDTQ calculation (assuming no memory limitations and no usage of index-permutation symmetry) and 22.9 hours for two sweeps in a variational UDMRG calculation with *D* = 393,000. These can be compared to a recent quantum algorithm resource estimate of 8.6 hours using sum-of-squares spectral amplification and quantum phase estimation to reach accuracy of 1 kcal/mol, assuming *O*(1) overlap with the initial state (*13*). In Supplementary Text, we show that the overlap of the filtered broken-symmetry determinant with the *D* = 18,000 UDMRG state is as large as 0.4468; this should be interpreted as the overlap with the manifold of eigenstates within 1 kcal/mol of the ground-state. This comparison is a snapshot in time: The classical resource cost will certainly decrease in the future, as will the cost of the quantum algorithms.

**Fig. 4.**
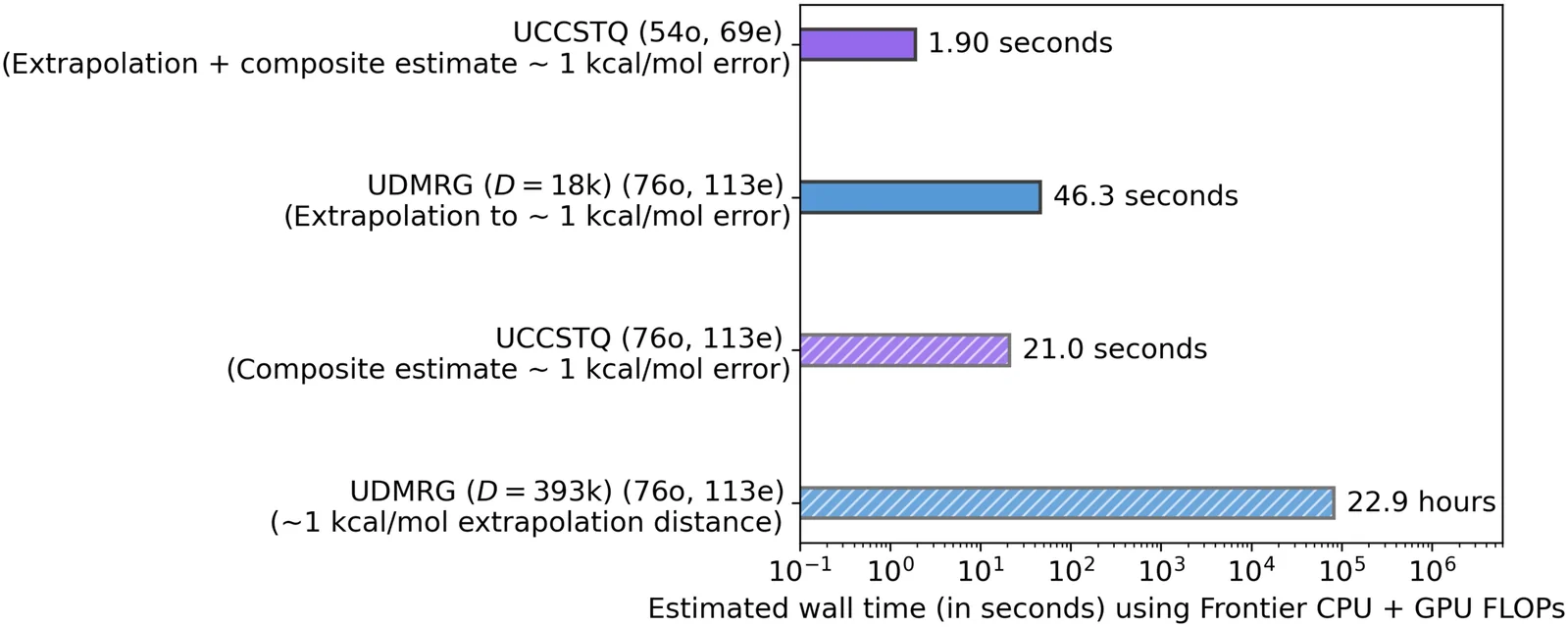
Computational cost and scaling of classical methods. Hypothetical wall time cost of UCCSDTQ and UDMRG for the LLDUC model assuming efficient use of the total combined CPU and GPU resources on the Frontier supercomputer.

### Beyond the LLDUC model and comparisons to spectroscopy

The classical solution of the LLDUC model inspires hope that a precise computational understanding of FeMo-co is possible. However, the small scale of the energy landscape raises questions regarding the robustness of our findings when the model limitations are lifted. The two main limitations are (i) lack of electron correlation outside of the 76-orbital active space (dynamical correlation effects) and (ii) geometric fluctuations of the cluster and protein environment.

In the LLDUC model, although reaching a precision of 1 kcal/mol required substantial effort, qualitative features of the landscape could already be obtained by ranking mean-field solutions based on relatively affordable broken-symmetry CC methods. For example, BS7 and BS8 correctly appear as the lowest energy spin isomer classes (see Fig. 2C) at the UCCSD level (although the degeneracy of BS7-235, BS8-236, and BS8-237 expands to a spread of ∼5 kcal/mol, which may be taken as the ordering uncertainty of the method). At the UCCSDT level, the ordering of the families is consistent with large bond dimension UDMRG (see Supplementary Text). Consequently, to obtain qualitative insights beyond the LLDUC model, we use a simplified protocol, ranking mean-field solutions at the UCCSD level, with additional energies recomputed at the UCCSD(T) level. For properties beyond the energy, we also verified this protocol against UCCSDT and UDMRG data (see Supplementary Text).

We first consider the effect of the protein environment and its fluctuations (see Fig. 5A). Within a QM/MM setup, we included the heterotetramer of the Mo-Fe protein with water solvation and, using a custom force field, ran molecular dynamics to equilibrate the MM environment (see Supplementary Text). From the MD trajectories (*50*), we then obtained the average electrostatic environment produced by protein fluctuations. Figure 5B shows the relative energies of the states in the averaged electrostatic potential for the 76-orbital active space compared to the LLDUC model with its continuum solvation. The low-energy landscape is qualitatively unchanged, and in particular, BS7, BS8, and BS10 remain the lowest energy spin isomer classes (additional discussion of effects from different cluster geometries is in Supplementary Text).

**Fig. 5.**
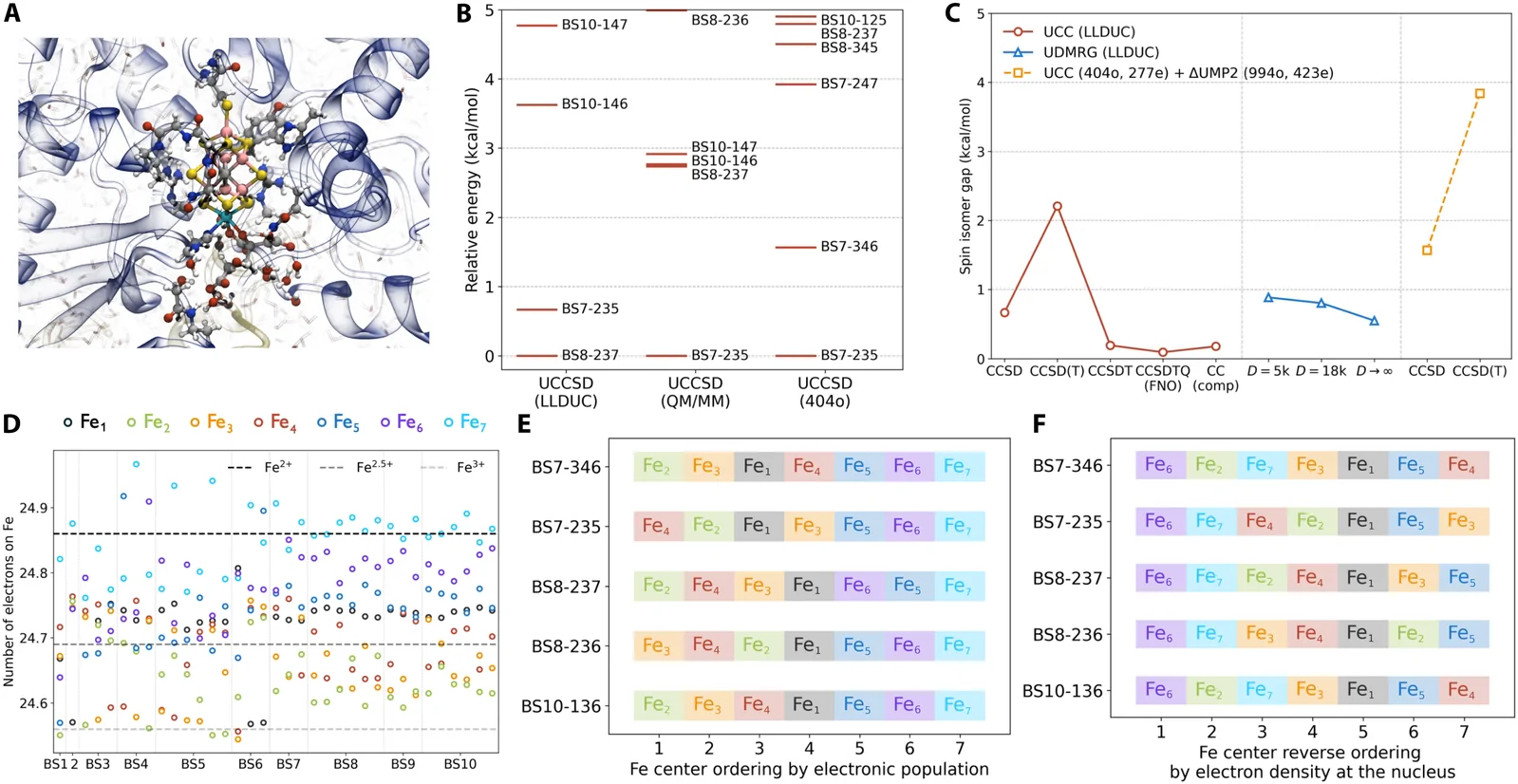
Beyond the LLDUC model and Fe oxidation states. (**A**) Geometry used for the QM/MM simulation [built from PDB 3U7Q (*6*)]. QM and MM atoms are shown as balls and transparent colors, respectively. (**B**) UCCSD relative energies computed using the LLDUC model (with continuum solvation), the 76-orbital active space derived from the QM/MM model with the MD averaged potential, and the larger (404o, 277e) active space with UMP2 composite correction for low-energy spin isomers. (**C**) Energy gap between two lowest-energy spin isomers computed using different levels of UCC and UDMRG theories for the LLDUC and the larger (404o, 277e) active space models. (**D**) Number of electrons on Fe from UCCSD in the (285o, 217e) active space using meta-Löwdin populations without Rydberg contributions. The Fe^3+^, Fe^2+^, and Fe^2.5+^ scales were computed following the procedure in Supplementary Text. (**E**) Ordering of Fe centers (oxidized to reduced) in FeMo-co by number of electrons on Fe from UCCSD in the 404-orbital active space, using meta-Löwdin populations without Rydberg contributions, for low-energy spin isomers (see Supplementary Text for more details). (**F**) Ordering of Fe centers (oxidized to reduced) in FeMo-co by reverse ordering of the electron density at the Fe nucleus from UCCSD in the 404-orbital active space for low-energy spin isomers (see Supplementary Text for more details).

We next consider electron correlation beyond the active space. Starting from the geometry (and continuum solvation treatment) of the LLDUC model, we used a Møller-Plesset second-order perturbation theory (MP2) (*51*) natural orbital truncation to construct an active space of size (404o, 277e), localized to have overlap with the FeMo-co core. In the (404o, 277e) active space, we carried out a full ranking of the Hartree-Fock references using UCCSD energies (as many as 24 references per spin isomer, for a total of 432 references for BS7 to BS10; see Supplementary Text). Figure 5B shows the UCCSD relative energies (with composite UMP2 corrections for the natural orbital truncation) of the spin isomers, and again we find the BS7, BS8, and BS10 to be the lowest in energy.

As summarized earlier, there is strong experimental evidence from (*28*) that constrains the Fe1 spin to be aligned with the cluster total spin (*28*). This rules out the BS10 family as the ground state, but is consistent with BS7 or BS8 as the ground state. BS8 does not appear as a low-energy spin isomer in most prior density functional calculations (see Supplementary Text) and hence has not been considered in analyzing the experimental data. Figure 5C shows that, similarly to in the LLDUC model, the gap between the lowest spin isomers is small in the 404 orbital active space: ∼1.6 kcal/mol at the UCCSD level. Thus, at room temperature or lower, there is a population of different spin isomers, consistent with the unusual temperature dependence of the Mössbauer spectra, which has been posited to arise from a population shift (*28*). Although BS8 and BS10 lie somewhat higher in energy in the 404 orbital model, taking into account the ordering uncertainty of ∼5 kcal/mol from UCCSD, we also cannot rule them out from being thermally accessible after a more accurate correlation treatment. We do not consider the UCCSD(T) relative spin isomer energies to be more reliable than those of UCCSD due to the (T) reference sensitivity (see Supplementary Text for list of UCCSD(T) energies). However, at both the UCCSD and UCCSD(T) level in the 404 orbital active space, we find BS7-235 to be the lowest energy isomer.

Our computations also give insight into the charge structure of the ground state and current experimental interpretations. Using a reference compound approach to assign oxidation states from the valence population density (see Supplementary Text), most of the Fe′s in FeMo-co have some mixed valence character (Fig. 5D), which may relate to the low-energy scale associated with different spin isomers, as the charge distributions are similar. BS1 to BS6 have a wider spread of oxidation states (and thus more localized charges) than BS7 to BS10, which, given the low energies of BS7, BS8, and BS10, suggests that the cluster favors delocalized charges. However, different common measures of assigning oxidation state lead to different conclusions. Figure 5 (E and F) compares the ordering of Fe′s (from oxidized to reduced) using the electron density at the nucleus (related to the Mössbauer isomer shift) and the Fe valence population. These measures are usually assumed (and often found to be) anticorrelated, but we do not find a good anticorrelation in the FeMo-co cluster (see Supplementary Text for further discussion). What oxidation state we assign the Fe centers experimentally and computationally depends on which part of the atomic density on the ion we are probing, and this may resolve potentially conflicting experimental deductions (see Supplementary Text).

We focus here on using the valence population to assign oxidation state. Here, broad features of the ordering are reasonably robust across the spin isomers. For example, across the BS7 and BS8 isomers, Fe2, Fe3, and Fe4 are more oxidized, while Fe5, Fe6, and Fe7 are more reduced. The Mo oxidation state is found to be close to Mo(III) (see Supplementary Text). The fluctuation of the Fe oxidation state across the spin isomers also yields an energy scale for shuttling charge across the cofactor. For example, in the low-energy BS7-235 and BS8-237 spin isomers, the charge of Fe2 differs by 0.3. This sensitivity suggests that the spectroscopic assignment of oxidation state can depend on the conditions under which the cofactor is studied. Last, the energetic accessibility of multiple redox active sites supports the multielectron functionality of the cofactor.

## DISCUSSION

Our work has addressed several aspects of FeMo-co electronic structure. From the viewpoint of the difficulty of ground-state simulation, our work has produced a classical prediction of the ground-state energy to within an estimated chemical accuracy in a model widely used in quantum resource estimates. This enables quantitative assessment of potential quantum advantage and establishes the difficulty of the FeMo-co active space problem relative to other computational problems, such as factoring, where classical and quantum costs are known. Our protocol required extrapolations whose uncertainty can be removed in the future using either improved classical or quantum calculations. The cost estimates we provide for such tasks should be viewed as upper bounds, as we expect further algorithm improvements. Classical methods succeed here because of insight into the structure of the states. It is expected that future quantum heuristics will use these insights also.

From the viewpoint of the chemistry and spectroscopy of the FeMo-co, our work highlights the dense electronic manifold of Fe-S clusters first identified in the Fe-cubane and now confirmed across the different clusters of nitrogenase. The small energy scale has a clear impact on interpreting spectroscopic measurements, as small changes in different experiments may affect the electronic state being measured. At the same time, our work supports the use of broken-symmetry theoretical approaches that capture the physical effect of orbital and spin-dependent localization, substantially simplifying the computation of the low-energy manifold.

For the purposes of understanding the catalytic mechanism of nitrogenase, computing the ground-state energy is only the starting point. Our work indicates that simpler single-reference electronic structure methods, which are practical to use in realistic representations of the cofactor, may suffice. This is because the complexity of the low-energy electronic structure lies not in the description of intrinsically multiconfigurational effects but the correct ranking of many competing, but largely single-configuration, states. This thus provides a feasible path to resolve the ambiguities of current DFT studies.

Substantial effort has already been directed toward using lower accuracy electronic structure methods in conjunction with QM/MM to elucidate the nitrogenase reaction (*25*, *52*). Building on our protocols, we now have a viable path forward where accurate and computationally practical quantum data can be combined with machine learning to improve on such studies (*53*). This presents the possibility of future high-accuracy computational reaction mechanism modeling and discovery in the long-standing nitrogenase problem.

## MATERIALS AND METHODS

### Filtering protocol for the LLDUC model

To obtain a reliable description of the LLDUC model (especially for the UCC methods), we first performed a thorough sampling of UHF solutions (78,750 initial guesses) and selected broken-symmetry states (24 per spin isomer) with low UHF energies. We then computed the UCCSD energies of these 840 UHF solutions and selected 12 to 24 configurations per spin isomer for the UCCSDT calculations, and furthermore, higher-order calculations were performed on one reference state for BS7-235, BS8-237, and BS8-236. The reference dependence of the UCCSD, UCCSDT, and UDMRG energies and properties is studied in Supplementary Text.

### Broken-symmetry DMRG

We used UDMRG with a broken-symmetry orbital basis (split-localized UCCSD natural orbitals computed for the selected reference) to study the low-energy states of the LLDUC model. We determined the orbital ordering by first pairing α and β orbitals to maximize overlap within paired orbitals and used a genetic algorithm to reorder the orbital pairs (see Supplementary Text). We used a sweep-based deterministic algorithm to compute the coefficient of the dominant determinant (see Supplementary Text). We implemented UDMRG in the open-source distributed parallelized DMRG code in block2 through its Python API (*54*, *55*). We used the $\Delta E∼\text{exp}\left[-\kappa {\left(\text{log}D\right)}^{2}\right]$ fitting for UDMRG energy extrapolation, based on 12 energies obtained from the reverse schedule with bond dimension of 5000 to 16,000 (see Supplementary Text). We verified the reliability of the $\Delta E∼\text{exp}\left[-\kappa {\left(\text{log}D\right)}^{2}\right]$ fitting using the smaller 4Fe-4S active space model (see Supplementary Text).

### Broken-symmetry CC theory

We computed UCC solutions based on broken-symmetry references. For the full active space with 76 orbitals, we computed UCCSD, UCCSD(T), and UCCSDT energies. We performed UCCSDTQ in smaller active subspaces (with up to 54 orbitals) for BS7-235, BS8-237, and BS8-236, where the active subspaces were determined by freezing occupied UCCSD natural orbitals (FNO) (see Supplementary Text). For the smallest 30-orbital model, we performed UCCSDTQP to check the accuracy of UCCSDTQ and estimate pentuple contributions. For subspaces with up to 50 orbitals, we performed UDMRG with *D* = 8000 and energy extrapolation to check the accuracy of UCCSDTQ, and we estimated pentuple contributions and beyond using the ratio of correlation energy increments (see Supplementary Text). For the UCC and FNO-UCC calculations, we developed a new open-source arbitrary-order CC code hast-ucc based on PySCF (*56*), with arbitrary order particle density matrices, where the CC diagrams and intermediates were derived automatically on the fly. We implemented a shared-memory parallel tensor contraction library, with special optimizations for the efficient storage and contraction of high-order UCC amplitude tensors (see Supplementary Text).

### Recovering the total spin

We studied the energy gap between the BS families, spin gap, and total spin behavior in UCC and UDMRG in FeMo-co and [2Fe-2S] and [4Fe-4S] (see Supplementary Text) (*22*, *57*). For FeMo-co, we also constructed a toy Heisenberg model using UCCSD(T) data computed for the LLDUC model, assuming *C*_3_ geometry and a fixed assignment of oxidation states (see Supplementary Text). Through these studies, we verified the validity of spontaneous symmetry breaking at the chemical accuracy scale and the evolution of broken-symmetry spin isomers from the pure spin isomers.

### Noncollinear broken-symmetry states

We investigated the relevance of noncollinear spin symmetry breaking in the LLDUC model at the generalized Hartree-Fock (GHF), generalized spin CCSD (GCCSD), and generalized spin CCSD(T) levels of theory. We identified hundreds of unique GHF and GCCSD solutions and studied the spin configuration similarity between the noncollinear low-energy GHF solutions and representative UHF solutions (see Supplementary Text).

### Active space model based on QM/MM potential

Using a QM/MM setup (*58*–*60*), we modeled the heterotetramer of the Mo-Fe protein with water solvation, using the Universal Force Field (*61*) for FeMo-co with charges derived from GFN2-xtb (*59*) and the rest of the system described by the Amber14 force field (*62*) with TIP3P-FB water (*63*). The electrostatic potential was accumulated from 300-ns MD trajectories (see Supplementary Text). We then constructed a 76-orbital active space model with the QM/MM potential and performed UHF, UCCSD, and UCCSD(T) calculations in the active space. For reference filtering, we also projected unique UHF solutions from the LLDUC model as initial guesses (see Supplementary Text).

### Larger active space models with dynamical correlation

We first determined a (994o, 423e) active space including all FeMo-co cluster orbitals, based on localized spin-unrestricted natural orbitals computed using the high-spin DFT solution. We then truncated the active space to four smaller subspaces with 117 to 404 orbitals, based on UMP2 natural orbital occupation thresholds. We performed UHF, UCCSD, and UCCSD(T) calculations in the truncated active spaces. For reference filtering, we additionally considered projected unique UHF solutions from other truncated active spaces as the initial guesses. We computed the UMP2 composite correction as the difference between the UMP2 energies in the (994o, 423e) and (404o, 277e) active spaces. To determine the UHF reference for UMP2 in the (994o, 423e) active space, we used the projected reference corresponding to the lowest UCCSD energy in the (404o, 277e) active space as the initial guess (see Supplementary Text).

### Oxidation state calibration

We studied the assignment of Fe and Mo oxidation states based on the electron density at the metal nucleus and using meta-Löwdin population analysis (*64*). The latter constructs an orthogonal basis with well-defined core, valence, and Rydberg character, and we considered only the electron population in the core and valence space (namely, without Rydberg contributions). We obtained the electron population scale for different Fe oxidation states using UCCSD performed for different [2Fe-2S] clusters and checked its transferability to the P-cluster using DFT. For the determination of the electron population scale for Mo(III), we use UCCSD performed on [MoFe_3_S_4_(SH)_6_]^3−^ (see Supplementary Text) (*17*, *65*).

### Cluster geometry dependence

We checked the dependence of the energies, iron electron populations, and nucleus electron densities on the FeMo-co cluster geometry using broken-symmetry DFT (see Supplementary Text).

## Supplementary Materials

### This PDF file includes:

Supplementary Text Tables S1 to S33 Figs. S1 to S31 References

## References

[1] H. Beinert, R. H. Holm, E. Münck, Iron-sulfur clusters: Nature’s modular, multipurpose structures. Science 277, 653–659 (1997). 10.1126/science.277.5326.653 9235882

[2] K. Tanifuji, Y. Ohki, Metal–sulfur compounds in N_2_ reduction and nitrogenase-related chemistry. Chem. Rev. 120, 5194–5251 (2020). 10.1021/acs.chemrev.9b00544 32459087

[3] J. Kim, D. C. Rees, Crystallographic structure and functional implications of the nitrogenase molybdenum–iron protein from *Azotobacter vinelandii*. Nature 360, 553–560 (1992). 10.1038/360553a0 25989647

[4] O. Einsle, F. A. Tezcan, S. L. A. Andrade, B. Schmid, M. Yoshida, J. B. Howard, D. C. Rees, Nitrogenase MoFe-protein at 1.16 Å resolution: A central ligand in the FeMo-cofactor. Science 297, 1696–1700 (2002). 10.1126/science.1073877 12215645

[5] H.-I. Lee, P. M. C. Benton, M. Laryukhin, R. Y. Igarashi, D. R. Dean, L. C. Seefeldt, B. M. Hoffman, The interstitial atom of the nitrogenase FeMo-cofactor: ENDOR and ESEEM show it is not an exchangeable nitrogen. J. Am. Chem. Soc. 125, 5604–5605 (2003). 10.1021/ja034383n 12733878

[6] T. Spatzal, M. Aksoyoglu, L. Zhang, S. L. A. Andrade, E. Schleicher, S. Weber, D. C. Rees, O. Einsle, Evidence for interstitial carbon in nitrogenase FeMo cofactor. Science 334, 940–940 (2011). 10.1126/science.1214025 PMC3268367 22096190

[7] K. M. Lancaster, M. Roemelt, P. Ettenhuber, Y. Hu, M. W. Ribbe, F. Neese, U. Bergmann, S. DeBeer, X-ray emission spectroscopy evidences a central carbon in the nitrogenase iron-molybdenum cofactor. Science 334, 974–977 (2011). 10.1126/science.1206445 PMC3800678 22096198

[8] M. Reiher, N. Wiebe, K. M. Svore, D. Wecker, M. Troyer, Elucidating reaction mechanisms on quantum computers. Proc. Natl. Acad. Sci. U.S.A. 114, 7555–7560 (2017). 10.1073/pnas.1619152114 PMC5530650 28674011

[9] Z. Li, J. Li, N. S. Dattani, C. J. Umrigar, G. K.-L. Chan, The electronic complexity of the ground-state of the FeMo cofactor of nitrogenase as relevant to quantum simulations. J. Chem. Phys. 150, 024302 (2019). 10.1063/1.5063376 30646701

[10] K. Wan, M. Berta, E. T. Campbell, Randomized quantum algorithm for statistical phase estimation. Phys. Rev. Lett. 129, 030503 (2022). 10.1103/PhysRevLett.129.030503 35905339

[11] D. W. Berry, C. Gidney, M. Motta, J. R. McClean, R. Babbush, Qubitization of arbitrary basis quantum chemistry leveraging sparsity and low rank factorization. Quantum 3, 208 (2019).

[12] M. Luo, J. I. Cirac, Efficient simulation of quantum chemistry problems in an enlarged basis set. PRX Quantum 6, 010355 (2025).

[13] G. H. Low, R. King, D. W. Berry, Q. Han, A. E. DePrince, A. F. White, R. Babbush, R. D. Somma, N. C. Rubin, Fast quantum simulation of electronic structure by spectral amplification. Phys. Rev. X 15, 041016 (2025).

[14] C. Van Stappen, L. Decamps, G. E. Cutsail III, R. Bjornsson, J. T. Henthorn, J. A. Birrell, S. De Beer, The spectroscopy of nitrogenases. Chem. Rev. 120, 5005–5081 (2020). 10.1021/acs.chemrev.9b00650 PMC7318057 32237739

[15] E. Münck, H. Rhodes, W. H. Orme-Johnson, L. C. Davis, W. J. Brill, V. K. Shah, Nitrogenase. VIII. Mössbauer and EPR spectroscopy. The MoFe protein component from *Azotobacter vinelandii* OP. Biochim. Biophys. Acta 400, 32–53 (1975). 10.1016/0005-2795(75)90124-5 167863

[16] R. Björnsson, F. Neese, S. DeBeer, Revisiting the mössbauer isomer shifts of the FeMoco cluster of nitrogenase and the cofactor charge. Inorg. Chem. 56, 1470–1477 (2017). 10.1021/acs.inorgchem.6b02540 28071903

[17] R. Björnsson, F. A. Lima, T. Spatzal, T. Weyhermüller, P. Glatzel, E. Bill, O. Einsle, F. Neese, S. De Beer, Identification of a spin-coupled Mo(III) in the nitrogenase iron–molybdenum cofactor. Chem. Sci. 5, 3096–3103 (2014).

[18] J. K. Kowalska, J. T. Henthorn, C. van Stappen, C. Trncik, O. Einsle, D. Keavney, S. DeBeer, X-ray magnetic circular dichroism spectroscopy applied to nitrogenase and related models: Experimental evidence for a spin-coupled molybdenum (III) center. Angew. Chem. Int. Ed. Engl. 58, 9373–9377 (2019). 10.1002/anie.201901899 PMC6772009 31119827

[19] H. E. Stanley, T. A. Kaplan, High-temperature expansions-the classical Heisenberg model. Phys. Rev. Lett. 16, 981–983 (1966).

[20] T. Lovell, R. A. Torres, W. G. Han, T. Liu, D. A. Case, L. Noodleman, Metal substitution in the active site of nitrogenase MFe7S9 (M= Mo^4+^, V^3+^, Fe^3+^). Inorg. Chem. 41, 5744–5753 (2002). 10.1021/ic020474u 12401079

[21] L. Cao, U. Ryde, Influence of the protein and DFT method on the broken-symmetry and spin states in nitrogenase. Int. J. Quantum Chem. 118, e25627 (2018).

[22] Z. Li, G. K.-L. Chan, Spin-projected matrix product states: Versatile tool for strongly correlated systems. J. Chem. Theory Comput. 13, 2681–2695 (2017). 10.1021/acs.jctc.7b00270 28467847

[23] Z. Li, S. Guo, Q. Sun, G. K.-L. Chan, Electronic landscape of the P-cluster of nitrogenase as revealed through many-electron quantum wavefunction simulations. Nat. Chem. 11, 1026–1033 (2019). 10.1038/s41557-019-0337-3 31570817

[24] S. Lee, J. Lee, H. Zhai, Y. Tong, A. M. Dalzell, A. Kumar, P. Helms, J. Gray, Z. H. Cui, W. Liu, M. Kastoryano, R. Babbush, J. Preskill, D. R. Reichman, E. T. Campbell, E. F. Valeev, L. Lin, G. K. L. Chan, Evaluating the evidence for exponential quantum advantage in ground-state quantum chemistry. Nat. Commun. 14, 1952 (2023). 10.1038/s41467-023-37587-6 PMC10082187 37029105

[25] B. Benediktsson, R. Björnsson, QM/MM study of the nitrogenase MoFe protein resting state: Broken-symmetry states, protonation states, and QM region convergence in the FeMoco active site. Inorg. Chem. 56, 13417–13429 (2017). 10.1021/acs.inorgchem.7b02158 29053260

[26] L. Cao, U. Ryde, Extremely large differences in DFT energies for nitrogenase models. Phys. Chem. Chem. Phys. 21, 2480–2488 (2019). 10.1039/c8cp06930a 30652711

[27] S. J. Yoo, H. C. Angove, V. Papaefthymiou, B. K. Burgess, E. Münck, Mössbauer study of the MoFe protein of nitrogenase from *Azotobacter vinelandii* using selective ^57^Fe enrichment of the M-centers. J. Am. Chem. Soc. 122, 4926–4936 (2000).

[28] E. D. Badding, S. Srisantitham, D. A. Lukoyanov, B. M. Hoffman, D. L. M. Suess, Connecting the geometric and electronic structures of the nitrogenase iron–molybdenum cofactor through site-selective ^57^Fe labelling. Nat. Chem. 15, 658–665 (2023). 10.1038/s41557-023-01154-9 PMC10710871 36914792

[29] J. T. Henthorn, R. J. Arias, S. Koroidov, T. Kroll, D. Sokaras, U. Bergmann, D. C. Rees, S. DeBeer, Localized electronic structure of nitrogenase FeMoco revealed by selenium K-edge high resolution x-ray absorption spectroscopy. J. Am. Chem. Soc. 141, 13676–13688 (2019). 10.1021/jacs.9b06988 PMC6716209 31356071

[30] T. Spatzal, J. Schlesier, E. M. Burger, D. Sippel, L. Zhang, S. L. A. Andrade, D. C. Rees, O. Einsle, Nitrogenase FeMoco investigated by spatially resolved anomalous dispersion refinement. Nat. Commun. 7, 10902 (2016). 10.1038/ncomms10902 PMC4793075 26973151

[31] Z. Li, Entanglement-minimized orbitals enable faster quantum simulation of molecules. Phys. Rev. Lett. 135, 210601 (2025). 10.1103/bwvc-z9hz 41349105

[32] S. Sharma, K. Sivalingam, F. Neese, G. K.-L. Chan, Low-energy spectrum of iron–sulfur clusters directly from many-particle quantum mechanics. Nat. Chem. 6, 927–933 (2014). 10.1038/nchem.2041 25242489

[33] J. M. Montgomery, D. A. Mazziotti, Strong electron correlation in nitrogenase cofactor, FeMoco. J. Phys. Chem. A 122, 4988–4996 (2018). 10.1021/acs.jpca.8b00941 29771514

[34] Z. Li, Expressibility of comb tensor network states (CTNS) for the P-cluster and the FeMo-cofactor of nitrogenase. Electron. Struct. 3, 014001 (2021).

[35] C. Xiang, W. Jia, W.-H. Fang, Z. Li, Distributed multi-GPU ab initio density matrix renormalization group algorithm with applications to the P-cluster of nitrogenase. J. Chem. Theory Comput. 20, 775–786 (2024). 10.1021/acs.jctc.3c01228 38198503

[36] J. Brabec, J. Brandejs, K. Kowalski, S. Xantheas, Ö. Legeza, L. Veis, Massively parallel quantum chemical density matrix renormalization group method. J. Comput. Chem. 42, 534–544 (2021). 10.1002/jcc.26476 33377527

[37] A. Menczer, M. van Damme, A. Rask, L. Huntington, J. Hammond, S. S. Xantheas, M. Ganahl, Ö. Legeza, Parallel implementation of the Density Matrix Renormalization Group method achieving a quarter petaFLOPS performance on a single DGX-H100 GPU node. J. Chem. Theory Comput. 20, 8397–8404 (2024). 10.1021/acs.jctc.4c00903 PMC11465466 39297788

[38] S. R. White, Density matrix formulation for quantum renormalization groups. Phys. Rev. Lett. 69, 2863–2866 (1992). 10.1103/PhysRevLett.69.2863 10046608

[39] S. R. White, Density-matrix algorithms for quantum renormalization groups. Phys. Rev. B 48, 10345–10356 (1993). 10.1103/physrevb.48.10345 10007313

[40] G. K.-L. Chan, M. Head-Gordon, Highly correlated calculations with a polynomial cost algorithm: A study of the density matrix renormalization group. J. Chem. Phys. 116, 4462–4476 (2002).

[41] A. O. Mitrushenkov, G. Fano, F. Ortolani, R. Linguerri, P. Palmieri, Quantum chemistry using the density matrix renormalization group. J. Chem. Phys. 115, 6815–6821 (2001).

[42] S. Wouters, D. Van Neck, The density matrix renormalization group for ab initio quantum chemistry. Eur. Phys. J. D 68, 272 (2014).

[43] S. Keller, M. Dolfi, M. Troyer, M. Reiher, An efficient matrix product operator representation of the quantum chemical Hamiltonian. J. Chem. Phys. 143, 244118 (2015). 10.1063/1.4939000 26723662

[44] A. Baiardi, M. Reiher, The density matrix renormalization group in chemistry and molecular physics: Recent developments and new challenges. J. Chem. Phys. 152, 040903 (2020). 10.1063/1.5129672 32007028

[45] F. Verstraete, T. Nishino, U. Schollwöck, M. C. Bañuls, G. K. Chan, M. E. Stoudenmire, Density matrix renormalization group, 30 years on. Nat. Rev. Phys. 5, 273–276 (2023).

[46] R. J. Bartlett, M. Musiał, Coupled-cluster theory in quantum chemistry. Rev. Mod. Phys. 79, 291–352 (2007).

[47] I. Shavitt, R. J. Bartlett, *Many-body Methods in Chemistry and Physics: MBPT and Coupled-cluster Theory* (Cambridge Univ. Press, 2010).

[48] H. Tasaki, Long-range order, “Tower” of states, and symmetry breaking in lattice quantum systems. J. Stat. Phys. 174, 735–761 (2019).

[49] L. Noodleman, C. Y. Peng, D. A. Case, J.-M. Mouesca, Orbital interactions, electron delocalization and spin coupling in iron-sulfur clusters. Coord. Chem. Rev. 144, 199–244 (1995).

[50] C. Li, G. K.-L. Chan, Accurate QM/MM molecular dynamics for periodic systems in GPU4PySCF with applications to enzyme catalysis. J. Chem. Theory Comput. 21, 803–816 (2025). 10.1021/acs.jctc.4c01698 39813105

[51] J. A. Pople, J. S. Binkley, R. Seeger, Theoretical models incorporating electron correlation. Int. J. Quantum Chem. 10, 1–19 (1976).

[52] L. Cao, O. Caldararu, U. Ryde, Protonation and reduction of the FeMo cluster in nitrogenase studied by quantum mechanics/molecular mechanics (QM/MM) calculations. J. Chem. Theory Comput. 14, 6653–6678 (2018). 10.1021/acs.jctc.8b00778 30354152

[53] C. Li, G. K.-L. Chan, Predictive free energy simulations through hierarchical distillation of quantum Hamiltonians. Proc. Natl. Acad. Sci. U.S.A. 123, e2529120123 (2026). 10.1073/pnas.2529120123 PMC12890790 41642992

[54] H. Zhai, G. K.-L. Chan, Low communication high performance ab initio density matrix renormalization group algorithms. J. Chem. Phys. 154, 224116 (2021). 10.1063/5.0050902 34241198

[55] H. Zhai, H. R. Larsson, S. Lee, Z. H. Cui, T. Zhu, C. Sun, L. Peng, R. Peng, K. Liao, J. Tölle, J. Yang, S. Li, G. K.-L. Chan, BLOCK2: A comprehensive open source framework to develop and apply state-of-the-art DMRG algorithms in electronic structure and beyond. J. Chem. Phys. 159, 234801 (2023). 10.1063/5.0180424 38108484

[56] Q. Sun, X. Zhang, S. Banerjee, P. Bao, M. Barbry, N. S. Blunt, N. A. Bogdanov, G. H. Booth, J. Chen, Z. H. Cui, J. J. Eriksen, Y. Gao, S. Guo, J. Hermann, M. R. Hermes, K. Koh, P. Koval, S. Lehtola, Z. Li, J. Liu, N. Mardirossian, J. D. McClain, M. Motta, B. Mussard, H. Q. Pham, A. Pulkin, W. Purwanto, P. J. Robinson, E. Ronca, E. R. Sayfutyarova, M. Scheurer, H. F. Schurkus, J. E. T. Smith, C. Sun, S. N. Sun, S. Upadhyay, L. K. Wagner, X. Wang, A. White, J. D. Whitfield, M. J. Williamson, S. Wouters, J. Yang, J. M. Yu, T. Zhu, T. C. Berkelbach, S. Sharma, A. Y. Sokolov, G. K.-L. Chan, Recent developments in the PySCF program package. J. Chem. Phys. 153, 024109 (2020). 10.1063/5.0006074 32668948

[57] S. Sharma, G. K.-L. Chan, Spin-adapted density matrix renormalization group algorithms for quantum chemistry. J. Chem. Phys. 136, 124121 (2012). 10.1063/1.3695642 22462849

[58] P. Eastman, R. Galvelis, R. P. Peláez, C. R. A. Abreu, S. E. Farr, E. Gallicchio, A. Gorenko, M. M. Henry, F. Hu, J. Huang, A. Krämer, J. Michel, J. A. Mitchell, V. S. Pande, J. P. G. L. M. Rodrigues, J. Rodriguez-Guerra, A. C. Simmonett, S. Singh, J. Swails, P. Turner, Y. Wang, I. Zhang, J. D. Chodera, G. de Fabritiis, T. E. Markland, OpenMM 8: Molecular dynamics simulation with machine learning potentials. J. Phys. Chem. B 128, 109–116 (2024). 10.1021/acs.jpcb.3c06662 PMC10846090 38154096

[59] C. Bannwarth, S. Ehlert, S. Grimme, GFN2-xTB—An accurate and broadly parametrized self-consistent tight-binding quantum chemical method with multipole electrostatics and density-dependent dispersion contributions. J. Chem. Theory Comput. 15, 1652–1671 (2019). 10.1021/acs.jctc.8b01176 30741547

[60] R. Björnsson, “ASH: A Python-based multiscale modelling program,” in *12th Triennial Congress of the World Association of Theoretical and Computational Chemists* (*WATOC 2020*) (WATOC, 2022).

[61] A. K. Rappé, C. J. Casewit, K. S. Colwell, W. A. Goddard III, W. M. Skiff, UFF, a full periodic table force field for molecular mechanics and molecular dynamics simulations. J. Am. Chem. Soc. 114, 10024–10035 (1992).

[62] J. A. Maier, C. Martinez, K. Kasavajhala, L. Wickstrom, K. E. Hauser, C. Simmerling, ff14SB: Improving the accuracy of protein side chain and backbone parameters from ff99SB. J. Chem. Theory Comput. 11, 3696–3713 (2015). 10.1021/acs.jctc.5b00255 PMC4821407 26574453

[63] L.-P. Wang, T. J. Martinez, V. S. Pande, Building force fields: An automatic, systematic, and reproducible approach. J. Phys. Chem. Lett. 5, 1885–1891 (2014). 10.1021/jz500737m PMC9649520 26273869

[64] Q. Sun, G. K.-L. Chan, Exact and optimal quantum mechanics/molecular mechanics boundaries. J. Chem. Theory Comput. 10, 3784–3790 (2014). 10.1021/ct500512f 26588523

[65] M. Cook, M. Karplus, Electronic structure of the [MoFe_3_S_4_(SH)_6_]^3−^ ion: A broken-symmetry metal–sulfur cluster. J. Chem. Phys. 83, 6344–6366 (1985).

[66] T. Lovell, J. Li, T. Liu, D. A. Case, L. Noodleman, FeMo cofactor of nitrogenase: A density functional study of states MN, MOX, MR, and MI. J. Am. Chem. Soc. 123, 12392–12410 (2001). 10.1021/ja011860y 11734043

[67] H. Zhai, Data for classical quantum chemical solution of the FeMo-cofactor model to Chemical Accuracy and its Implications, Zenodo (2026); 10.5281/zenodo.22116121. PMC13685976 42905822

[68] The PySCF Developers, The PySCF package, version 2.12.0, Zenodo (2026); 10.5281/zenodo.22116646.

[69] H. Zhai, G. K.-L. Chan, block-hczhai/block2-preview (p0.5.4rc16). arXiv:2601.04621, Zenodo (2026); 10.5281/zenodo.22115816.

[70] H. Zhai, G. K.-L. Chan, hczhai/hast-ucc. arXiv:2601.04621, Zenodo (2026); 10.5281/zenodo.22116003.

[71] P. Zanello, Structure and electrochemistry of proteins harboring iron-sulfur clusters of different nuclearities. Part V. Nitrogenases. Coord. Chem. Rev. 398, 113004 (2019). 10.1016/j.jsb.2019.01.003 30677521

[72] H. Q. Huang, M. Kofford, F. B. Simpson, G. D. Watt, Purification, composition, charge, and molecular weight of the FeMo cofactor from azotobacter vinelandii nitrogenase. J. Inorg. Biochem. 52, 59–75 (1993). 10.1016/0162-0134(93)85623-g 8228979

[73] H. Jiang, O. K. Svensson, U. Ryde, Quantum mechanical calculations of redox potentials of the metal clusters in nitrogenase. Molecules 28, 65 (2023). 10.3390/molecules28010065 PMC9822455 36615260

[74] W.-T. Jin, M. Yang, S.-S. Zhu, Z.-H. Zhou, Bond-valence analyses of the crystal structures of FeMo/V cofactors in FeMo/V proteins. Acta Crystallogr. D Struct. Biol. 76, 428–437 (2020). 10.1107/S2059798320003952 32355039

[75] I. Dance, Computational investigations of the chemical mechanism of the enzyme nitrogenase. Chembiochem 21, 1671–1709 (2020). 10.1002/cbic.201900636 31803989

[76] S. Raugei, L. C. Seefeldt, B. M. Hoffman, Critical computational analysis illuminates the reductive-elimination mechanism that activates nitrogenase for N2 reduction. Proc. Natl. Acad. Sci. U.S.A. 115, E10521–E10530 (2018). 10.1073/pnas.1810211115 PMC6233137 30355772

[77] I. Dance, Survey of the geometric and electronic structures of the key hydrogenated forms of FeMo-co, the active site of the enzyme nitrogenase: Principles of the mechanistically significant coordination chemistry. Inorganics 7, 8 (2019).

[78] M. Torbjörnsson, U. Ryde, Comparison of the accuracy of DFT methods for reactions with relevance to nitrogenase. Electron. Struct. 3, 034005 (2021).

[79] B. Benediktsson, R. Björnsson, Analysis of the geometric and electronic structure of spin-coupled iron–sulfur dimers with broken-symmetry DFT: Implications for FeMoco. J. Chem. Theory Comput. 18, 1437–1457 (2022). 10.1021/acs.jctc.1c00753 PMC8908755 35167749

[80] K. Honjo, T. Kamimura, K. Tada, R. Kishi, Y. Kitagawa, Theoretical study of local environmental effect on electronic structure of M-cluster. Eur. J. Inorg. Chem. 28, e202500016 (2025).

[81] C. Van Stappen, B. Benediktsson, A. Rana, A. Chumakov, Y. Yoda, D. Bessas, L. Decamps, R. Bjornsson, S. De Beer, Structural correlations of nitrogenase active sites using nuclear resonance vibrational spectroscopy and QM/MM calculations. Faraday Discuss. 243, 253–269 (2023). 10.1039/d2fd00174h 37067436

[82] G. K.-L. Chan, A. Keselman, N. Nakatani, Z. Li, S. R. White, Matrix product operators, matrix product states, and ab initio density matrix renormalization group algorithms. J. Chem. Phys. 145, 014102 (2016). 10.1063/1.4955108 27394094

[83] J. Pipek, P. G. Mezey, A fast intrinsic localization procedure applicable for ab initio and semiempirical linear combination of atomic orbital wave functions. J. Chem. Phys. 90, 4916–4926 (1989).

[84] R. Olivares-Amaya, W. Hu, N. Nakatani, S. Sharma, J. Yang, G. K.-L. Chan, The ab-initio density matrix renormalization group in practice. J. Chem. Phys. 142, 034102 (2015). 10.1063/1.4905329 25612684

[85] Q. Sun, T. C. Berkelbach, N. S. Blunt, G. H. Booth, S. Guo, Z. Li, J. Liu, J. D. McClain, E. R. Sayfutyarova, S. Sharma, S. Wouters, G. K.-L. Chan, PySCF: The Python-based simulations of chemistry framework. WIREs Comput. Mol. Sci. 8, e1340 (2018).

[86] Ö. Legeza, G. Fáth, Accuracy of the density-matrix renormalization-group method. Phys. Rev. B 53, 14349–14358 (1996). 10.1103/physrevb.53.14349 9983233

[87] K. Okunishi, Y. Hieida, Y. Akutsu, Universal asymptotic eigenvalue distribution of density matrices and corner transfer matrices in the thermodynamic limit. Phys. Rev. E 59, R6227–R6230 (1999). 10.1103/physreve.59.r6227 11969725

[88] G. K.-L. Chan, P. W. Ayers, E. S. Croot III, On the distribution of eigenvalues of grand canonical density matrices. J. Stat. Phys. 109, 289–299 (2002).

[89] D. W. Berry, Y. Tong, T. Khattar, A. White, T. I. Kim, G. H. Low, S. Boixo, Z. Ding, L. Lin, S. Lee, G. K.-L. Chan, R. Babbush, N. C. Rubin, Rapid initial-state preparation for the quantum simulation of strongly correlated molecules. PRX Quantum 6, 020327 (2025).

[90] H. Schurkus, D.-T. Chen, H.-P. Cheng, G. K.-L. Chan, J. Stanton, Theoretical prediction of magnetic exchange coupling constants from broken-symmetry coupled cluster calculations. J. Chem. Phys. 152, 234115 (2020). 10.1063/1.5144696 32571049

[91] H. Zhai, S. Lee, Z. H. Cui, L. Cao, U. Ryde, G. K.-L. Chan, Multireference protonation energetics of a dimeric model of nitrogenase iron–sulfur clusters. J. Phys. Chem. A 127, 9974–9984 (2023). 10.1021/acs.jpca.3c06142 PMC10694817 37967028

[92] S. Lee, H. Zhai, S. Sharma, C. J. Umrigar, G. K.-L. Chan, Externally corrected CCSD with renormalized perturbative triples (R-ecCCSD (T)) and the density matrix renormalization group and selected configuration interaction external sources. J. Chem. Theory Comput. 17, 3414–3425 (2021). 10.1021/acs.jctc.1c00205 34018739

[93] A. D. Becke, Density-functional exchange-energy approximation with correct asymptotic behavior. Phys. Rev. A 38, 3098–3100 (1988). 10.1103/physreva.38.3098 9900728

[94] C. Lee, W. Yang, R. G. Parr, Development of the Colle-Salvetti correlation-energy formula into a functional of the electron density. Phys. Rev. B 37, 785–789 (1988). 10.1103/physrevb.37.785 9944570

[95] A. D. Becke, A new mixing of Hartree–Fock and local density-functional theories. J. Chem. Phys. 98, 1372–1377 (1993).

[96] F. E. Jorge, G. G. Camiletti, S. F. Machado, Contracted Gaussian basis sets for Douglas-Kroll-Hess calculations: Estimating scalar relativistic effects of some atomic and molecular properties. J. Chem. Phys. 130, 064108 (2009). 10.1063/1.3072360 19222268

[97] E. Cancès, Y. Maday, B. Stamm, Domain decomposition for implicit solvation models. J. Chem. Phys. 139, 054111 (2013). 10.1063/1.4816767 23927247

[98] F. Lipparini, Y. Maday, B. Stamm, E. Cancès, B. Mennucci, Fast domain decomposition algorithm for continuum solvation models: Energy and first derivatives. J. Chem. Theory Comput. 9, 3637–3648 (2013). 10.1021/ct400280b 26584117

[99] F. Lipparini, G. Scalmani, L. Lagardère, B. Stamm, E. Cancès, Y. Maday, J. P. Piquemal, M. J. Frisch, B. Mennucci, Quantum, classical, and hybrid QM/MM calculations in solution: General implementation of the ddCOSMO linear scaling strategy. J. Chem. Phys. 141, 184108 (2014). 10.1063/1.4901304 25399133

[100] A. Damle, L. Lin, L. Ying, Compressed representation of Kohn–Sham orbitals via selected columns of the density matrix. J. Chem. Theory Comput. 11, 1463–1469 (2015). 10.1021/ct500985f 26574357

[101] C. Møller, M. S. Plesset, Note on an approximation treatment for many-electron systems. Phys. Rev. 46, 618–622 (1934).

[102] P. Pyykkö, M. Atsumi, Molecular single-bond covalent radii for elements 1–118. Chem. A Eur. J. 15, 186–197 (2009). 10.1002/chem.200800987 19058281

[103] R. D. Shannon, Revised effective ionic radii and systematic studies of interatomic distances in halides and chalcogenides. Acta Crystallogr. A 32, 751–767 (1976).

[104] P. W. Anderson, An approximate quantum theory of the antiferromagnetic ground state. Phys. Rev. 86, 694–701 (1952).

[105] F. Weigend, R. Ahlrichs, Balanced basis sets of split valence, triple zeta valence and quadruple zeta valence quality for H to Rn: Design and assessment of accuracy. Phys. Chem. Chem. Phys. 7, 3297–3305 (2005). 10.1039/b508541a 16240044

