# Supplementary material for: Classical quantum chemical solution of the FeMo-cofactor model to chemical accuracy and its implications
Source: Sci Adv. 2026 Oct 21;12(43):eaeg7106. doi: 10.1126/sciadv.aeg7106 (PMC13685976; doi:10.1126/sciadv.aeg7106)
Supplement: Supplementary file 1 [file sciadv.aeg7106_sm.pdf]

Science Advances
AAAS

Supplementary Materials for

Classical quantum chemical solution of the FeMo-cofactor model to chemical accuracy and its implications

Huanchen Zhai *et al.*

Corresponding author: Garnet Kin-Lic Chan, gkc1000@gmail.com

Sci. Adv. **12**, eaeg7106 (2026)
DOI: 10.1126/sciadv.aeg7106

This PDF file includes:

Supplementary Text
Tables S1 to S33
Figs. S1 to S31
References

1 Prior work on FeMo-co resting state and nomenclature

1.1 Atomic structure

In this work, we focus on the resting (E_0) state of FeMo-cofactor (FeMo-co) models based on the 1.0-Å crystal structure of nitrogenase from *Azotobacter vinelandii* (PDB 3U7Q) (*6*). The identification of the FeMo-co atomic structure took almost two decades. In 1992, the first crystal structure was reported, where the central atom was missing and all Fe atoms were three-coordinated (*3*). Ten years later, the central atom was identified as a light atom at 1.16-Å resolution (*4*), which was finally determined to be a six-coordinated carbon in 2011, by 1.0-Å high-resolution crystallography. Thereafter, the crystal structures for other nitrogenase bacteria have also been reported, while redox potential studies show that there are no significant variations among the FeMo-co proteins expressed by different bacteria (*71*).

1.2 Spin, charge and oxidation states

The total spin of the FeMo-co ground state was determined by electron paramagnetic resonance (EPR) to be $S = 3/2$ (*15*). The total charge of FeMo-co was experimentally determined to be negative (*72*). For the oxidation states of iron and molybdenum, a study in 2000 based on ^{57}Fe Mössbauer data supported a $[\mathrm{Mo}^{4+}\,3\,\mathrm{Fe}^{3+}\,4\,\mathrm{Fe}^{2+}]^{1-}$ oxidation assignment (*27*). In 2014, Bjornsson et al (*17*). proposed the Mo(III) assignment based on high-energy resolution fluorescence detected Mo K-edge X-ray absorption spectroscopy, which has since been supported by density functional calculations and later experiments (*18*). Based on the Mo(III) assignment, total spin, ^{57}Fe Mössbauer studies, and further DFT calculations, Bjornsson et al (*16*). further proposed an oxidation state assignment of $[\mathrm{Mo}^{3+}\,4\,\mathrm{Fe}^{3+}\,3\,\mathrm{Fe}^{2+}]^{1-}$ in 2017. We use the $1-$ total charge assignment for models in this work, which has also been used by the majority of computational studies of the FeMo-co resting state since 2017 (*9, 21, 25, 73*). Note that compared to the localized formal Fe(II) and Fe(III) charge assignment above, other computations have suggested a picture with more delocalized electrons on the Fe atoms (*25*).

More recent experimental and computational studies have focused on the assignment of oxidation states to individual iron atoms, which is a more challenging problem under active debate. In 2016, spatially resolved anomalous diffraction refinement (SpReAD) analysis of X-ray data suggested that Fe1, Fe3, and Fe7 are the most reduced sites, although the interpretation of the signal is generally considered to be complicated (*30*). A 2019 study based on selenium K-edge high resolution X-ray absorption spectroscopy suggested that Fe2 and Fe6 are more oxidized than Fe3/4/5/7 and form an antiferromagnetic configuration (*29*). In contrast, a 2020 study based on the classical bond-valence analysis of the crystal structure of FeMo-co suggested Fe1, Fe6 and Fe7 should be considered to be Fe(III), while Fe2/3/4/5 are more

mixed-valence (*74*). In 2023, analysis of Mössbauer data with site-selective ^{57}Fe labeling suggested that Fe1 is aligned with the cluster total spin, has a delocalized $\mathrm{Fe}^{2.5+}$ oxidation state, and forms a ferromagnetic configuration with at least one of its neighbor irons (Fe2, Fe3, or Fe4) (*28*). Although there is room for overlap in the above assignments, it is also clear that they are not entirely consistent. In the main text, we give some insight into a possible origin of these variations.

1.3 Density functional studies

1.3.1 Broken-symmetry spin isomers

Early computational studies of FeMo-co were performed by Noodleman and co-workers (*20, 66*), who introduced the BS1-BS10 nomenclature that is widely used in the literature for computational studies of FeMo-co. The notation BSn ($n = 1, \cdots, 10$) is used to label different spin isomers for FeMo-co low-energy states, assuming the approximate 3-fold symmetry through the Fe1-Mo axis. Each spin isomer corresponds to an assignment of four α (up-oriented) large spins and three β (down-oriented) large spins to seven Fe centers. Without the 3-fold symmetry, we have $C_3^7 = 35$ unique spin isomers, where each of them can be labeled by the BSn name and the three integers ijk indicating the iron sites with β spin, assuming the total spin projection is aligned with α. Most recent computational studies use the BSn-ijk or BSijk notation. In this work, we mainly use this convention in the main text, but computationally we also use a simple $0, 1, \cdots, 34$ indexing, and the labeling BSn-X ($X =$ A, B, $\cdots$, E) for brevity in the Supporting Material. The correspondence between these notations is summarized in Supplementary Table 1.

We additionally note that the Fe site numbering in the integral and the XYZ coordinate files published with the LLDUC model (*9*) is different from the conventional experimental structure numbering of Fe atoms (*6, 16*). We used the LLDUC model numbering in the input files of all computations, while for all data presented in the main text and the Supporting Material, we use the experimental structure numbering. The experimental structure labels Fe1, Fe2, Fe3, Fe4, Fe5, Fe6, and Fe7 correspond to Fe1, Fe4, Fe3, Fe2, Fe5, Fe7, and Fe6 in the LLDUC model.

1.3.2 Energy landscape

In 2017, Benediktsson and Bjornsson performed a QM/MM DFT study for the three BS7 states of the FeMo-co resting state, and found that BS7-A(346) is the most stable spin isomer, with BS7-C(235) and BS7-B(247) being 0.7 and 1.1 kcal/mol higher in energy, respectively (*25*). They suggested BS7-C to be the most likely ground state based on a metal–metal distance analysis and comparison with the X-ray structure. Later, Cao and Ryde performed a more comprehensive QM/MM DFT study in 2018 on all 35 broken-symmetry states, for the resting and other intermediates of FeMo-co (*21*). For the resting state they found the three BS7 states to be the most stable, and the BS6 states to be the next most stable, being ~ 6.5 kcal/mol higher in energy. The energy variation among the three BS7 states was

Supplementary Table 1: Correspondence between the notation for broken-symmetry solutions used in this work and Refs. (*16, 21, 75*). The BSn numbering is the nomenclature introduced in (*20*). The three numbers i, j, k after BSn are the experimental structure numbering of Fe atoms with β spin.

spin isomer index	this work	Ref. (*21*)	Refs. (*16, 75*)
0	BS3-A	BS3-3	BS3-134
1	BS3-B	BS3-2	BS3-124
2	BS9-A	BS9-3	BS9-145
3	BS10-A	BS10-6	BS10-147
4	BS10-B	BS10-5	BS10-146
5	BS3-C	BS3-1	BS3-123
6	BS10-C	BS10-3	BS10-135
7	BS9-B	BS9-2	BS9-137
8	BS10-D	BS10-4	BS10-136
9	BS10-E	BS10-1	BS10-125
10	BS10-F	BS10-2	BS10-127
11	BS9-C	BS9-1	BS9-126
12	BS6-A	BS6-2	BS6-157
13	BS6-B	BS6-1	BS6-156
14	BS6-C	BS6-3	BS6-167
15	BS2	BS2	BS2-234
16	BS8-A	BS8-6	BS8-345
17	BS8-B	BS8-4	BS8-347
18	BS7-A	BS7-3	BS7-346
19	BS8-C	BS8-5	BS8-245
20	BS7-B	BS7-2	BS7-247
21	BS8-D	BS8-2	BS8-246
22	BS5-A	BS5-6	BS5-457
23	BS5-B	BS5-5	BS5-456
24	BS4-A	BS4-3	BS4-467
25	BS7-C	BS7-1	BS7-235
26	BS8-E	BS8-3	BS8-237
27	BS8-F	BS8-1	BS8-236
28	BS5-C	BS5-3	BS5-357
29	BS4-B	BS4-2	BS4-356
30	BS5-D	BS5-4	BS5-367
31	BS4-C	BS4-1	BS4-257
32	BS5-E	BS5-1	BS5-256
33	BS5-F	BS5-2	BS5-267
34	BS1	BS1	BS1-567

found to be 0.6 to 1.3 kcal/mol. In their study, BS7-C or BS7-A were found to be the most stable spin isomers when the TPSS-D3 or B3LYP functionals were used, respectively. Another DFT study by Raugei et al (*76*). in 2018 (with the COSMO polarizable dielectric continuum treatment for the protein environment not explicitly considered in their model) for the resting state suggested that BS7-A is the most stable, while other spin isomers were 7 to 50 kcal/mol higher in energy. DFT studies on hydrogenated FeMo-co by Dance in 2019 suggested BS7-A, BS7-B, BS7-C, BS6-A, BS2, BS10-A, and BS10-C to be the low-energy isomers for FeMo-co derivative structures (*75, 77*).

1.3.3 DFT functional dependence and protein environment effects

The 2018 study by Cao and Ryde studied the contribution of different computational factors to the relative energetics. The work suggested that the effects of varying the basis set and the modeling of the protein surroundings contributed up to 2.6 kcal/mol, the treatment of the cluster geometry contributes up to 8.8 kcal/mol, and the effect of the DFT functional (TPSS or B3LYP) is up to 13.9 kcal/mol (*21*). Dispersion corrections were found to be important for obtaining accurate energies (up to 13.6 kcal/mol) in a 2021 study by Torbjörnsson and Ryde (*78*). In 2022, Benediktsson and Bjornsson reported a benchmark set of 11 Fe–Fe and Mo–Fe dimeric complexes, and found that the metal–metal distance is very sensitive to the specific choice of DFT functional (*79*). In a 2025 DFT study, Honjo et al (*80*). reported that hydrogen bonds and water molecules can significantly contribute to the stability of the LUMO and HOMO, for the resting state of FeMo-co.

In 2023, Van Stappen et al (*81*). performed nuclear resonance vibrational spectroscopy experiments for different nitrogenase active sites including FeMo-co, and compared the results with the calculated partial vibrational density of states (PVDOS) using DFT and QM/MM. For FeMo-co they considered the three BS7 states. They found that the computed PVDOS is sensitive to the specific choice of the BS state, and a proper simulation may require accounting for the degeneracy of spin isomers. They also reported the importance of the proper modeling of the protein environment for reproducing the experimental vibrational spectrum (*81*).

1.4 Beyond density functional theory studies

Considering the high sensitivity in the functional choice observed in the DFT studies and the small energy difference among BS states, it is desirable to investigate the performance of wavefunction based methods that can go beyond DFT accuracy for nitrogenase systems. Active space models were constructed and studied using density matrix renormalization group (DMRG) for [2Fe-2S] and [4Fe-4S] clusters in 2014 (*32*), and later for P clusters with different oxidation states in 2019 (*23*). (54o, 54e) and (67o, 65e) active space models for FeMo-co were proposed by Reiher et. al in 2017 in the context of quantum computing resource estimation (*8*). In 2018, Montgomery and Mazziotti studied the correlations in FeMo-co using smaller subspaces of the (54o, 54e) model, using CASSCF and variational 2-RDM

methods with up to a (30o, 30e) subspace (*33*). However, the (54o, 54e) active space model was later shown to be insufficient to capture the open-shell character in the system (*9*). To fix the problem, in 2019 Li et al (*9*). proposed an improved (76o, 113e) active space model (the LLDUC model) for FeMo-co and performed preliminary DMRG studies (with bond dimension up to 2000). In the 2023 study, Lee et al (*24*). revisited the DMRG studies (mainly via the spin-adapted DMRG formalism (*57*)) for [2Fe-2S], [4Fe-4S], P cluster (8Fe), and FeMo-co (LLDUC) active space (*9*) models with a larger bond dimension (up to 7000 for FeMo-co) and reported the DMRG extrapolation results. The results are summarized in Supplementary Figure 1. We see that the DMRG extrapolation distance for FeMo-co is significantly larger than that of smaller sized Fe-S clusters and the P cluster. Very recently, in 2025 Li proposed to apply spin-adapted DMRG with entanglement-minimized orbitals and reported the results for FeMo-co (LLDUC model) using a DMRG bond dimension of up to 10,000 (*31*).

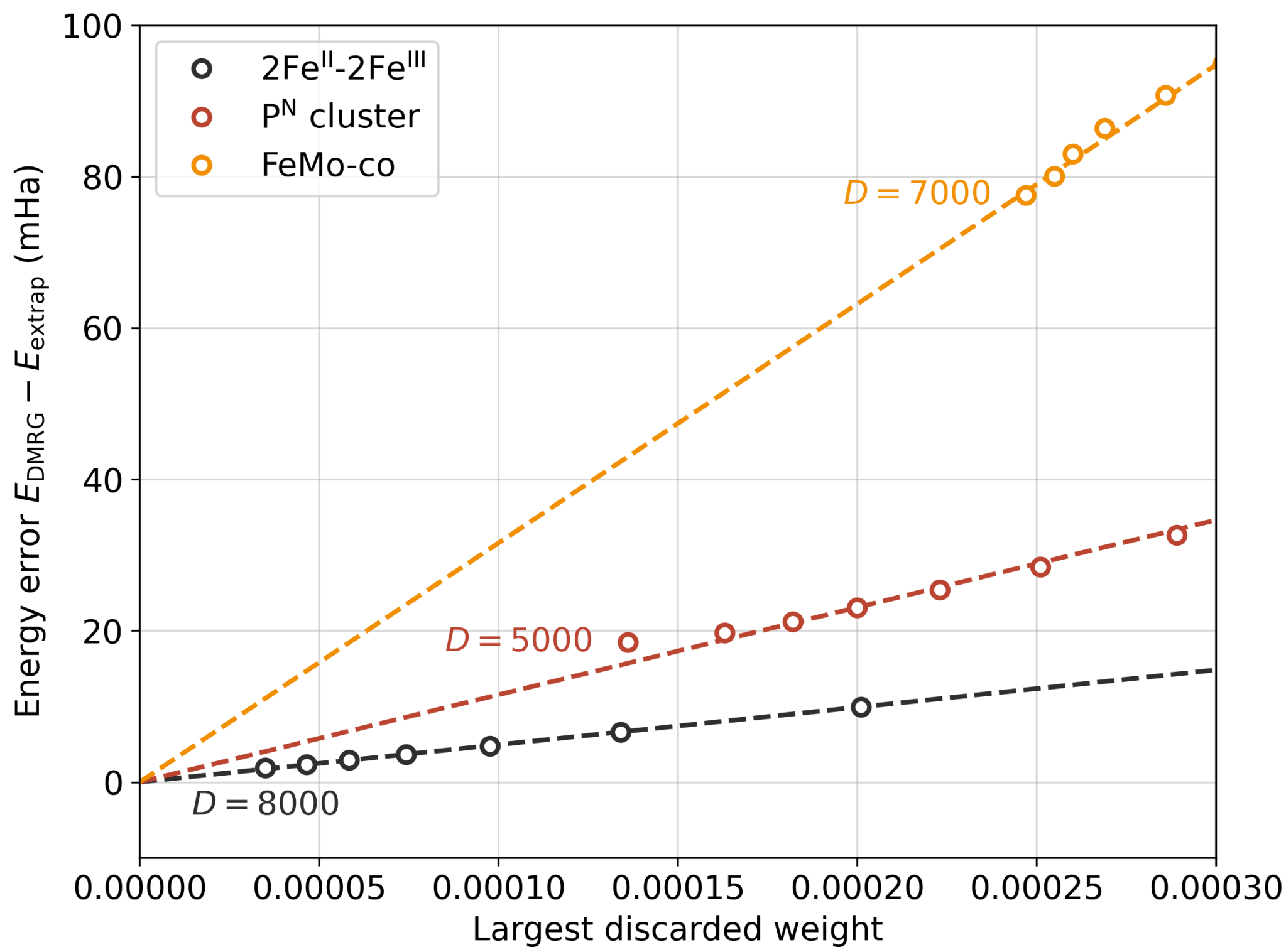

Supplementary Figure 1: **DMRG extrapolation error for** $2\,\mathrm{Fe}^{\mathrm{II}}{-}2\,\mathrm{Fe}^{\mathrm{III}}$ **cluster,** P^{N} **cluster, and FeMo-cofactor.** Data taken from Ref. (*24*).

2 Theory background

2.1 Density matrix renormalization group

The density matrix renormalization group (DMRG) approach (*38, 39, 45*) uses the following ansatz for the wavefunction, called a Matrix Product State (MPS) (*82*)

$$|\Psi\rangle = \sum_{\{n\}} \mathbf{A}[1]^{n_1} \mathbf{A}[2]^{n_2} \cdots \mathbf{A}[K]^{n_K} |n_1\, n_2 \cdots n_K\rangle \tag{S1}$$

where K is the number of spatial orbitals, each n_k is the occupation state on each of the orbitals, and each $\mathbf{A}[k]^{n_k} (k = 2, \cdots, K-1)$ is a $D \times D$ matrix, and $\mathbf{A}[1]^{n_1}$ and $\mathbf{A}[K]^{n_K}$ are vectors. Given a Hamiltonian, an iterative procedure called a sweep algorithm can be used to optimize the $\mathbf{A}$ matrices and find the approximate ground state. The accuracy of the DMRG energy can be systematically improved by increasing the MPS bond dimension D. For quantum chemistry Hamiltonians, one typically uses the spin-adapted formalism of DMRG with spin-restricted (and optionally localized) orbitals, to find the ground state in the correct total spin sector and minimize computational cost (*40, 54, 57*).

For the FeMo-co active space model considered in this work, since the mean-field broken symmetry solutions are good approximations to the low-energy spin isomers, we consider a less conventional spin-unrestricted DMRG (UDMRG) procedure based on broken symmetry orbitals. Specifically, for each of the 35 spin isomers for FeMo-co, we start from one of the broken symmetry UHF solutions and perform spin-unrestricted CCSD (UCCSD) to find the spin-unrestricted CCSD natural orbitals (NO). All UHF and UCCSD calculations are done in the same active space as that for UDMRG. We split-localized the UCCSD NO in four parts: α spin occupied, α spin virtual, β spin occupied, and β spin virtual, using Pipek-Mezey localization (*83*), and used them as the orbitals for UDMRG.

Orbital reordering can have a significant impact on the performance of DMRG (*84*). For the case of UDMRG used in this work, we determine a suitable orbital ordering with the $\alpha\beta\alpha\beta\cdots$ orbital arrangement using a greedy optimization procedure. Specifically, we first find an optimal pairing of α and β orbitals by maximizing the sum of the squared overlaps between paired α and β orbitals. We then use the standard Genetic Algorithm optimization (GAOPT) procedure (*84*) to find a good ordering for the $\alpha\beta$ pairs. To generate a good initial guess for UDMRG, we use the fractional occupation number of the UCCSD NO as a hint for determining the initial quantum number distribution in the MPS (*55*). The UDMRG procedure is implemented using the Python API of BLOCK2 (*54, 55*) interfaced with PySCF (*56, 85*), with support for hybrid distributed and shared-memory parallelization.

2.1.1 Theory of DMRG extrapolation

As the quality of DMRG calculations can be improved systematically by the bond dimension D, it is common to perform an extrapolation of the energy (or other observables) over a series of DMRG calculations with different bond dimensions. There are a variety of extrapolation techniques in the literature. The most common is to extrapolate with respect to the discarded weight $w(D)$, the compression error of the DMRG matrix product state in the variational two-site algorithm. In the limit of large D, one can justify the behaviour $\Delta E(D) \propto w(D)$ where ΔE is the residual energy error (*86*). However, as seen in Sec. 3.1.8, we are not yet in this asymptotic regime in our calculations on the FeMo-co LLDUC model.

An alternative is to directly extrapolate ΔE as a function of the bond dimension D. One form that has been used is

$$\Delta E \sim e^{-\mathrm{const}(\log D)^{\alpha}} \tag{S2}$$

where $\alpha = 2$. This can be derived from the asymptotic behaviour of $w(D)$ for large D and using $\Delta E(D) \propto w(D)$. Under the condition that the entanglement Hamiltonian (logarithm of the system density matrix in DMRG) corresponds to a set of weakly interacting quasiparticles, one obtains a discarded weight of the form Eq. S2, where $\alpha > 1$ is a system specific parameter (*40, 87, 88*). This form has previously been used for energy extrapolation in spin-adapted DMRG calculations on Fe dimers (*24*) and to motivate an extrapolation of the overlap between a small bond dimension MPS and the exact state in Ref. (*89*) also in Fe-S clusters.

Note that while the theoretical derivation of the bond dimension extrapolation also requires $\Delta E \propto w(D)$, as an empirical extrapolation relation, the bond dimension and weight extrapolations may in practice have different regimes of applicability. We use Eq. S2 to carry out bond dimension extrapolation in this work.

2.2 Coupled cluster theory

In the (single reference) coupled cluster (CC) theory we consider the following exponential ansatz (*46, 47*)

$$|\Psi\rangle = \mathrm{e}^{\hat{T}}|\Phi_0\rangle \tag{S3}$$

where $|\Phi_0\rangle$ is a spin-restricted or spin-unrestricted mean-field state, called the reference state in CC theory, and

$$\hat{T} = \hat{T}_1 + \hat{T}_2 + \cdots = \sum_{ia,\sigma} T_{ia,\sigma}\, c^{\dagger}_{a\sigma} c_{i\sigma} + \frac{1}{(2!)^2} \sum_{ijab,\sigma\sigma'} T_{ijab,\sigma\sigma'}\, c^{\dagger}_{a\sigma} c^{\dagger}_{b\sigma'} c_{j\sigma'} c_{i\sigma} + \cdots \tag{S4}$$

is known as the cluster operator, which can be expanded in contributions from single ($\hat{T}_1$), double ($\hat{T}_2$), triple ($\hat{T}_3$), quadruple ($\hat{T}_4$), etc. excitations. Indices $i, j, \cdots$ and $a, b, \cdots$ are used to label occupied and unoccupied orbitals in the reference state $|\Phi_0\rangle$, respectively. For efficient classical simulations, $\hat{T}$ is truncated to include only low-order excitations. For example, in the so-called Coupled Cluster Singles and Doubles (CCSD), we have $\hat{T} = \hat{T}_1 + \hat{T}_2$. We can additionally include an approximate correction for the triples, which gives the commonly used CCSD(T) method.

It has been shown that CC with a spin-unrestricted broken-symmetry reference can provide accurate exchange couplings in bridged transition metal dimers (*90*), while CC with conventional spin-restricted references may potentially have convergence issues for these cases. In our previous work, we have also shown that broken-symmetry CCSD(T) can provide reliable relative energies for the protonation of dimeric models of nitrogenase iron–sulfur clusters (*91*).

For iron–sulfur models studied in this work, one of the difficulties is the large number of broken-symmetry mean-field solutions (typically with different UHF energies). We consider 24 low-energy UHF references (for each spin isomer in FeMo-co) and perform CCSD and CCSD(T) for each of these references. For more expensive theories including CCSDT and DMRG we consider one or a few references with the lowest CCSD energies. The reference dependence of the CC energy and density matrices will be discussed in Supplementary Sec. 3.1.2. For some unfavorable UHF references, broken-symmetry CC can also have convergence difficulties, especially with full triples. In this work we use a large DIIS space (12 to 36) together with normal CC self-consistent iterations (as implemented in PySCF) to alleviate the convergence problem.

For spin-unrestricted CCSDT and CCSDTQ, the large tensor size of the CC amplitude tensor can create a memory bottleneck for systems considered in this work. We developed a memory-efficient arbitrary-order spin-unrestricted CC implementation with support for the contraction of CC tensors in the packed storage format, where only index-symmetry unique elements are kept in each anti-symmetric tensor. The code is parallelized using multi-threading and interfaced with PySCF. To further save the memory cost of CCSDTQ in some large FNO spaces, we also used fully or partially out of core storage for DIIS vectors.

3 Ground state estimation for FeMo-co active space models

3.1 Minimal active space (76o, 113e) LLDUC model

The LLDUC model was defined in Ref. (*9*). The integral (FCIDUMP) file and geometry can be found in `https://github.com/zhendongli2008/Active-space-model-for-FeMoco`. The XYZ coordinates (in Angstroms) of the geometry are listed in Supplementary Listing 1.

Supplementary Listing 1: The XYZ coordinates (in Angstroms) of the geometry used in the LLDUC model.

```
225

Fe 10.018000 -7.568000 56.109001
Fe 12.528000 -8.326000 55.692001
Fe 11.277000 -6.902000 53.853001
Fe 11.990000 -5.771000 56.166000
Fe 14.820000 -7.601000 54.671001
Fe 13.550000 -6.198000 52.848999
Fe 14.226000 -5.072000 55.094002
Mo 16.132000 -5.638000 53.306000
C 13.044000 -6.665000 54.723999
S 11.719000 -7.459000 57.646000
S 16.169001 -6.043000 55.618000
S 10.001000 -5.522000 55.134998
S 13.162000 -3.960000 56.627998
S 14.407000 -9.544000 55.750999
S 14.337000 -4.109000 53.098000
S 10.718000 -8.998000 54.466999
S 15.203000 -7.626000 52.453999
S 11.674000 -6.465000 51.712002
S 8.197000 -8.169000 57.320999
C 13.298000 -2.024000 50.055000
N 12.366000 -2.944000 50.620998
C 11.087000 -2.665000 50.879002
N 10.465000 -1.648000 50.319000
N 10.437000 -3.395000 51.761002
C 15.000000 -1.023000 59.625000
C 16.169001 -0.765000 58.679001
C 17.408001 -1.557000 59.002998
O 17.870001 -1.613000 60.158001
N 18.021999 -2.150000 57.976002
C 8.265000 -1.347000 60.679001
C 9.399000 -1.996000 59.963001
N 9.933000 -3.152000 60.439999
C 10.065000 -1.670000 58.792999
C 10.903000 -3.530000 59.608002
N 10.987000 -2.647000 58.599998
C 8.211000 -4.616000 49.907001
```

C 7.638000 -4.371000 51.291000

C 6.881000 -3.235000 51.567001

C 7.762000 -5.325000 52.312000

C 6.315000 -3.036000 52.811001

C 7.180000 -5.134000 53.570000

C 6.480000 -3.971000 53.827999

O 5.954000 -3.723000 55.074001

N 5.234000 -10.420000 55.743999

C 6.175000 -9.825000 56.689999

C 5.492000 -9.417000 57.998001

O 4.993000 -8.311000 58.167999

C 6.936000 -8.649000 56.117001

N 5.537000 -10.342000 58.952000

C 18.375999 -9.779000 57.895000

O 18.938999 -9.535000 58.952999

N 17.193001 -9.276000 57.611000

C 16.604000 -8.174000 58.390999

C 15.700000 -8.561000 59.548000

O 16.066999 -8.490000 60.719002

N 14.482000 -8.960000 59.175999

C 13.413000 -9.170000 60.129002

C 12.448000 -10.321000 59.819000

O 11.585000 -10.619000 60.638000

N 12.608000 -10.986000 58.669998

C 11.669000 -12.033000 58.242001

C 12.397000 -13.165000 57.548000

O 12.311000 -14.342000 57.938000

N 13.074000 -12.829000 56.459999

C 13.702000 -13.811000 55.596001

C 14.324000 -13.116000 54.386002

C 13.275000 -12.471000 53.491001

C 13.911000 -11.621000 52.422001

N 12.878000 -11.123000 51.516998

C 13.118000 -10.372000 50.460999

N 14.374000 -10.095000 50.109001

N 12.094000 -9.945000 49.705002

O 22.684000 -7.070000 47.084999

C 18.180000 -7.369000 50.759998

C 18.711000 -7.463000 52.162998

N 18.159000 -6.788000 53.228001
C 19.879999 -8.011000 52.627998
C 18.976999 -6.904000 54.275002
N 20.003000 -7.668000 53.919998
C 17.480000 -1.176000 54.216000
C 17.136999 -1.687000 52.838001
C 17.757999 -3.064000 52.515999
C 19.298000 -2.979000 52.311001
C 19.787001 -4.275000 51.637001
C 21.243001 -4.658999 51.755000
C 17.111000 -3.651000 51.271999
O 17.582001 -2.027000 55.126999
O 17.659000 0.041000 54.360001
O 21.753999 -5.267001 50.771999
O 21.844000 -4.408001 52.838001
O 16.628000 -4.810000 51.337002
O 17.166000 -2.989000 50.195999
O 17.490000 -3.979000 53.599998
O 15.888000 -4.907000 46.811001
O 19.811001 -2.775000 44.562000
O 21.295000 -1.675000 56.474998
O 14.797000 -5.542000 49.339001
O 20.999001 -7.558000 49.209999
O 21.018000 -4.234000 48.202000
O 18.273001 -3.799000 47.834000
O 24.264000 -5.111000 53.854000
O 18.688999 -0.721000 49.472000
O 20.888000 -4.190000 55.507000
O 19.867001 0.773000 55.806000
O 19.983999 2.139000 49.183998
O 19.601999 0.653000 51.790001
H 14.253909 -2.551439 49.902245
H 13.485333 -1.148637 50.707842
H 12.976245 -1.666277 49.058362
H 12.756517 -3.749057 51.133080
H 10.948382 -1.006864 49.696509
H 9.418611 -3.350350 51.840380
H 10.851817 -4.301103 52.064489
H 14.176828 -0.309351 59.447134

H 15.333467 -0.925316 60.673517
H 14.594556 -2.042045 59.487892
H 15.865440 -0.939269 57.629728
H 16.465566 0.302217 58.741955
H 17.747578 -2.051739 56.983900
H 18.886142 -2.654352 58.166876
H 7.943033 -0.415330 60.180924
H 8.542154 -1.100693 61.721569
H 7.393391 -2.026762 60.730390
H 11.631869 -2.750219 57.800192
H 9.950464 -0.842408 58.089483
H 11.523221 -4.427460 59.671513
H 8.474673 -3.670967 49.398976
H 7.479231 -5.140569 49.262567
H 9.119194 -5.242955 49.957698
H 6.731721 -2.478799 50.782532
H 8.369443 -6.224645 52.146371
H 5.741910 -2.119104 53.011911
H 7.324840 -5.870775 54.367803
H 5.546271 -2.840425 55.063551
H 5.750165 -10.676231 54.895864
H 4.564723 -9.698382 55.448024
H 6.922962 -10.606213 56.936088
H 6.237039 -7.808570 55.941648
H 7.401622 -8.936745 55.157491
H 6.037121 -11.218482 58.832310
H 5.164447 -10.123761 59.874931
H 18.781655 -10.466135 57.105626
H 16.704789 -9.500843 56.734897
H 16.043744 -7.544498 57.677036
H 17.429698 -7.586986 58.824307
H 14.219156 -8.808225 58.189655
H 12.783401 -8.263772 60.234252
H 13.872680 -9.367031 61.112602
H 13.256381 -10.608473 57.970429
H 11.174293 -12.420930 59.144023
H 10.895271 -11.603903 57.570696
H 13.174849 -11.839973 56.192474
H 14.473224 -14.370958 56.160710

H 12.945500 -14.559086 55.282907
H 15.032972 -12.341634 54.741837
H 14.913117 -13.854269 53.810118
H 12.641772 -13.254547 53.028674
H 12.605520 -11.821374 54.090680
H 14.436696 -10.768534 52.906517
H 14.648390 -12.199351 51.832233
H 11.938028 -11.063509 51.914966
H 14.531875 -9.489172 49.305241
H 15.069805 -9.986501 50.852478
H 12.200422 -9.064588 49.201927
H 23.601459 -6.857982 47.330021
H 22.249074 -6.194096 47.039440
H 18.955188 -7.698977 50.048433
H 17.905390 -6.332504 50.518737
H 17.264032 -7.973340 50.626379
H 20.628332 -8.602807 52.103837
H 18.853442 -6.425326 55.245736
H 16.033435 -1.787606 52.792646
H 17.426546 -0.972948 52.052632
H 19.528063 -2.102232 51.681236
H 19.759202 -2.868484 53.309073
H 19.247123 -5.115528 52.106350
H 19.502098 -4.271284 50.571493
H 16.780810 -4.624223 47.121885
H 15.433213 -5.151276 47.644086
H 19.980766 -3.723341 44.435067
H 19.321145 -2.766364 45.406260
H 21.109337 -1.750623 57.425786
H 20.759935 -0.882264 56.201074
H 15.501236 -5.269415 49.977894
H 14.144043 -5.981686 49.917945
H 21.703906 -7.489930 48.526249
H 21.149790 -6.782293 49.795777
H 21.232006 -4.616723 49.099190
H 21.241122 -3.291069 48.291377
H 19.195384 -4.089918 48.024181
H 17.876980 -3.527801 48.695535
H 24.697333 -5.483950 53.066151

```
H 23.382051 -4.837434 53.479899
H 18.953931 -0.321612 50.327834
H 18.115647 -1.481067 49.723219
H 20.992458 -3.241281 55.753084
H 21.160164 -4.239411 54.564410
H 20.330760 1.130055 55.030296
H 18.979135 0.500481 55.434638
H 19.940635 1.936802 50.139503
H 19.518351 1.363575 48.815374
H 20.354371 0.130718 52.119534
H 18.942524 0.622904 52.521728
H 20.831763 -7.852887 54.530690
H  9.545331 -1.367341 50.646139
H 11.144726 -10.137694 50.018567
C 23.292999 -8.105000 51.974998
C 24.523001 -7.559000 51.272999
O 25.587000 -8.169000 51.252998
N 24.365000 -6.351000 50.728001
C 25.365000 -5.756000 49.866001
C 24.761000 -4.521000 49.146000
O 22.260000 -7.893000 55.609001
H 17.491148 -3.347688 54.445403
H 22.481544 -8.230248 51.233996
H 22.943402 -7.372156 52.723626
H 23.534473 -9.061675 52.460204
H 23.421240 -5.901816 50.778903
H 26.261472 -5.456441 50.446366
H 25.720615 -6.508632 49.132117
H 24.470248 -3.748936 49.879990
H 23.848896 -4.798400 48.587383
H 25.488867 -4.081178 48.443117
H 21.937137 -7.439342 56.409331
H 22.855842 -7.224751 55.210211
```

3.1.1 Non-collinear spin solutions

Since the broken-symmetry UHF solutions have been shown to be useful in describing the low-energy landscape of FeMo-co, one may ask whether further spin symmetry breaking can help. For this purpose we computed the general Hartree-Fock (GHF) solutions and the CCSD and CCSD(T) solutions with the GHF solution as references (termed

GCCSD and GCCSD(T), respectively), for the LLDUC model of FeMo-co. We note that the current implementation of GHF in PySCF can only reliably explore GHF solutions in the real domain. To sample the *real* GHF space, for each of the 35 UHF spin isomers, we considered 2250 possible UHF initial density matrices, but with a small amount of noise to connect the α and β spin manifolds. We solve for the GHF solution using the Newton solver implemented in PySCF, and identified 286 unique GHF solutions for the LLDUC model. The number of unique GHF solutions is significantly smaller than the number of unique UHF solutions, mainly because the 35 UHF spin isomer are no longer "disconnected" in the optimization manifold, and many of them optimize to the same broken-symmetry solution in the GHF space.

The best GHF energy we obtained is -22139.997279 Hartrees, which is 4.3 kcal/mol lower than the best UHF energy (-22139.990392 Hartrees). We computed GCCSD and GCCSD(T) energies using the 286 unique GHF solutions as references, and 221 CCSD calculations converged within 400 iterations. In Supplementary Table 2 we list the GHF, GCCSD, and GCCSD(T) energies for 16 references with the lowest GCCSD energies. The comparison between the UCCSD and GCCSD energies for the low-energy references is shown in Supplementary Figure 2. The best GCCSD energy is 3.9 kcal/mol lower than the best UCCSD energy. In Supplementary Figure 3 we plot the spin orientation in the Fe and Mo atoms (with the spin orientation of Fe1 fixed on the left). We found that half of these low-energy GHF states are small perturbations or copies of the UHF BS states BS7-C, BS8-E and BS8-F. Other low-energy GHF states show clear non-collinear spin configurations, particularly in the right cubane (Fe5, Fe6, and Fe7). We additionally note that the UHF BS states BS7-C, BS8-E and BS8-F have exactly the same spin configuration for Fe1, Fe2, Fe3, and Fe4. As a result, the non-collinear spin arrangement found in the low-energy GHF solutions (such as GHF-64) may be related to the very small energy gap among UHF BS states BS7-C and BS8-E.

Overall, we have not exhaustively studied the GHF solution space to the extent we have characterized the UHF solution space in this work, and further work on GHF solutions is desirable in the future.

3.1.2 Reference dependence

As discussed in Supplementary Sec. 2, we use broken-symmetry mean-field solutions as references for CC and for constructing split-localized broken-symmetry orbitals for DMRG. For each of the 35 spin isomers, there are different ways of assigning the Fe(II) and Fe(III) oxidation states and the doubly occupied orbital in Fe(II). As a result, one would expect that there are many ways to initialize the UHF self-consistent field procedure, and we may end up with different UHF solutions (local minima) for each spin isomer. In this section, we discuss the manifold of these UHF solutions and the consequences of using them as references in broken-symmetry CC and DMRG calculations, respectively.

Supplementary Table 2: Broken-symmetry GHF, GCCSD and GCCSD(T) energies (in Hartrees) computed using different references for the LLDUC model. The GHF references are indexed by their ranking in the GHF energies, counting from zero. The low-energy UHF BS states and their UHF, UCCSD, and UCCSD(T) energies are also listed for comparison.

reference	E_{HF}	E_{CCSD}	$E_{\mathrm{CCSD(T)}}$	similar to
GHF-61	-22139.984067	-22140.312627	-22140.355756	BS7-C (235)
GHF-64	-22139.983413	-22140.308508	-22140.353316	
GHF-21	-22139.990392	-22140.305389	-22140.352783	BS7-C (235)
GHF-63	-22139.983618	-22140.304696	-22140.352173	
GHF-44	-22139.986013	-22140.304318	-22140.352781	BS7-C (235)
GHF-77	-22139.981522	-22140.301346	-22140.348974	
GHF-62	-22139.983774	-22140.301272	-22140.349397	BS8-E (237)
GHF-47	-22139.985676	-22140.300503	-22140.350474	
GHF-33	-22139.987906	-22140.300021	-22140.349813	
GHF-66	-22139.983280	-22140.298387	-22140.346968	BS8-F (236)
GHF-153	-22139.971545	-22140.296702	-22140.355347	BS7-C (235)
GHF-88	-22139.979837	-22140.296366	-22140.345343	
GHF-129	-22139.973956	-22140.296346	-22140.359345	
GHF-187	-22139.967960	-22140.296047	-22140.350789	
GHF-159	-22139.971136	-22140.295958	-22140.349573	
GHF-135	-22139.973441	-22140.295794	-22140.350849	BS7-C (235)
BS7-C	-22139.990392	-22140.305389	-22140.352783	
BS8-E	-22139.981193	-22140.306453	-22140.350447	
BS8-F	-22139.983280	-22140.298387	-22140.346968	

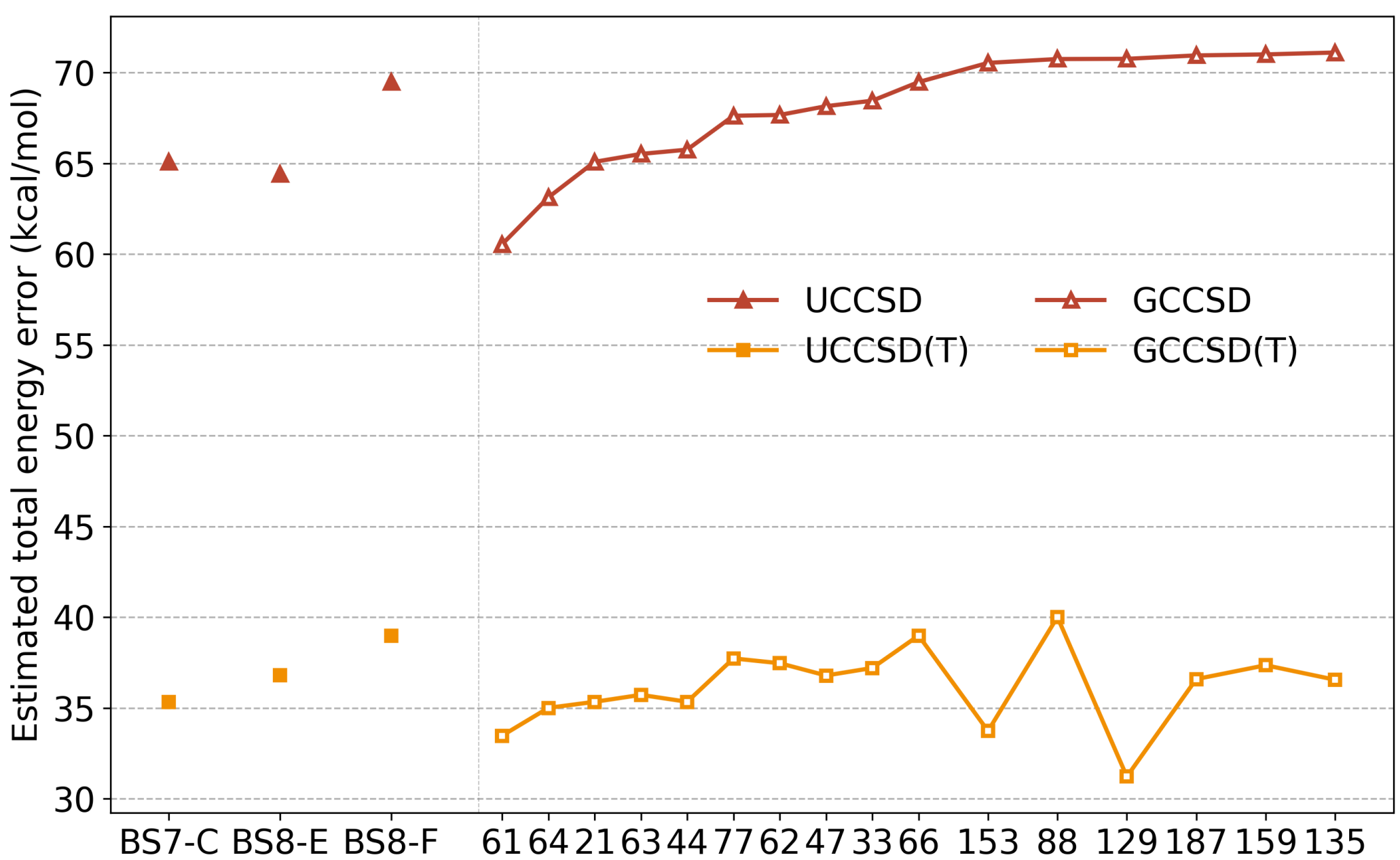

Supplementary Figure 2: **Energy landscape of FeMo-co LLDUC model computed using GCCSD and GCCSD(T), compared with UHF counterparts.** Integers are GHF reference indices based on ranking of GHF energies.

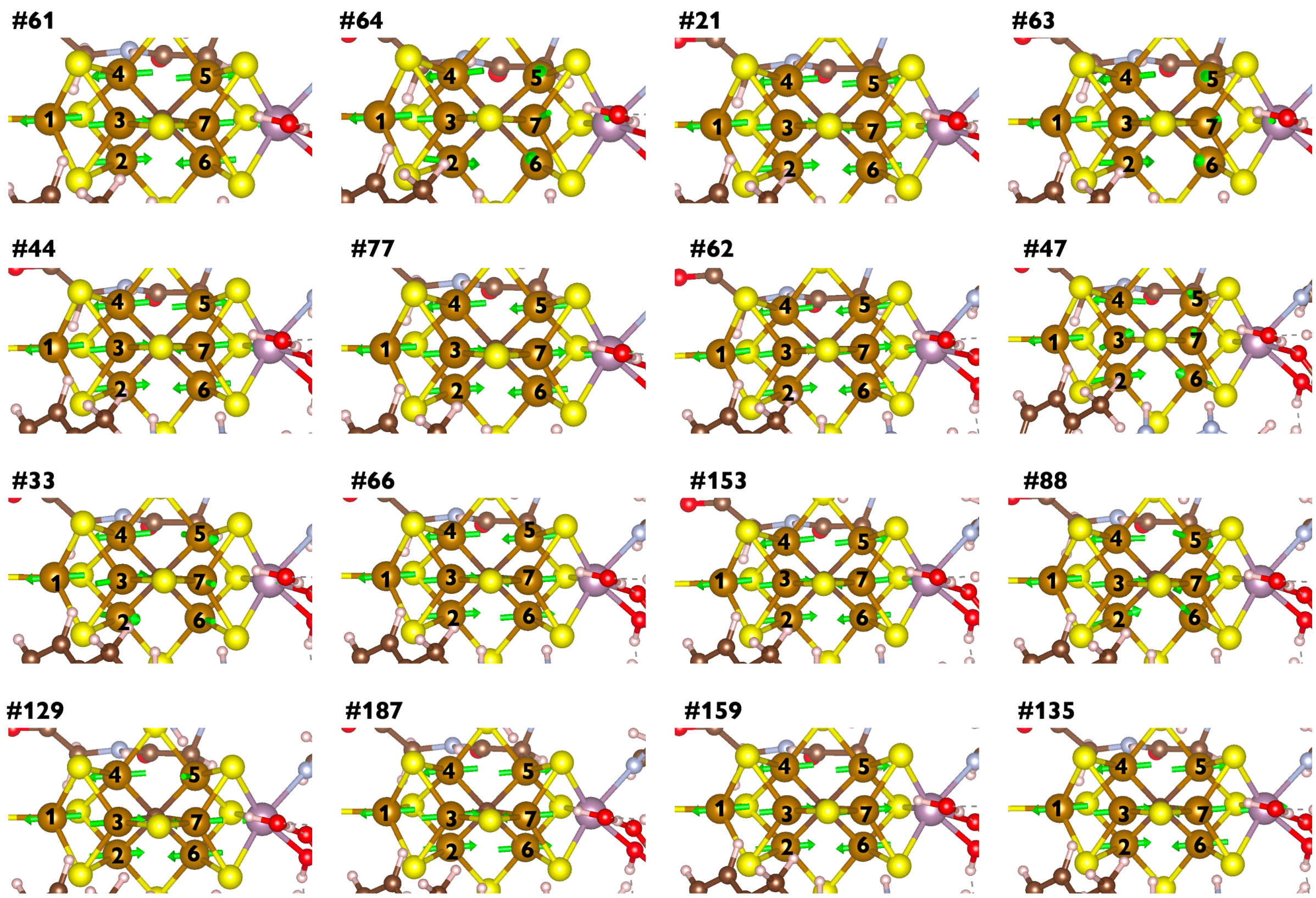

Supplementary Figure 3: **The spin orientation computed using the GHF density matrix for 16 GHF references with the lowest GCCSD energies.** The spin orientation of Fe1 is fixed on the left.

Note that for each given UHF broken-symmetry reference, it is also possible to have multiple CC or DMRG solutions, since the CC and DMRG wavefunctions are obtained from some type of self-consistent iterative algorithm. However, in practice, we do not find this to be a problem for the current study. As long as the algorithm converges, we always end up with a well-defined CC or DMRG solution for each considered UHF reference. A detailed investigation of local minima and convergence behavior in the broken-symmetry CC/DMRG solution manifold is beyond the scope of this work.

Enumeration of UHF solutions. Although the Fe atoms in a converged UHF solution are typically mixed-valence, for the purpose of sampling the low-energy UHF solutions for the LLDUC model, we use density matrices with collinear spins and classical integer Fe(II) and Fe(III) charges (*23*) as the initial guesses for UHF, based on simplicity and ease of reproducibility considerations. As the LLDUC model space is relatively small (compared to the larger active space models used in later sections), and the number of such initial classical density matrices is large ($5^3 \times 18 = 2250$ for each spin isomer), we find that this procedure works very well for identifying the UHF solution with the lowest energy for each spin isomer, as indicated by the relatively flat UHF curve shown in Fig. 2A. For each spin isomer, we found that many of the 2250 initial guesses optimized to the same UHF solution, but the number of unique UHF solutions is still significant, as listed in Supplementary Table 3. The total number of unique UHF solutions we found for this model is 12300, more than the square of the number of unique UHF solutions we found for mixed valence [4Fe-4S], where we found 26 unique UHF solutions (see Supplementary Sec. 4). We label the different UHF solutions within each spin isomer as BSn-X-m where $m = 0, 1, 2, \cdots$ indicates the ordering by UHF energies. Note that the number of unique UHF solutions can also heavily depend on the Newton optimization solver and the counting procedure.

Supplementary Table 3: Number of unique UHF solutions found for each spin isomer in the LLDUC model.

spin isomer	# UHF	spin isomer	# UHF	spin isomer	# UHF	spin isomer	# UHF
BS1	258	BS5-B	313	BS7-B	390	BS9-B	423
BS2	446	BS5-C	280	BS7-C	312	BS9-C	448
BS3-A	286	BS5-D	243	BS8-A	391	BS10-A	285
BS3-B	218	BS5-E	360	BS8-B	417	BS10-B	310
BS3-C	308	BS5-F	294	BS8-C	392	BS10-C	389
BS4-A	364	BS6-A	484	BS8-D	490	BS10-D	378
BS4-B	228	BS6-B	401	BS8-E	290	BS10-E	370
BS4-C	359	BS6-C	342	BS8-F	408	BS10-F	331
BS5-A	379	BS7-A	376	BS9-A	337	**total**	**12300**

For active space models larger than the LLDUC model, the sampling based on only the integer assignment of charges in the electronic configurations may not be sufficient. We additionally consider the projection of unique UHF solutions between different active space models. Details are discussed in Supplementary Sec. 3.2.

Reference dependence: CC energies. In Supplementary Figure 4 we show the dependence of CCSD, CCSD(T), and CCSDT energies on the specific choice of low-energy UHF references, for some selected spin isomers. As broken-symmetry CC calculations are expensive, to reduce the computational cost we performed CCSD and CCSD(T) for 24 references with the lowest UHF energies. For CCSDT we considered 12-24 references with the lowest UHF energies. We can see that for BS10-F, BS6-A, and BS8-E, the reference with the lowest UHF energy (labeled as BS10-F-0, BS6-A-0, and BS8-E-0, respectively) is not the same as the reference with the lowest UCCSD or UCCSDT energy, which indicates that there is a non-trivial reference dependence for UCCSD solutions. The references with the lowest CC energy are BS10-F-20, BS6-A-13, and BS8-E-4 for UCCSD, and BS10-F-12, BS6-A-4, and BS8-E-23 for UCCSDT, respectively. In particular, in some cases the best UCCSD/UCCSDT solution is found with a relatively high energy UHF reference. Nevertheless, it seems to be sufficient to sample a finite number of low-energy UHF references to find CC solutions that are *close enough* to the best CC solution. It can also be seen from Supplementary Figure 4 that the UCCSDT energies mostly track the relative UCCSD energies of different references within each given spin isomer, but with a smaller spread of energies, indicating a weaker reference dependence at higher excitation levels of CC. At a very high accuracy level (mHa), the CCSD and CCSDT energy ordering can be slightly different. But this reordering is actually within the energy uncertainty of the CCSDT approximation itself.

The CCSD(T) energies also track the CCSD energies as the reference is varied for a given spin isomer. The T1 diagnostic is on average large (~ 0.1) indicating that (T) may not be entirely reliable. An ideal method should yield the same energy regardless of reference, for a given spin isomer. Comparing the range of energies across different references (for each given spin isomer), we see that it increases going from CCSD to CCSD(T), highlighting the stronger reference dependence of the (T) correction. For this reason, although the (T) correction does not seem pathological in the LLDUC model, we do not consider it to be more reliable than CCSD (and indeed it may be more unreliable) for giving the ordering and energy differences of spin isomers. In the case of BS8-E-15, the effect of the large (T) correction is partially removed in UCCSDT, again suggesting the importance of the inclusion of full triples for achieving quantitative accuracy for such systems.

Based on the above observation of the reference dependence, in this work, we performed UCCSD and UCCSD(T) for 24 references with the lowest UHF energy for each spin isomer, and report the minimum energy obtained at each level of theory. For UCCSDT calculations we consider 12 references with the lowest UHF energies (including the reference with the lowest UCCSD energy), for each spin isomer (except for BS7-C, BS8-E, and BS8-F, for which 24 references are considered).

Reference dependence: DMRG energies. The dependence of DMRG energies on the UHF references is computed

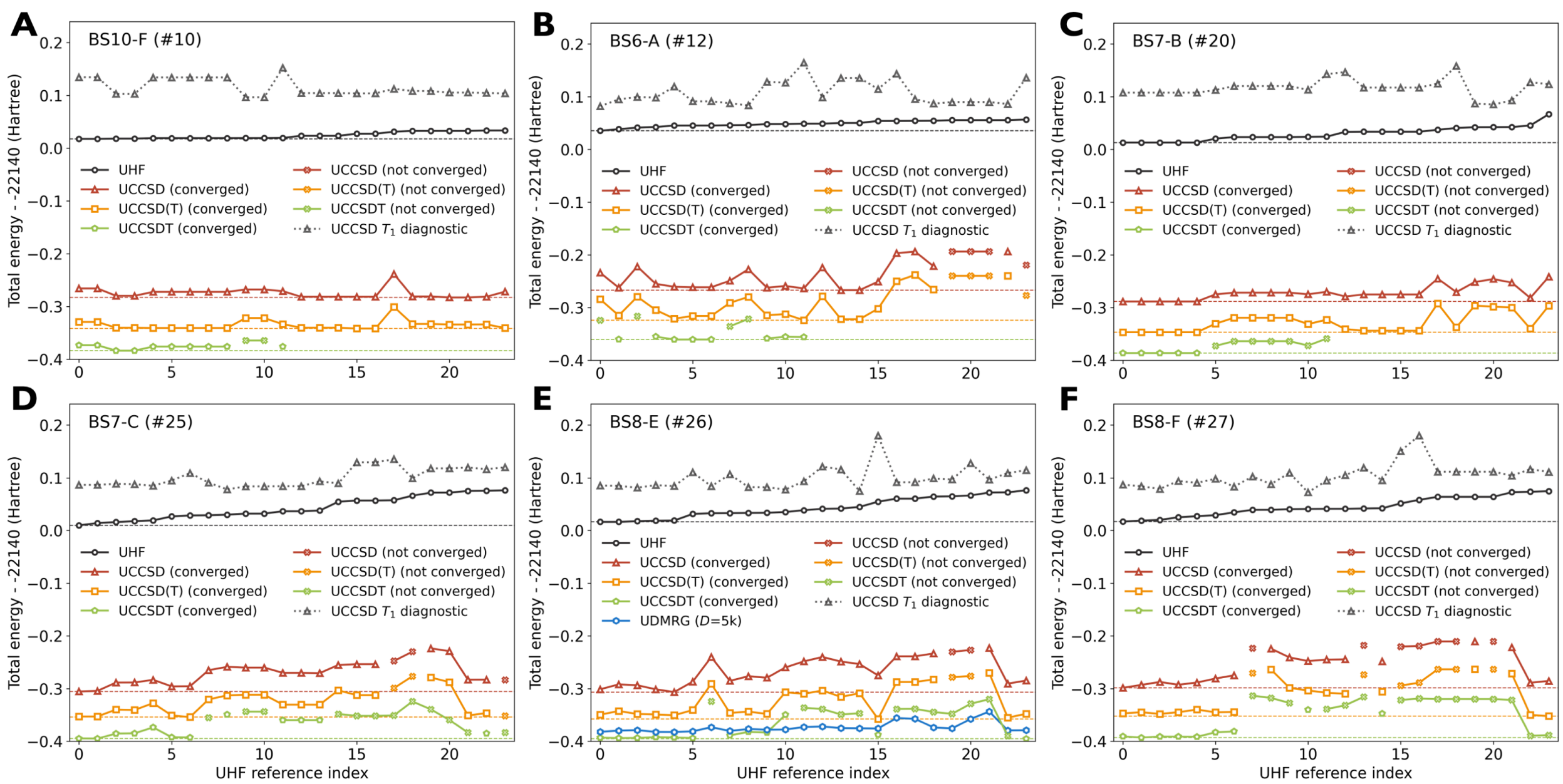

Supplementary Figure 4: **UHF, UCCSD, UCCSD(T), and UCCSDT energies and UCCSD T_1 diagnostic for different references in selected spin isomers (A) BS10-F, (B) BS6-A, (C) BS7-B, (D) BS7-C, (E) BS8-E, and (F) BS8-F, in the LLDUC model.** For BS8-E, UDMRG energies (with bond dimension 5000) for the references with low CCSD energies are also shown. Dashed lines indicate the minimal energy obtained for the considered references at each level of theory.

for BS8-E (for 24 references with the lowest UHF energies and bond dimension 5000) and shown in Supplementary Figure 4E. Compared to UCCSD, we see that UDMRG is less dependent on the particular choice of reference. This is expected because the DMRG ansatz is more flexible for capturing multireference effects in these systems; for example, it can freely reoccupy different orbitals. The UHF reference affects the DMRG calculations only indirectly via the dependence of the UCCSD natural orbitals on the reference, and the initial quantum number distribution which is determined from the UCCSD natural occupations. Because of the reduced dependence on the UHF reference, for the large bond dimension DMRG computations performed in this work, we only considered one reference with the best UHF energy for BS7-C and BS8-E, respectively.

Reference dependence: CC and DMRG properties. We additionally study the reference dependence of the state properties (electron density at Fe nucleus and electron population on Fe) using the one-particle density matrices computed using UCCSD and UDMRG. The results for BS8-E are shown in Supplementary Figure 5. We see that UDMRG nuclear densities are visibly insensitive with respect to the change of reference, and for the electron population on Fe, its reference dependence is also much smaller than that of UCCSD. For both UCCSD and UDMRG, the ordering of the valence density is mostly unchanged when different references are used, while the ordering of the electron density at Fe nucleus (related to the Mössbauer isomer shift) is more robust with respect to the change of reference.

3.1.3 UCC and UDMRG energies

In Supplementary Table 4 we list the UHF, UCC, and UDMRG energies with the best reference for each spin isomer of the FeMo-co LLDUC model. The UHF energy is the minimum among all sampled references, the UCCSD energy is the minimum among 24 references with the lowest UHF energies, the UCCSD(T) energies are obtained using the same reference as the one for UCCSD, and the UCCSDT energy is the minimum among 12 references with the lowest UHF energies and the reference with the lowest UCCSD energy. Additionally, for BS7-C, BS8-E, and BS8-F we computed UCCSDT energies for all 24 low-UHF-energy references and took the minimum. In Supplementary Figure 6 we show the spin isomer relative energies computed using different theories. We see the gap between low-energy spin isomers (especially between BS7-C and BS8-E) decreases when the correlation level increases, showing the importance of a high accuracy treatment of electron correlation in the LLDUC model.

3.1.4 Recovering the total spin

While we have shown that broken-symmetry CC and DMRG theories are effective at finding low-energy states for FeMo-co, the states do not have pure spin. (As discussed in Sec. 7.2.1, these broken-symmetry states live in the manifold of a “tower of states” for each spin isomer). In this section, we perform a detailed analysis on the the effect of the broken-symmetry approximation, and how the pure total spin is recovered at large excitation order (for CC) and

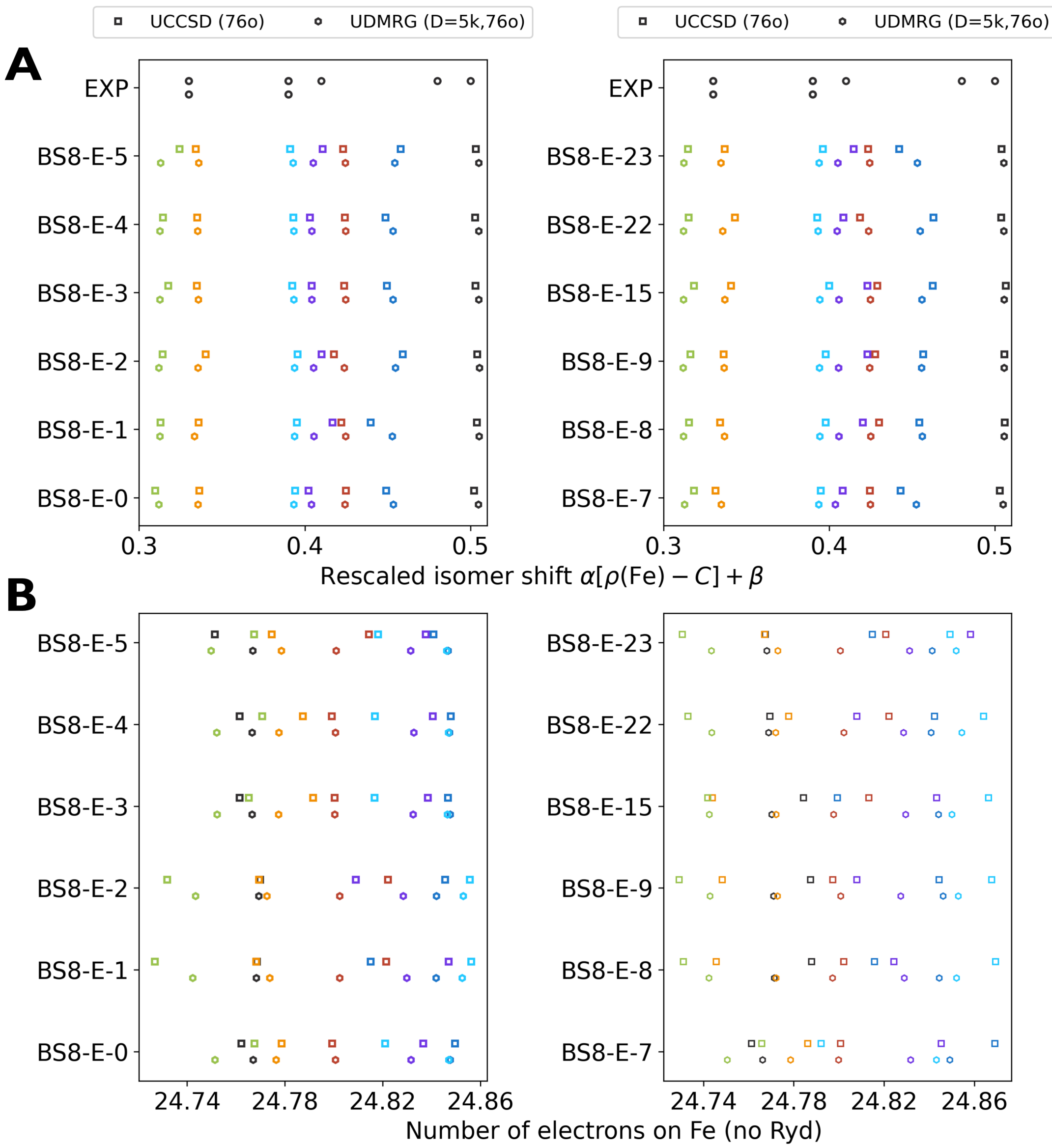

Supplementary Figure 5: **UCCSD and UDMRG (A) rescaled isomer shift (computed using the electron density at the Fe nucleus) and (B) electron population on Fe (computed using meta-Löwdin populations without Rydberg contributions) for different references in spin isomer BS8-E in the LLDUC model.**

Supplementary Table 4: UHF, UCC, and UDMRG energies with the best reference for each spin isomer of the FeMo-co LLDUC model. Energies are in Hartrees, shifted by -22140.0 Hartrees.

spin isomer	E_{UHF}	E_{UCCSD}	$E_{\mathrm{UCCSD(T)}}$	E_{UCCSDT}	$E_{\mathrm{UDMRG}}^{D=5000}$	$E_{\mathrm{UDMRG}}^{D=8000}$	CCSD T_1 diagnostic
BS3-A	0.024129	-0.275898	-0.320929	-0.360651	-0.349340		0.08332
BS3-B	0.026386	-0.275446	-0.327962	-0.361219	-0.350934		0.07482
BS9-A	0.011646	-0.285676	-0.333989	-0.383278	-0.366400		0.09446
BS10-A	0.010505	-0.298846	-0.346108	-0.394618	-0.380355		0.09817
BS10-B	0.010424	-0.300672	-0.352890	-0.394707	-0.380576	-0.385609	0.09116
BS3-C	0.037725	-0.265054	-0.311283	-0.348382	-0.341913		0.08548
BS10-C	0.015230	-0.277971	-0.347828	-0.377909	-0.363839		0.11822
BS9-B	0.021474	-0.271003	-0.327126	-0.370578	-0.356186		0.11668
BS10-D	0.017884	-0.283249	-0.350147	-0.384475	-0.368422		0.10524
BS10-E	0.015563	-0.281324	-0.339846	-0.380163	-0.366238		0.08382
BS10-F	0.017734	-0.283147	-0.334302	-0.383722	-0.370353		0.10529
BS9-C	0.024564	-0.271147	-0.338945	-0.373028	-0.359321		0.09221
BS6-A	0.035623	-0.267123	-0.322240	-0.360736	-0.344611		0.13550
BS6-B	0.038193	-0.269801	-0.317138	-0.361635	-0.348310		0.09217
BS6-C	0.039963	-0.276379	-0.322975	-0.362158	-0.349788		0.08872
BS2	0.025921	-0.281779	-0.328731	-0.369197	-0.353921		0.11196
BS8-A	0.021796	-0.279251	-0.337185	-0.379831	-0.367156		0.09591
BS8-B	0.018615	-0.280213	-0.338863	-0.380751	-0.369281		0.09218
BS7-A	0.011770	-0.293826	-0.356408	-0.393178	-0.375853		0.08827
BS8-C	0.024204	-0.280004	-0.337721	-0.379041	-0.365101		0.10271
BS7-B	0.013159	-0.288743	-0.346790	-0.386094	-0.370890		0.10770
BS8-D	0.021050	-0.277791	-0.337159	-0.380429	-0.366410		0.10414
BS5-A	0.036424	-0.244800	-0.297193	-0.356859	-0.358046		0.10624
BS5-B	0.036072	-0.252662	-0.303041	-0.348706	-0.345641		0.10365
BS4-A	0.033503	-0.274622	-0.323695	-0.359774	-0.347444		0.08711
BS7-C	0.009608	-0.305389	-0.352783	-0.394946	-0.380177	-0.385656	0.08666
BS8-E	0.016226	-0.306453	-0.350447	-0.395255	-0.382348	-0.387713	0.08429
BS8-F	0.016720	-0.298387	-0.346968	-0.392966	-0.380937	-0.386193	0.08716
BS5-C	0.028775	-0.257313	-0.309178	-0.365435	-0.366255		0.10850
BS4-B	0.022972	-0.275849	-0.327340	-0.367484	-0.353577		0.10940
BS5-D	0.029747	-0.258257	-0.317180	-0.357113	-0.354539		0.08308
BS4-C	0.021727	-0.274881	-0.351995	-0.368593	-0.353227		0.11247
BS5-E	0.026097	-0.260294	-0.321079	-0.364067	-0.367336		0.08462
BS5-F	0.030319	-0.267021	-0.323392	-0.355363	-0.350530		0.08016
BS1	0.064277	-0.208181	-0.250076	-0.319433	-0.326568		0.09848

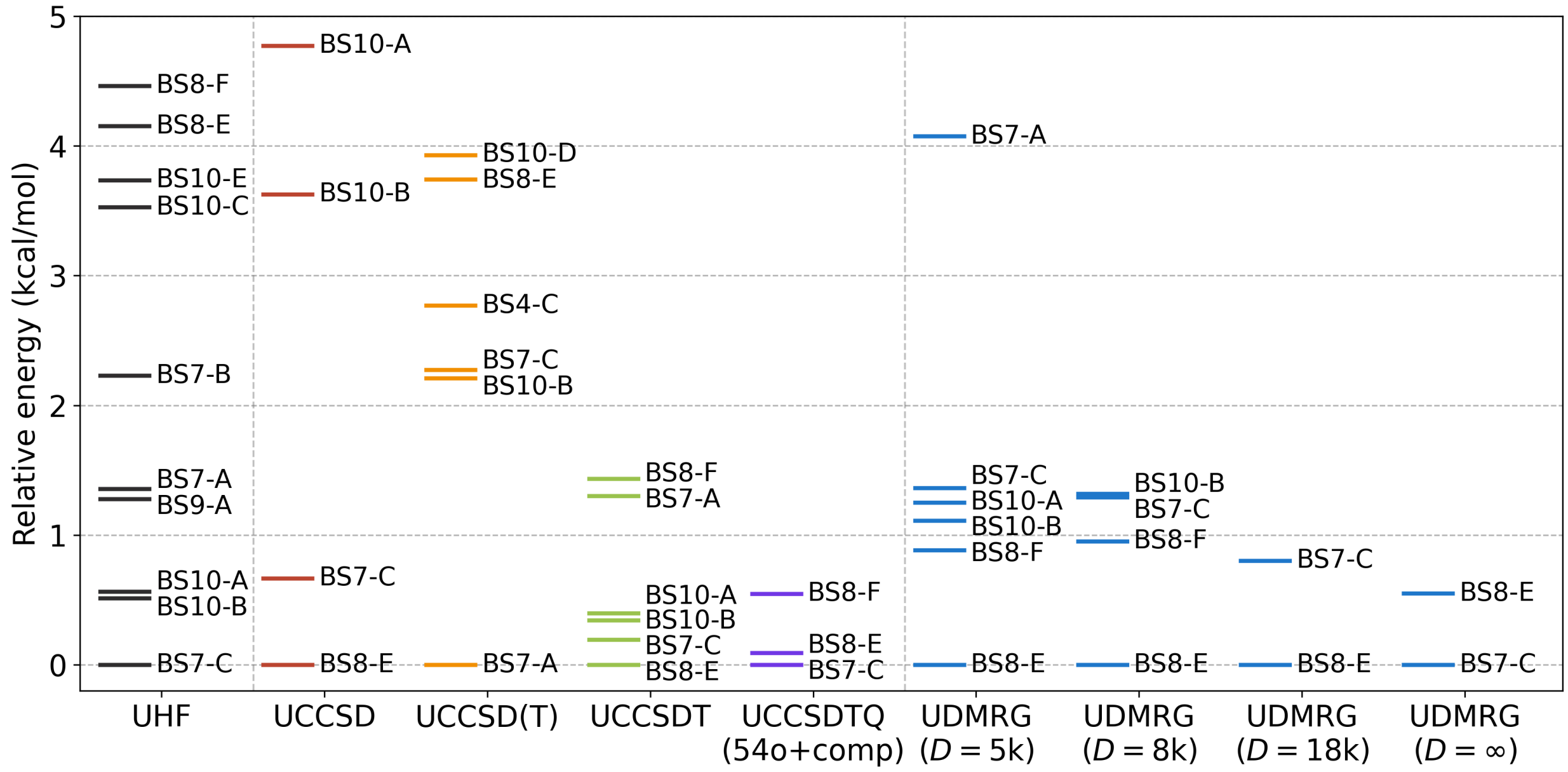

Supplementary Figure 6: **UHF, UCC, and UDMRG relative energies computed for the LLDUC model for low-energy spin isomers.** Note that only 4, 3, and 2 low-energy spin isomers were computed at UDMRG($D = 8000$), UCCSDTQ(54o), and UDMRG($D = 18000$) levels of theory, respectively.

for large MPS bond dimension (for DMRG).

The [2Fe-2S] 20-orbital active space model. We first study the correlation between $\langle S^2 \rangle$ and the energy error for UCC and UDMRG solutions, using the minimal active space model with (20o, 30e) and (20o, 31e) for Fe(III)–Fe(III) and Fe(III)–Fe(II) dimers, respectively. The active space model was defined in Ref. (*22*) and the integral file can be obtained from `https://github.com/zhendongli2008/Active-space-model-for-Iron-Sulfur-Clusters/blob/main/Fe2S2_and_Fe4S4/Fe2S2/fe2s2`. Note that the core energy -4976.26532397 Hartrees for this active space model is not included in the reported total energy. For this model we can obtain the exact energy for the pure spin state using spin-adapted DMRG (SA-DMRG), based on which the energy error for UCC (up to UCCSDTQP) and UDMRG (with different MPS bond dimensions) can be computed. The results are listed in Supplementary Table 5-8 and are plotted in Supplementary Figure 7. We can see that when we increase the UHF/CC excitation order, $\langle S \rangle$ approaches the pure spin S value monotonically. For UDMRG, when the MPS bond dimension D is sufficiently large, $\langle S \rangle$ converges to the pure spin value. From Supplementary Figure 7C and Supplementary Figure 7F we see that for UDMRG the spin contamination quickly decreases once the energy error is below a threshold (10^{-3} Hartrees for the [2Fe-2S] case). Therefore, the energy scale 10^{-3}, which is related to the spin gap of the problem, can be viewed as the error caused by the broken-symmetry scheme for this system, and is removed once the accuracy of the theory

itself is sufficient to distinguish different states at this scale, or in the energy extrapolation procedure (to infinite MPS bond dimension or infinite CC excitation order).

Supplementary Table 5: UHF, UCC, and the exact (estimated using large bond dimension spin-adapted DMRG) energies, $\langle S^2\rangle, 2\langle S\rangle$, and $2S_z$ for a (20o, 30e) active space model of the Fe(III)–Fe(III) dimer.

theory	energy (Ha)	$\langle S^2\rangle$	$2\langle S\rangle$	$2S_z$ (Fe1)	$2S_z$ (Fe2)
UHF	-116.512692	4.893150	3.535703	-4.197729	4.200378
UCCSD	-116.580470	3.601338	2.924965	-3.916799	3.919647
UCCSD(T)	-116.596038	3.104838	2.663243	-3.818287	3.821016
UCCSDT	-116.600883	2.719797	2.446620	-3.763482	3.766248
UCCSDTQ	-116.604181	1.600034	1.720319	-3.418594	3.421149
UCCSDTQP	-116.604756	1.077378	1.304238	-2.931590	2.933808
SA-DMRG[D=12k]	-116.605609	0.000000	0.000000		

Supplementary Table 6: UHF UCC, and the exact (estimated using large bond dimension spin-adapted DMRG) energies, $\langle S^2\rangle, 2\langle S\rangle$, and $2S_z$ for a (20o, 31e) active space model of the Fe(III)–Fe(II) dimer.

theory	energy (Ha)	$\langle S^2\rangle$	$2\langle S\rangle$	$2S_z$ (Fe1)	$2S_z$ (Fe2)
UHF	-116.322099	4.660419	3.431893	-3.773833	4.125092
UCCSD	-116.361322	3.294955	2.765610	-3.603649	3.918227
UCCSD(T)	-116.367095	2.942437	2.573479	-3.537574	3.866800
UCCSDT	-116.372305	2.276939	2.179270	-3.332917	3.733398
UCCSDTQ	-116.373827	1.489036	1.637450	-2.739205	3.252087
UCCSDTQP	-116.373996	1.197270	1.406051	-2.212663	2.777618
SA-DMRG[D=12k]	-116.374322	0.750000	1.000000		

Supplementary Table 7: UDMRG energies, $\langle S^2\rangle, 2\langle S\rangle$, and $2S_z$ at different bond dimensions for a (20o, 30e) active space model of the Fe(III)–Fe(III) dimer.

MPS D	energy (Ha)	$\langle S^2\rangle$	$2\langle S\rangle$	$2S_z$ (Fe1)	$2S_z$ (Fe2)
100	-116.598906	3.595044	2.921757	-3.869613	3.870381
200	-116.601798	2.561228	2.353343	-3.734341	3.734903
400	-116.603444	1.551455	1.684366	-3.409451	3.411074
600	-116.604077	1.075703	1.302783	-3.082867	3.084524
800	-116.604421	0.789053	1.038679	-2.762896	2.764400
1000	-116.604571	0.492156	0.722970	-2.321323	2.322487
1200	-116.604751	0.135284	0.241425	-1.330488	1.331083
1600	-116.605170	0.014958	0.029482	-0.439387	0.439701
2000	-116.605377	0.010609	0.020997	-0.373072	0.373298
2400	-116.605476	0.011585	0.022907	-0.391477	0.391762

The FeMo-co LLDUC model. We further study $\langle S^2\rangle$ for the FeMo-co LLDUC model. We list the results in Supplementary Tables 9 and 10. The correlation between $\langle S\rangle$ and the FeMo-co LLDUC model total energy (computed using UHF, UCCSD, UCCSDT, and UDMRG) is shown in Supplementary Figure 8. We see that the $\langle S\rangle$ computed at UCCSDT and UDMRG ($D = 11000$) is between 5/2 and 3 [these do not correspond to the highest levels of theory used for the energy (FNO-UCCSDTQ and UDMRG ($D = 18000$)]. As $\langle S\rangle$ is converging from above, we conclude

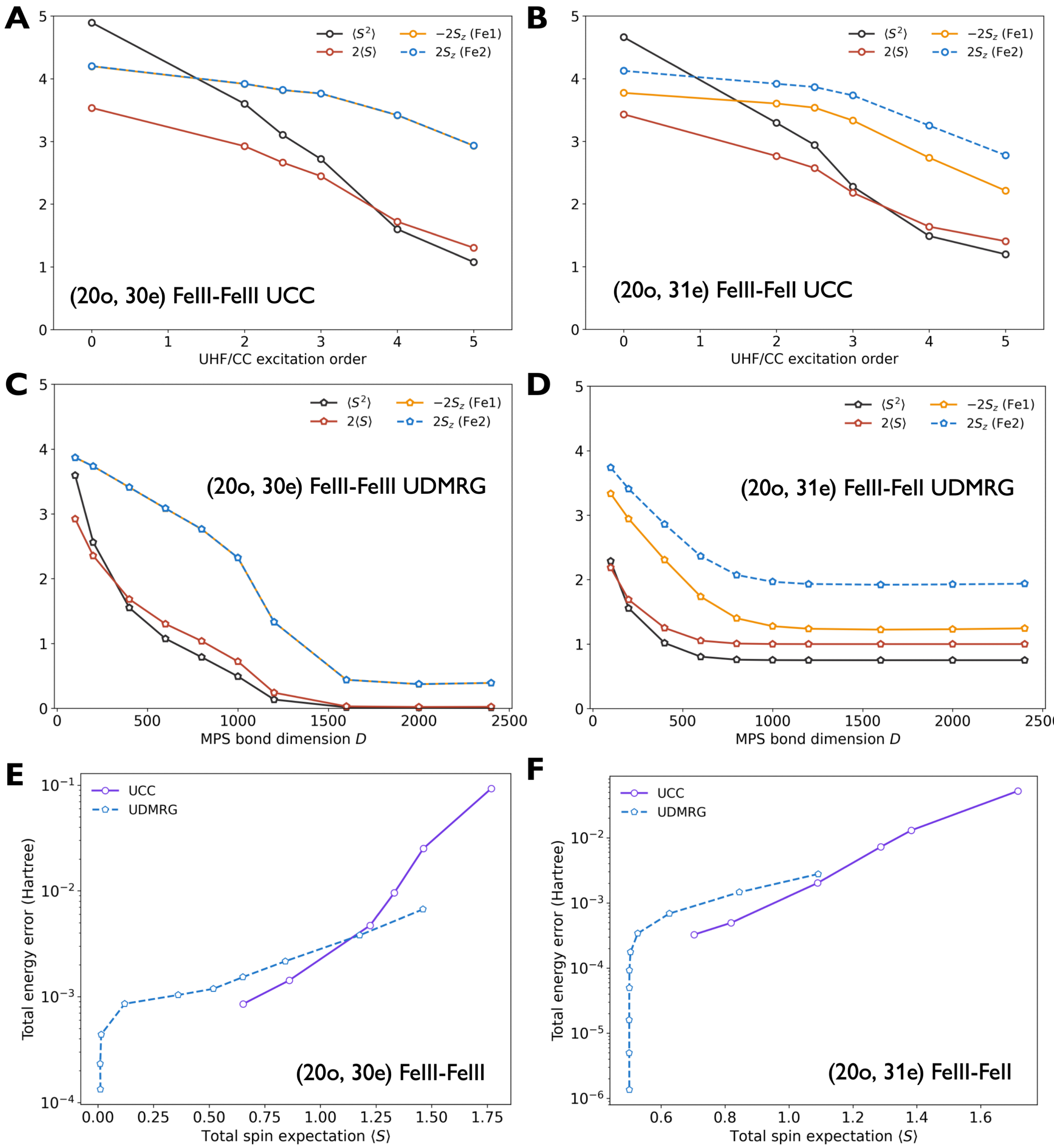

Supplementary Figure 7: **Spin operator expectation values for the [2Fe2S] dimer. A,B** $\langle S^2 \rangle, 2\langle S \rangle$, and $2S_z$ for the (20o, 30e) and (20o, 31e) active space models of the [2Fe2S] dimer as a function of CC excitation order (with UHF = 0, UCCSD(T) = 2.5). **C,D** $\langle S^2 \rangle, 2\langle S \rangle$, and $2S_z$ for the (20o, 30e) and (20o, 31e) active space models of the [2Fe2S] dimer as a function of MPS bond dimension D. **E** Correlation between energy error and $\langle S \rangle$ for the Fe(III)–Fe(III) model. **F** Correlation between energy error and $\langle S \rangle$ for the Fe(III)–Fe(II) model.

Supplementary Table 8: UDMRG energies, $\langle S^2 \rangle, 2\langle S \rangle$, and $2S_z$ at different bond dimensions for a (20o, 31e) active space model of the Fe(III)–Fe(II) dimer.

MPS D	energy (Ha)	$\langle S^2 \rangle$	$2\langle S \rangle$	$2S_z$ (Fe1)	$2S_z$ (Fe2)
100	-116.371550	2.284151	2.183803	-3.332811	3.738070
200	-116.372867	1.557705	1.689018	-2.942814	3.408071
400	-116.373636	1.017338	1.251522	-2.306630	2.858374
600	-116.373982	0.804693	1.053965	-1.736648	2.363313
800	-116.374146	0.758738	1.008719	-1.401038	2.072533
1000	-116.374230	0.752147	1.002146	-1.278340	1.966404
1200	-116.374273	0.751040	1.001040	-1.238854	1.932182
1600	-116.374306	0.750770	1.000770	-1.225353	1.920452
2000	-116.374317	0.750908	1.000908	-1.231956	1.926105
2400	-116.374321	0.751185	1.001184	-1.243982	1.936448

the ground state $S \leq 5/2$, which is consistent with the experimental $S = 3/2$ for this system. However, we do not have a guarantee that the LLDUC model shares the same ground-state S as the true system.

Supplementary Table 9: UHF, UCCSD, and UCCSDT energies (in Hartrees, shifted by -22140.0 Hartrees) and $\langle S^2 \rangle$ of BS7-C, BS8-E, and BS8-F states computed for the LLDUC model.

spin isomer	E_{UHF}	$\langle S^2 \rangle_{\mathrm{UHF}}$	E_{UCCSD}	$\langle S^2 \rangle_{\mathrm{UCCSD}}$	E_{UCCSDT}	$\langle S^2 \rangle_{\mathrm{UCCSDT}}$
BS7-C	0.009608	17.88	-0.305389	11.92	-0.394946	9.31
BS8-E	0.016226	17.90	-0.306453	12.62	-0.395255	8.22
BS8-F	0.016720	17.96	-0.298387	12.56	-0.392966	10.43

Supplementary Table 10: UDMRG energies (in Hartrees, shifted by -22140.0 Hartrees) and $\langle S^2 \rangle$ of BS8-E computed for the LLDUC model.

MPS D	E_{UDMRG}	$\langle S^2 \rangle_{\mathrm{UDMRG}}$	MPS D	E_{UDMRG}	$\langle S^2 \rangle_{\mathrm{UDMRG}}$
5000	-0.382131	12.27	8000	-0.388369	11.60
6000	-0.384697	12.02	9000	-0.389741	11.42
7000	-0.386720	11.80	11000	-0.391926	11.20

3.1.5 Frozen natural orbital UCC and UDMRG

The large difference between the UCCSD(T) and UCCSDT energies indicates that the full quadruple (Q) contribution can be significant for the LLDUC model. Since UCCSDTQ is too expensive to compute (with our resources) for the complete 76-orbital LLDUC model, we perform UCCSDTQ in a series of smaller active subspaces defined using frozen CCSD natural orbitals (FNO, with spin symmetry breaking). In the FNO model, we keep all 18 and 21 (for α and β spin, respectively) virtual orbitals in all FNO subspaces. For the occupied orbitals, we first compute the broken-symmetry UCCSD natural orbitals (for a given spin isomer) by separately diagonalizing the occupied-occupied block of the UCCSD one-particle density matrix for α and β spins, and then freeze $22, 26, 30, \cdots, 46$ α and β occupied natural orbitals with the highest natural orbital occupation. The "FNO occupation cutoff" is then defined as the sum

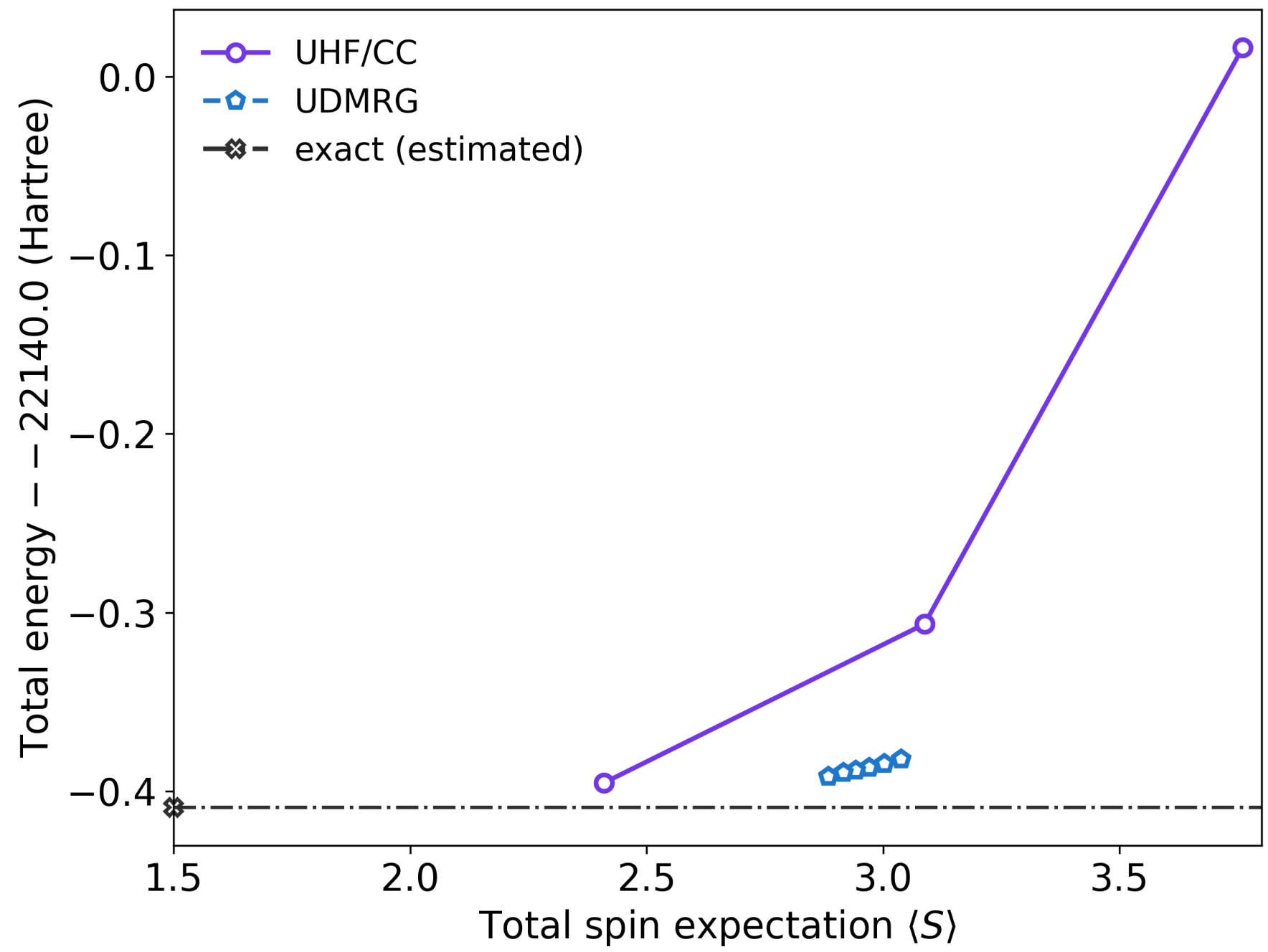

Supplementary Figure 8: **Correlation between shifted total energy and $\langle S \rangle$ of BS8-E computed for the LLDUC model.**

of the α and β spin natural orbital cutoffs (see Supplementary Table 11).

We also perform UDMRG (up to MPS bond dimension 8000) within each of the FNO subspaces, and use the extrapolated UDMRG energies to estimate the error in UCCSDTQ. The results are listed in Supplementary Table 12. In the smaller subspaces (e.g. ≤ 38o for BS7-C, BS8-F, and ≤ 46o for BS8-E), the extrapolated UDMRG number lies below the UCCSDTQ number by at most 0.99 mHa. In the larger subspaces, the UDMRG number lies above the UCCSDTQ number by at most 0.95 mHa. For the smallest FNO space with (30o, 21e), we also computed UCCSDTQP energies and the results are listed in Supplementary Table 13. The largest energy difference between the extrapolated UDMRG and UCCSDTQP energies in the (30o, 21e) is 0.38 mHa (with the UDMRG number lying below the UCCSDTQP energy).

There are two interpretations of the data in the FNO space. The first is that the UCCSDTQ energies become non-variational in the larger active spaces. Supporting this is the fact that the UCCSDTQP energy for (30o, 21e) actually lies slightly (0.009 mHa) above the UCCSDTQ energy for BS8-E, suggesting non-uniform convergence with excitation level, although the UDMRG estimate for the exact number still supports the variationality of the UCCSDTQ energy. The second is that the extrapolated DMRG energies lie above the true energy in the larger active spaces. Since the

extrapolation distance for DMRG increases in the larger active spaces, the accuracy of the extrapolation is clearly lower. Without further evidence, it is not possible to pick between these scenarios with high confidence, other than to say that in all cases the data supports the UCCSDTQ energy being either variational, or very close (within 1 kcal/mol) of the 'exact' estimate. In the iron cubane (SM Sec. 4), where very accurate variational DMRG numbers can be produced, we show that the UCCSDTQ energy is indeed variational and that the extrapolated UDMRG number can sometimes be above this variational estimate.

As shown in Fig. 3B, we see a large curvature in the FNO-UCCSDTQ composite corrected UCCSDT energy versus FNO occupation cutoff curve, which prevents the composite energy from being simply extrapolated using linear regression. This may be partially explained by the non-linear change in $\langle S^2 \rangle$ when considering different FNO subspaces (see Supplementary Table 14). In fact, from Supplementary Figure 9 we see a similar non-linear trend for the $\langle S \rangle$ computed for UCCSD and UCCSDT in different FNO subspaces. As each FNO active space series is defined for a specific spin isomer (to compute the UCCSD natural orbitals), the energy of states with other spin couplings will be significantly increased in the small FNO spaces, and the near-degeneracy among the spin isomers will be recovered only when the FNO space is very close to the full 76-orbital space.

Supplementary Table 11: Natural orbital cutoff in FNO subspaces for BS7-C, BS8-E, and BS8-F states for the LLDUC model.

spin isomer	(30o, 21e)	(34o, 29e)	(38o, 37e)	(42o, 45e)	(46o, 53e)	(50o, 61e)	(54o, 69e)
BS7-C	1.937625	1.964417	1.975624	1.984529	1.989548	1.992606	1.994763
BS8-E	1.931639	1.965347	1.976265	1.987435	1.990742	1.993289	1.995066
BS8-F	1.934736	1.968874	1.977565	1.985572	1.991067	1.993575	1.995543

Supplementary Table 12: UHF, UCC and UDMRG energies in the FNO subspaces for BS7-C, BS8-E, and BS8-F states for the LLDUC model. Energies are in Hartrees shifted by -22140.0 Hartrees. Extrapolated UDMRG energies are computed using the $\exp[-\kappa(\log D)^2]$ fitting based on data with MPS bond dimensions $D = 7500, 7000, \cdots, 5000$.

spin isomer	FNO subspace	E_{UHF}	E_{UCCSD}	E_{UCCSDT}	E_{UCCSDTQ}	$E^{D=8000}_{\mathrm{UDMRG}}$	$E^{\mathrm{extrap}}_{\mathrm{UDMRG}}$	$E_{\mathrm{UCCSDTQ}} - E^{\mathrm{extrap}}_{\mathrm{UDMRG}}$
BS7-C	(30o, 21e)	0.013987	-0.102385	-0.123010	-0.126991	-0.127483	-0.127539	+0.000548
	(34o, 29e)	0.013987	-0.154096	-0.188616	-0.193582	-0.193110	-0.193973	+0.000391
	(38o, 37e)	0.013987	-0.190847	-0.235962	-0.241705	-0.240057	-0.241741	+0.000036
	(42o, 45e)	0.013987	-0.215455	-0.268514	-0.274840	-0.270758	-0.274277	-0.000563
	(46o, 53e)	0.013987	-0.237776	-0.298481	-0.305408	-0.300172	-0.304977	-0.000431
	(50o, 61e)	0.013987	-0.255100	-0.321765	-0.329905	-0.319909	-0.328953	-0.000952
	(54o, 69e)	0.013987	-0.269585	-0.342146	-0.351627			
BS8-E	(30o, 21e)	0.076451	-0.098580	-0.136016	-0.136728	-0.136941	-0.137102	+0.000374
	(34o, 29e)	0.076451	-0.145703	-0.196855	-0.200313	-0.200097	-0.201260	+0.000947
	(38o, 37e)	0.076451	-0.181494	-0.243404	-0.248464	-0.246454	-0.249325	+0.000861
	(42o, 45e)	0.076451	-0.206176	-0.276008	-0.281959	-0.278102	-0.282951	+0.000992
	(46o, 53e)	0.076451	-0.226300	-0.303998	-0.310556	-0.302324	-0.311081	+0.000524
	(50o, 61e)	0.076451	-0.242257	-0.327246	-0.335307	-0.323274	-0.335004	-0.000303
	(54o, 69e)	0.076451	-0.255650	-0.347246	-0.356269			
BS8-F	(30o, 21e)	0.018614	-0.112553	-0.141566	-0.146057	-0.146808	-0.146984	+0.000927
	(34o, 29e)	0.018614	-0.158841	-0.203066	-0.209615	-0.209593	-0.210390	+0.000775
	(38o, 37e)	0.018614	-0.190547	-0.245325	-0.252449	-0.251102	-0.252766	+0.000317
	(42o, 45e)	0.018614	-0.215290	-0.279408	-0.287242	-0.282822	-0.287116	-0.000125
	(46o, 53e)	0.018614	-0.235811	-0.306788	-0.315083	-0.306934	-0.315018	-0.000064
	(50o, 61e)	0.018614	-0.252355	-0.329961	-0.339335	-0.328617	-0.338639	-0.000696
	(54o, 69e)	0.018614	-0.265288	-0.348697	-0.359283			

Supplementary Table 13: UCCSDTQP and UDMRG energies in the FNO subspace (30o, 21e) for BS7-C, BS8-E, and BS8-F states for the LLDUC model. Energies are in Hartrees shifted by -22140.0 Hartrees. Extrapolated UDMRG energies are computed using the $\exp[-\kappa(\log D)^2]$ fitting based on data with MPS bond dimensions $D = 7500, 7000, \cdots, 5000$.

spin isomer	FNO subspace	E_{UCCSDTQP}	$E^{D=8000}_{\mathrm{UDMRG}}$	$E^{\mathrm{extrap}}_{\mathrm{UDMRG}}$	$E_{\mathrm{UCCSDTQP}} - E^{\mathrm{extrap}}_{\mathrm{UDMRG}}$
BS7-C	(30o, 21e)	-0.127496	-0.127483	-0.127539	+0.000043
BS8-E	(30o, 21e)	-0.136719	-0.136941	-0.137102	+0.000383
BS8-F	(30o, 21e)	-0.146846	-0.146808	-0.146984	+0.000138

Supplementary Table 14: UCC and UDMRG $\langle S^2 \rangle$ in the FNO subspaces for BS7-C, BS8-E, and BS8-F states for the LLDUC model.

spin isomer	FNO subspace	$\langle S^2\rangle_{\mathrm{UCCSD}}$	$\langle S^2\rangle_{\mathrm{UCCSDT}}$	$\langle S^2\rangle_{\mathrm{UCCSDTQ}}$	$\langle S^2\rangle^{D=5000}_{\mathrm{UDMRG}}$	$\langle S^2\rangle^{D=8000}_{\mathrm{UDMRG}}$
BS7-C	(30o, 21e)	16.70	16.66	16.66	16.66	16.66
	(34o, 29e)	16.52	16.46		16.49	16.48
	(38o, 37e)	16.15	16.01		16.07	16.05
	(42o, 45e)	15.79	15.59		15.71	15.67
	(46o, 53e)	15.30	14.95		15.15	15.08
	(50o, 61e)	14.80	14.27		14.74	14.62
	(54o, 69e)	14.14	13.31			
BS8-E	(30o, 21e)	16.77	16.68	16.52	16.52	16.52
	(34o, 29e)	16.53	16.41		16.34	16.33
	(38o, 37e)	16.16	16.02		16.12	16.09
	(42o, 45e)	15.87	15.65		15.86	15.82
	(46o, 53e)	15.46	15.13		15.61	15.53
	(50o, 61e)	14.89	14.30		15.20	15.08
	(54o, 69e)	14.13	12.94			
BS8-F	(30o, 21e)	16.74	16.63	16.66	16.67	16.67
	(34o, 29e)	16.48	16.35		16.44	16.43
	(38o, 37e)	16.11	15.96		16.09	16.08
	(42o, 45e)	15.76	15.53		15.77	15.72
	(46o, 53e)	15.42	15.10		15.49	15.40
	(50o, 61e)	15.08	14.57		15.11	14.98
	(54o, 69e)	14.73	14.03			

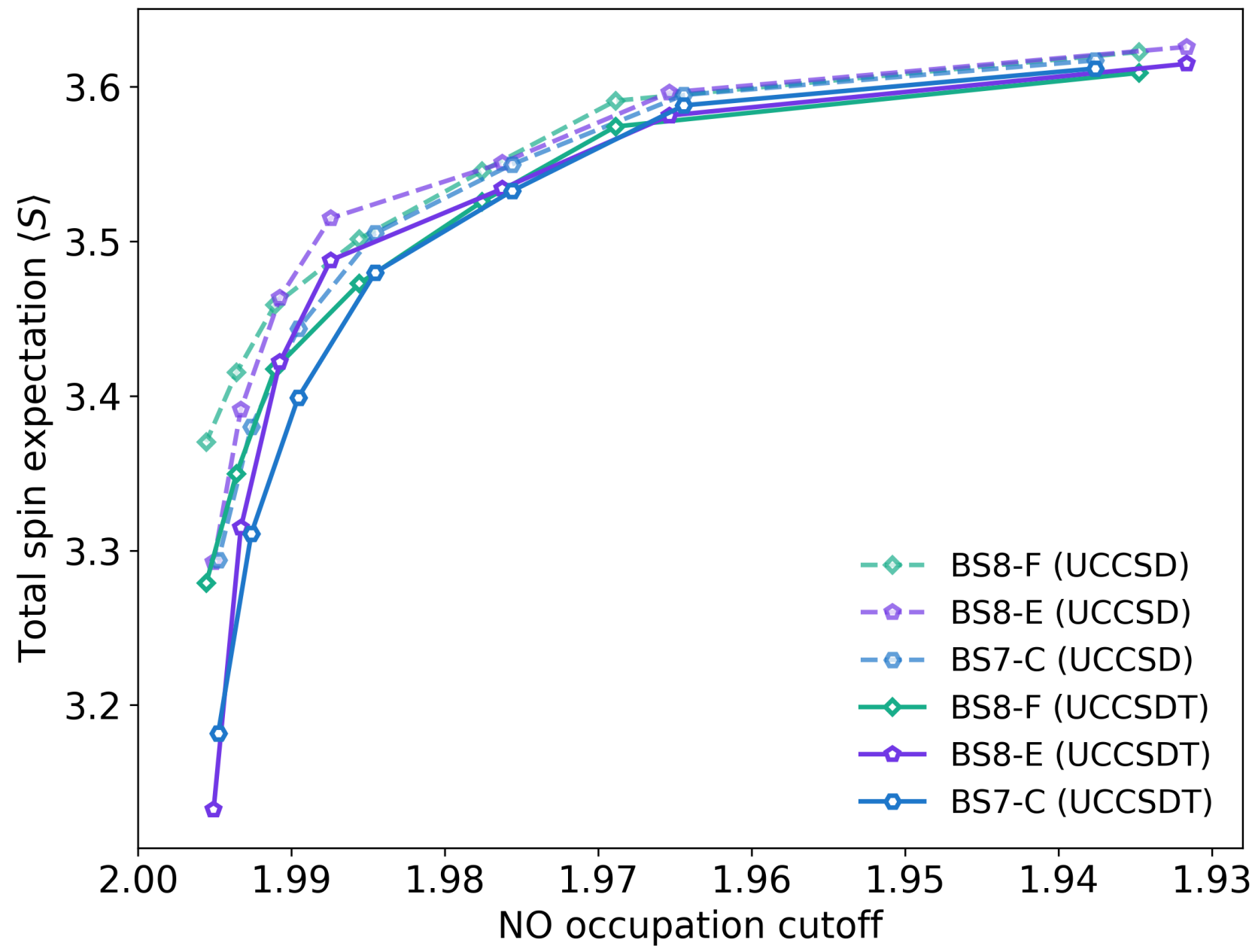

Supplementary Figure 9: **UHF/CCSD and UHF/CCSDT $\langle S \rangle$ in the FNO subspaces for BS7-C, BS8-E, and BS8-F states for the LLDUC model.**

FNO-UCCSDTQ composite correction. The FNO-UCCSDTQ composite energy is computed as

$$E_{\text{compsosite}} = E_{\text{UCCSDT}} + E_{\text{FNO-UCCSDTQ}} - E_{\text{FNO-UCCSDT}} \tag{S5}$$

We list the FNO-UCCSD, FNO-UCCSDT, FNO-UCCSDTQ, full space UCCSD, UCCSDT, and the composite energies for BS7-C, BS8-E, and BS8-F in Supplementary Table 15. Extrapolation was done using linear fitting for the 46o, 50o, and 54o energies with respect to UCCSD natural orbital occupation cutoff. Note that the UCCSD energies listed in Supplementary Table 15 were computed using the same reference as that of UCCSDT.

3.1.6 Overlap with dominant determinants

Given that the bond dimension $D = 18000$ MPS obtained from UDMRG is a good approximate ground state of the LLDUC model, we can compute an estimate of the overlap of the dominant broken symmetry determinant with this state. Using an efficient sweep-like algorithm (*55, 92*), we can extract all determinants (defined in the split-localized broken-symmetry CCSD natural orbital basis) with the absolute value of the overlap larger than a given value. The overlap with dominant determinants (with absolute value larger than 0.05) are listed in Supplementary Table 16. We find that the overlaps with the dominant determinant are 0.4738 and 0.4468 for BS7-C and BS8-E, respectively. The next largest overlap is below 0.1 in both states.

Supplementary Table 15: UCCSD, UCCSDT and UCCSDTQ energies in the FNO subspaces, and full space UCCSD, UCCSDT and composite energies for BS7-C, BS8-E, and BS8-F states for the LLDUC model. Energies are in Hartrees shifted by -22140.0 Hartrees. Extrapolation was done using linear fitting for 46o, 50o, and 54o energies with respect to UCCSD natural orbital occupation cutoff. The listed UCCSD energies use the same reference as that of UCCSDT, namely the reference for the best full space UCCSDT energy.

spin isomer	FNO subspace	E_{UCCSD}	E_{UCCSDT}	E_{UCCSDTQ}	$E_{\mathrm{composite}}$
BS7-C	(30o, 21e)	-0.102385	-0.123010	-0.126991	-0.398928
	(34o, 29e)	-0.154096	-0.188616	-0.193582	-0.399912
	(38o, 37e)	-0.190847	-0.235962	-0.241705	-0.400689
	(42o, 45e)	-0.215455	-0.268514	-0.274840	-0.401272
	(46o, 53e)	-0.237776	-0.298481	-0.305408	-0.401873
	(50o, 61e)	-0.255100	-0.321765	-0.329905	-0.403087
	(54o, 69e)	-0.269585	-0.342146	-0.351627	-0.404427
	(76o, 113e)	-0.304318	-0.394946	**extrapolated**	**-0.406849**
BS8-E	(30o, 21e)	-0.098580	-0.136016	-0.136728	-0.395967
	(34o, 29e)	-0.145703	-0.196855	-0.200313	-0.398713
	(38o, 37e)	-0.181494	-0.243404	-0.248464	-0.400315
	(42o, 45e)	-0.206176	-0.276008	-0.281959	-0.401206
	(46o, 53e)	-0.226300	-0.303998	-0.310556	-0.401813
	(50o, 61e)	-0.242257	-0.327246	-0.335307	-0.403316
	(54o, 69e)	-0.255650	-0.347246	-0.356269	-0.404278
	(76o, 113e)	-0.284755	-0.395255	**extrapolated**	**-0.407117**
BS8-F	(30o, 21e)	-0.112553	-0.141566	-0.146057	-0.397456
	(34o, 29e)	-0.158841	-0.203066	-0.209615	-0.399515
	(38o, 37e)	-0.190547	-0.245325	-0.252449	-0.400091
	(42o, 45e)	-0.215290	-0.279408	-0.287242	-0.400800
	(46o, 53e)	-0.235811	-0.306788	-0.315083	-0.401261
	(50o, 61e)	-0.252355	-0.329961	-0.339335	-0.402340
	(54o, 69e)	-0.265288	-0.348697	-0.359283	-0.403552
	(76o, 113e)	-0.292974	-0.392966	**extrapolated**	**-0.405741**

Supplementary Table 16: Overlap between the bond dimension $D = 18000$ MPS obtained from UDMRG and the dominant determinants (with absolute value larger than 0.05).

spin isomer	determinant overlap				
BS7-C	-0.473757	-0.081447	-0.078313	-0.058582	0.053784
BS8-E	-0.446836	0.099686	-0.075533	0.074390	-0.063381
	-0.061440	0.056716	-0.055592	0.054155	-0.052423

3.1.7 Computational cost and scaling

In this section, we summarize the actual scaling and computational cost of the UCC and UDMRG methods used in this work. Performance analysis can help us establish an estimate of the classical simulation time to reach chemical accuracy for the LLDUC model. Timings are measured on AMD EPYC 9474F CPUs with 96 CPU cores and 1.5TB memory per node, supported by the Scientific Computing Core at the Flatiron Institute.

UDMRG. We perform UDMRG for the BS7-C and BS8-E states using a normal forward DMRG schedule up to a large MPS bond dimension ($D = 18000$). After that, to collect data necessary for extrapolation, we additionally perform the reverse schedule with MPS bond dimensions $D = 16000, 15000, \cdots$, and 5000. To make sure that the energy at each of the bond dimensions is fully converged, we do four sweeps for each of the reverse schedule bond dimensions. The computational cost and timings are listed in Supplementary Table 17. Note that forward schedule sweeps are in general more expensive than the reverse schedule sweeps, because in the forward schedule we need to apply perturbative noise to reduce the chance of getting stuck in local minima when increasing the bond dimension. The scaling of the UDMRG computational cost for BS8-E is shown in Supplementary Figure 10A.

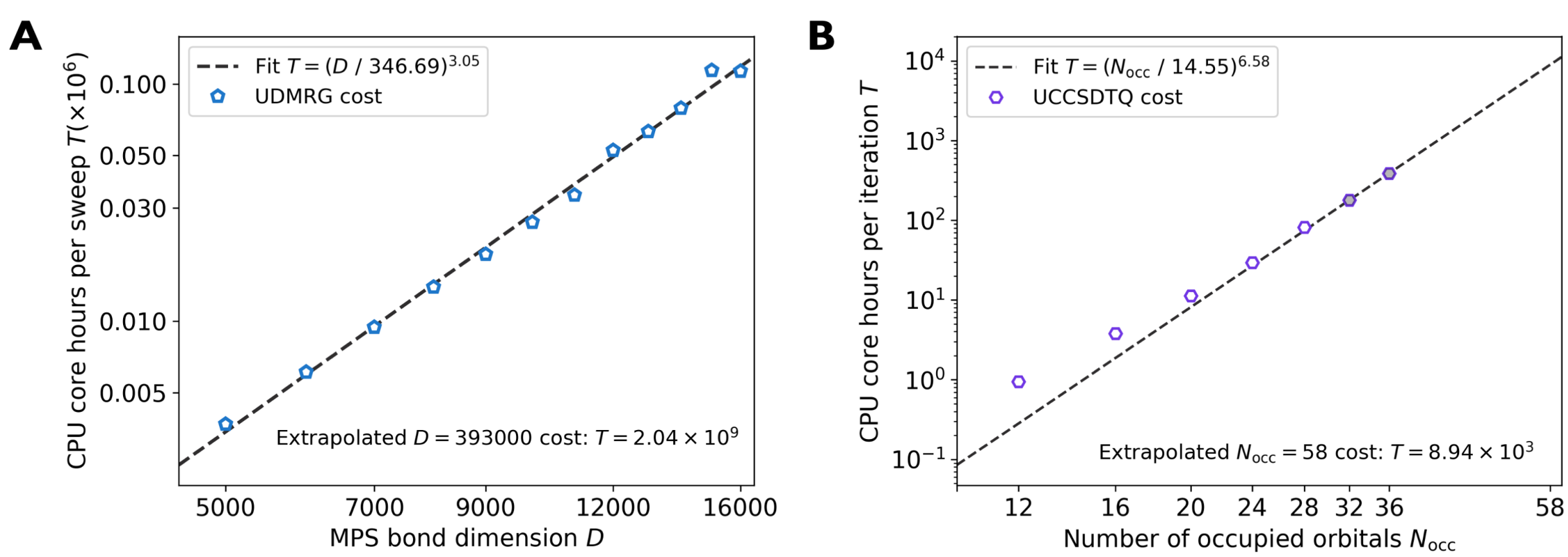

Supplementary Figure 10: **Computational cost and scaling for UDMRG and UCC. A** Computational cost and scaling for UDMRG extrapolation, in CPU core hours per sweep, for BS8-E. **B** Computational cost and scaling for UCCSDTQ in the active subspace, in CPU core hours per iteration, for BS8-E. Extrapolation is done using only the data from the 32 and 36 occupied orbital subspaces, as the cost for smaller subspaces has significant overheads.

UCC. We list the FNO-UCCSDTQ computational cost and timings in Supplementary Table 18. Note that the memory cost is the main bottleneck in these calculations, because currently our UCC code does not support distributed storage of amplitudes. We use index-permutation symmetry to reduce the memory cost of amplitudes, which introduces a non-negligible amount of computational overhead for packing and unpacking in the contraction step. Therefore,

Supplementary Table 17: Computational cost of UDMRG for the LLDUC model.

spin isomer	schedule type	D	N_{nodes}	N_{sweeps}	FLOPs/sweep	wall time/sweep	CPU hours/sweep
BS7-C	forward	18000	16	2	4.01×10^{18}	160.2 hours	246091
	reverse	16000	16	4	2.78×10^{18}	72.7 hours	111639
	reverse	15000	16	4	2.33×10^{18}	62.1 hours	95411
	reverse	14000	16	4	1.91×10^{18}	57.4 hours	88202
	reverse	13000	16	4	1.54×10^{18}	39.9 hours	61298
	reverse	12000	16	4	1.22×10^{18}	31.5 hours	48397
	reverse	11000	16	4	9.49×10^{17}	30.4 hours	46642
	reverse	10000	16	4	7.22×10^{17}	18.7 hours	28732
	reverse	9000	16	4	5.34×10^{17}	13.7 hours	21052
	reverse	8000	16	4	3.78×10^{17}	9.72 hours	14925
	reverse	7000	16	4	2.58×10^{17}	7.76 hours	11917
	reverse	6000	16	4	1.64×10^{17}	5.07 hours	7787
	reverse	5000	16	4	9.86×10^{16}	3.14 hours	4825
						**total CPU hours:**	**2655489**
BS8-E	forward	18000	24	2	5.00×10^{18}	137.8 hours	317440
	reverse	16000	16	4	2.85×10^{18}	73.6 hours	113066
	reverse	15000	16	4	2.38×10^{18}	74.1 hours	113812
	reverse	14000	16	4	1.95×10^{18}	51.4 hours	79026
	reverse	13000	16	4	1.57×10^{18}	41.0 hours	62985
	reverse	12000	16	4	1.25×10^{18}	34.1 hours	52374
	reverse	11000	12	4	8.37×10^{17}	29.5 hours	33928
	reverse	10000	12	4	6.40×10^{17}	22.7 hours	26117
	reverse	9000	12	4	4.77×10^{17}	16.6 hours	19116
	reverse	8000	12	4	3.39×10^{17}	12.1 hours	13918
	reverse	7000	12	4	2.31×10^{17}	8.17 hours	9413
	reverse	6000	12	4	1.49×10^{17}	5.30 hours	6102
	reverse	5000	12	4	8.82×10^{16}	3.19 hours	3671
						**total CPU hours:**	**2768994**
**estimated**		393000			4.54×10^{22}		2.04×10^{9}

for estimating FLOPs/iter we consider the situation where there is no usage of amplitudes with index-permutation symmetry. The scaling of the FNO-UCCSDTQ computational cost for BS8-E is shown in Supplementary Figure 10B.

Supplementary Table 18: Computational cost of FNO-UCCSDTQ per iteration for the LLDUC model. $N_{\rm iter}$ is the number of iterations to achieve energy convergence of 10^{-8} Hartrees. FLOPs/iter is the estimated number without the consideration of index-permutation symmetry (the actual computation was done with index-permutation symmetry to reduce memory costs).

spin isomer	FNO subspace	$N_{\rm nodes}$	$N_{\rm iter}$	FLOPs/iter	wall time/iter	CPU hours/iter
BS7-C	(30o, 21e)	1	46	5.83×10^{13}	0.02 hours	0.9
	(34o, 29e)	1	55	3.07×10^{14}	0.08 hours	3.8
	(38o, 37e)	1	71	1.10×10^{15}	0.24 hours	11.3
	(42o, 45e)	1	73	2.84×10^{15}	0.62 hours	29.7
	(46o, 53e)	1	77	6.10×10^{15}	0.83 hours	79.9
	(50o, 61e)	1	75	1.19×10^{16}	2.50 hours	239.6
	(54o, 69e)	1	80	2.14×10^{16}	3.96 hours	380.5
BS8-E	(30o, 21e)	1	72	5.83×10^{13}	0.02 hours	0.9
	(34o, 29e)	1	90	3.07×10^{14}	0.08 hours	3.8
	(38o, 37e)	1	90	1.10×10^{15}	0.23 hours	11.2
	(42o, 45e)	1	109	2.84×10^{15}	0.61 hours	29.4
	(46o, 53e)	1	103	6.10×10^{15}	0.85 hours	82.0
	(50o, 61e)	1	93	1.19×10^{16}	1.86 hours	178.8
	(54o, 69e)	1	98	2.14×10^{16}	4.04 hours	388.0
BS8-F	(30o, 21e)	1	61	5.83×10^{13}	0.02 hours	0.9
	(34o, 29e)	1	77	3.07×10^{14}	0.08 hours	3.8
	(38o, 37e)	1	70	1.10×10^{15}	0.23 hours	11.3
	(42o, 45e)	1	81	2.84×10^{15}	0.62 hours	29.8
	(46o, 53e)	1	97	6.10×10^{15}	0.83 hours	79.9
	(50o, 61e)	1	113	1.19×10^{16}	1.64 hours	157.0
	(54o, 69e)	1	99	2.14×10^{16}	4.38 hours	420.9
**estimated**	(76o, 113e)			2.32×10^{17}		8937

3.1.8 UDMRG energy extrapolation

We list the UDMRG energies and discarded weights in Supplementary Table 19. The $\exp[-\kappa(\log D)^2]$ fitting is shown in Supplementary Figure 11. We also performed the more conventional DMRG extrapolation using discarded weights (using three data points with the largest bond dimensions), shown in Supplementary Figure 12. We did not see an overall simple trend in the discarded weight extrapolation, which is possibly caused by the usage of spin-unrestricted orbitals in UDMRG.

We estimate the required bond dimension to reach 1 kcal/mol error using the $\exp[-\kappa(\log D)^2]$ fitting of the BS8-E data listed in Supplementary Table 19. The energy at $D = 393000$ is predicted to be -22140.408505 Hartrees, which is 0.001592 Hartrees or 1.00 kcal/mol above the extrapolated BS8-E energy using the $\exp[-\kappa(\log D)^2]$ fit (-22140.410097 Hartrees).

Supplementary Table 19: UDMRG energies and discarded weights for BS7-C and BS8-E of LLDUC model.

spin isomer	schedule type	D	energy (Hartree)	max discarded weight
BS7-C	forward	18000	-22140.394853	7.10×10^{-5}
	reverse	16000	-22140.394216	8.52×10^{-5}
	reverse	15000	-22140.393634	8.88×10^{-5}
	reverse	14000	-22140.392959	9.15×10^{-5}
	reverse	13000	-22140.392205	9.38×10^{-5}
	reverse	12000	-22140.391360	9.59×10^{-5}
	reverse	11000	-22140.390407	9.84×10^{-5}
	reverse	10000	-22140.389322	1.01×10^{-4}
	reverse	9000	-22140.388070	1.04×10^{-4}
	reverse	8000	-22140.386616	1.07×10^{-4}
	reverse	7000	-22140.384879	1.11×10^{-4}
	reverse	6000	-22140.382754	1.20×10^{-4}
	reverse	5000	-22140.380060	1.40×10^{-4}
	$\exp[-\kappa(\log D)^2]$ fit	∞	-22140.410973	
BS8-E	forward	18000	-22140.396133	4.10×10^{-5}
	reverse	16000	-22140.395472	5.22×10^{-5}
	reverse	15000	-22140.394930	5.50×10^{-5}
	reverse	14000	-22140.394302	5.70×10^{-5}
	reverse	13000	-22140.393599	5.88×10^{-5}
	reverse	12000	-22140.392814	6.05×10^{-5}
	reverse	11000	-22140.391926	6.20×10^{-5}
	reverse	10000	-22140.390912	6.40×10^{-5}
	reverse	9000	-22140.389741	6.70×10^{-5}
	reverse	8000	-22140.388369	7.42×10^{-5}
	reverse	7000	-22140.386720	8.28×10^{-5}
	reverse	6000	-22140.384697	9.36×10^{-5}
	reverse	5000	-22140.382131	1.08×10^{-4}
	$\exp[-\kappa(\log D)^2]$ fit	∞	-22140.410097	

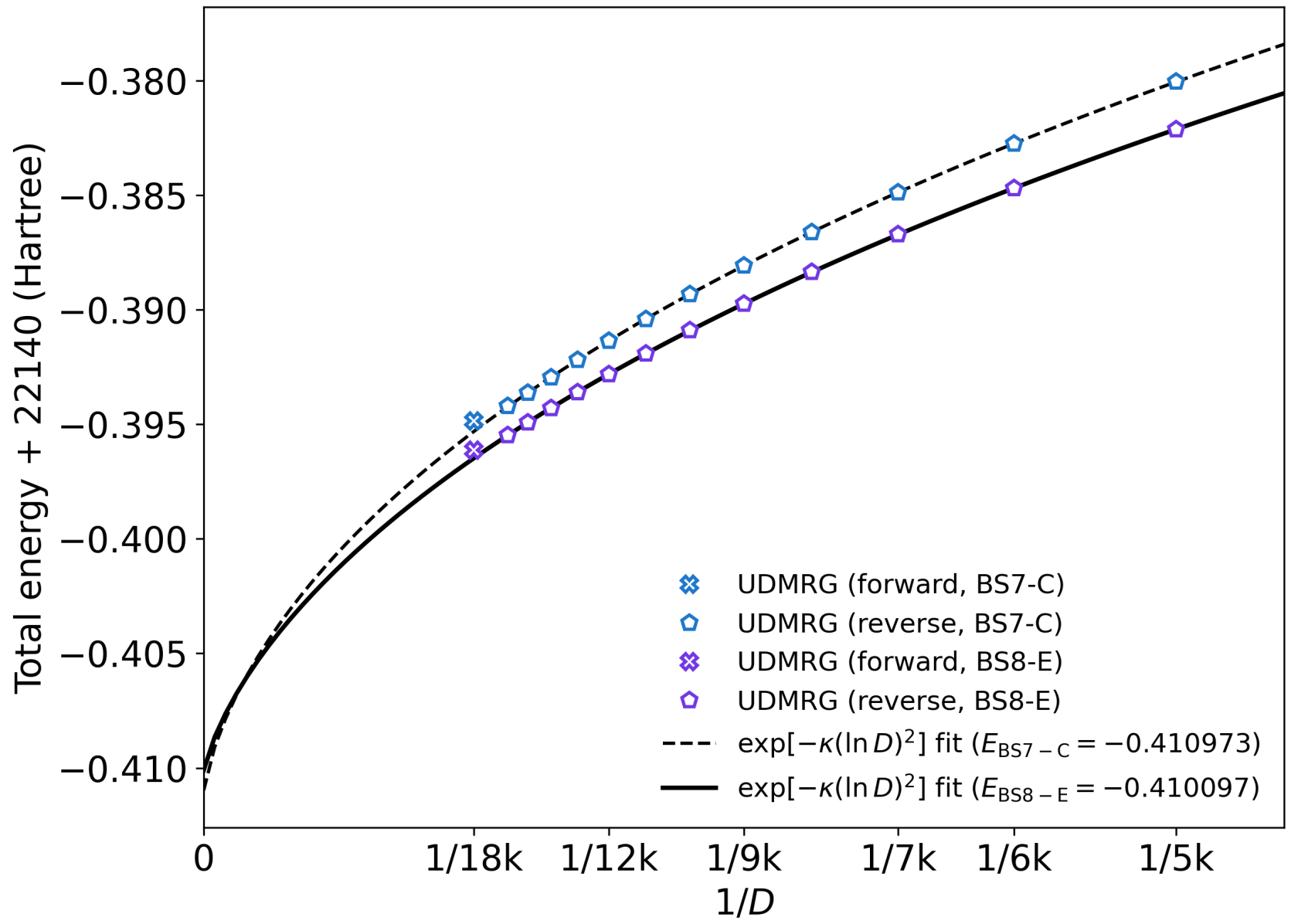

Supplementary Figure 11: **The $\exp[-\kappa(\log D)^2]$ fit for BS7-C and BS8-E states for the LLDUC model, using UDMRG energy data obtained from the reverse schedule.**

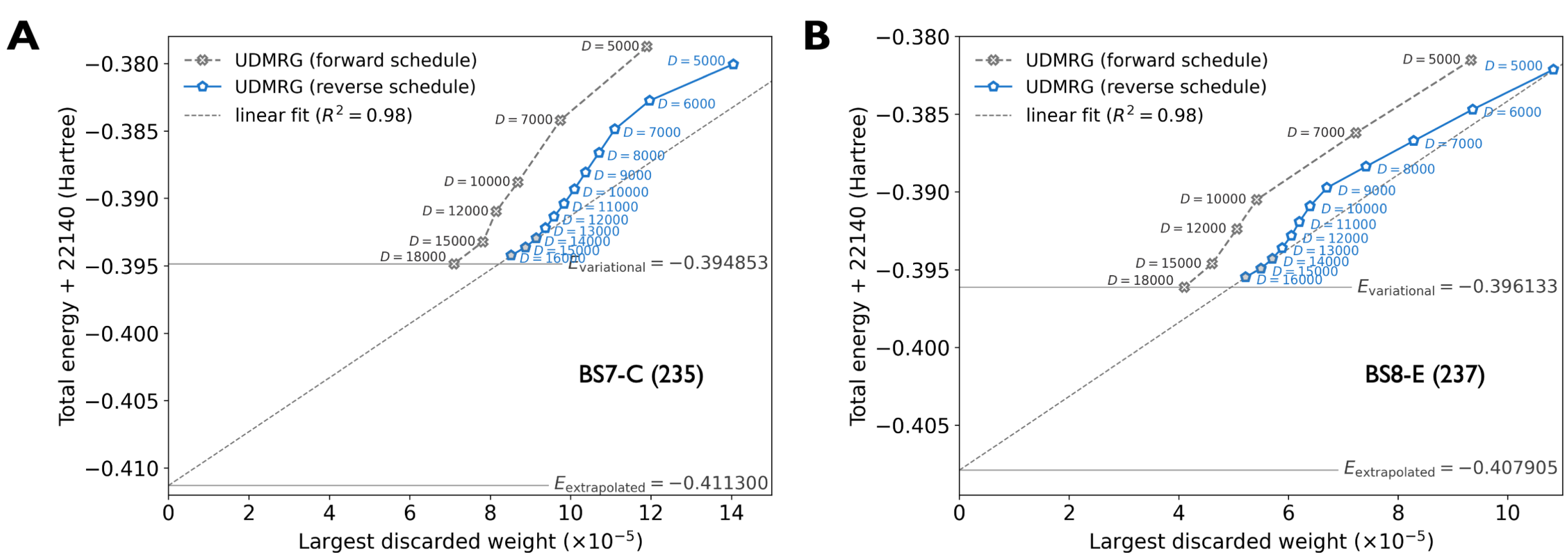

Supplementary Figure 12: **The low confidence discarded weight extrapolation for the (A) BS7-C and (B) BS8-E states for the LLDUC model, using UDMRG energy and discarded weights obtained from the reverse schedule.** We perform linear extrapolation using the three data points with bond dimension 14000, 15000, and 16000. The energy and discarded weights obtained from the forward schedule are shown in dashed lines.

3.1.9 Composite energy estimation

We estimate the final UCC energy for BS7-C, BS8-E and BS8-F by adding a composite post-Q correction. To estimate the contribution of post quadruples connected correlations, we use an Fe dimer reference system (FeIII-FeII, see Sec. 3.1.4) for which we have computed the D, T, Q and post-Q correlation increments, against which we can compare the corresponding D, T, and Q correlation increments for FeMo-co. If FeMo-co can be viewed as an extension of the Fe dimer (i.e. multiple connected copies with the same qualitative correlations), then we can expect a constant ratio of the increments between the systems. (Of course, whether this type of relationship can be found or holds will depend on the system, thus this scaling relation is an empirical one).

In Supplementary Table 20 we show the correlation increments and ratios. For LLDUC model, we use the UHF, UCCSD, and UCCSDT energies with their respective best reference to minimize the fluctuation and reference dependence of the increment ratio at different correlation levels. We see that the ratio of the correlation increments is indeed roughly constant, suggesting that the chosen Fe dimer is a good model system for the correlations in FeMo-co. We estimate the post-Q increment ratio as the average of the D, T, and Q ratios, which gives the post-Q correction as -0.003963, -0.003980, and -0.004134 Hartrees, for BS7-C, BS8-E and BS8-F, respectively. Adding these corrections to the composite UCCSDTQ energy, we estimate the exact UCC energy as -22140.410812, -22140.411097, and -22140.409875 Hartrees, for BS7-C, BS8-E and BS8-F, respectively.

Supplementary Table 20: Post quadruples correction for the UCC energy for BS7-C, BS8-E and BS8-F states of the LLDUC model. ΔE represents the D, T, Q and post-Q correlation increments. Estimated quantities are labeled in bold. Absolute energies are in Hartrees shifted by -116.0 Hartrees (dimer) or -22140.0 Hartrees (LLDUC).

spin isomer	theory	E_{dimer}	$\Delta E_{\mathrm{dimer}}$	E_{LLDUC}	$\Delta E_{\mathrm{LLDUC}}$	$\Delta E_{\mathrm{LLDUC}}/\Delta E_{\mathrm{dimer}}$
BS7-C	UHF	-0.322099		0.009608		
	UCCSD	-0.361322	-0.039224	-0.305389	-0.314997	8.03
	UCCSDT	-0.372305	-0.010983	-0.394946	-0.089557	8.15
	UCCSDTQ	-0.373827	-0.001522	-0.406849	-0.011903	7.82
	exact	-0.374322	-0.000495	**estimated**	**-0.003963**	**8.00**
BS8-E	UHF	-0.322099		0.016226		
	UCCSD	-0.361322	-0.039224	-0.306453	-0.322678	8.23
	UCCSDT	-0.372305	-0.010983	-0.395255	-0.088802	8.09
	UCCSDTQ	-0.373827	-0.001522	-0.407117	-0.011862	7.79
	exact	-0.374322	-0.000495	**estimated**	**-0.003980**	**8.04**
BS8-F	UHF	-0.322099		0.016720		
	UCCSD	-0.361322	-0.039224	-0.298387	-0.315107	8.03
	UCCSDT	-0.372305	-0.010983	-0.392966	-0.094580	8.61
	UCCSDTQ	-0.373827	-0.001522	-0.405741	-0.012775	8.39
	exact	-0.374322	-0.000495	**estimated**	**-0.004134**	**8.35**

3.1.10 Natural orbital occupancy

As an additional metric for the similarity between UCC and UDMRG solutions of the LLDUC model, in Supplementary Figure 13 we show the comparison between natural orbital occupancy computed at UCCSD, UCCSDT, and UDMRG level, for some representative low-energy spin-isomers. We found good agreement between the UCC and UDMRG theories.

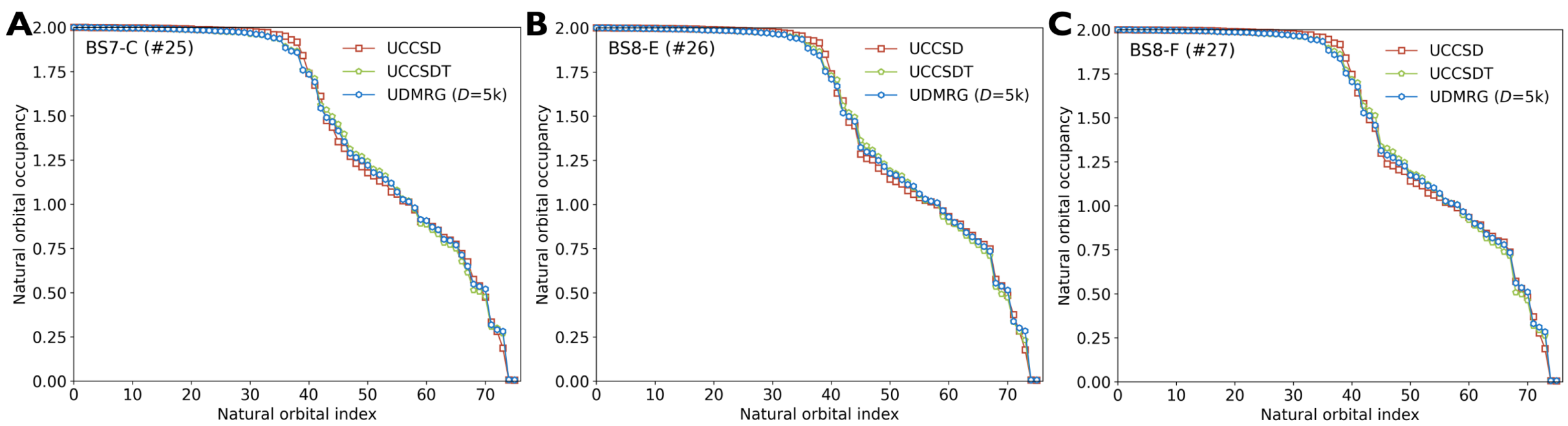

Supplementary Figure 13: **UCCSD, UCCSDT and UDMRG natural orbital occupancy computed for spin isomers BS7-C, BS8-E, and BS8-F of the LLDUC model.**

3.2 Large active space models

To investigate the effect of dynamical correlation not captured in the minimal LLDUC model, we construct a series of larger active space models and perform spin unrestricted Hartree-Fock and coupled cluster calculations (up to singles and doubles and perturbative triples) with the same cluster geometry, charge and ligands as the ones used for the LLDUC model, built from PDB 3U7Q.

3.2.1 Active space setups

We start with the high-spin UKS solution computed with the B3LYP functional (*93–95*) (as was used in the construction of the LLDUC model), the TZP-DKH basis (*96*) for Fe, Mo, and S, the def2-SVP basis (*105*) for H, C, O, and N atoms), and the remaining protein outside of the crystal cutout treated using the domain-decomposition COSMO solvation model (dd-COSMO) ($\epsilon = 4.0$) (*97–99*) as implemented in PySCF. We split-localize the spin unrestricted natural orbitals (UNO) computed from the high-spin UKS solution to doubly occupied (PM (*83*)), singly occupied (PM), and virtual (SCDM (*100*)) subspaces. We then constructed a (994o, 423e) active space including all FeMo-co orbitals (7 Fe atoms + 1 Mo atoms + 1 C atom + 10 S atoms + 2 O atoms + 1 N atom), by selecting 994 localized orbitals that are localized on the FeMo-co core.

To further truncate this active space, we performed high-spin UHF and UMP2 (*51, 101*) in the (994o, 423e) active space (without frozen core or density fitting). In Supplementary Figure 14, we show UMP2 natural orbital occupancies

computed in the (994o, 423e) active space and the orbital index ranges used to construct smaller active spaces.

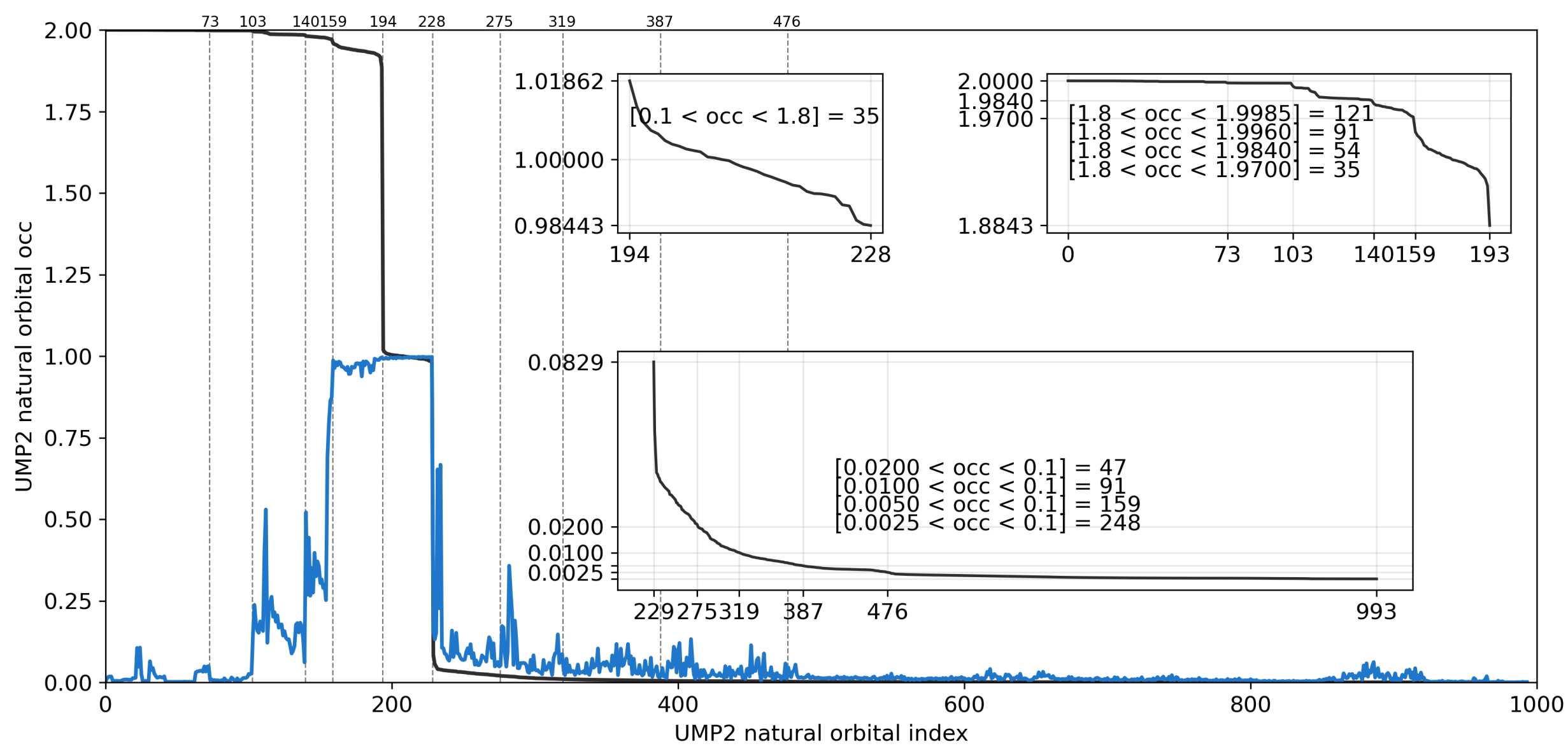

Supplementary Figure 14: **Active space constructed using UMP2 natural orbitals.** Black lines: UMP2 natural orbital occupation in the (994o, 423e) active space. Blue line: the squared overlap between each UMP2 natural orbital and the LLDUC (76o, 113e) active space.

Based on the UMP2 natural orbital occupancies, we define four truncated active spaces listed in Supplementary Table 21. the high-spin UHF and UMP2 energies in the 994o active space and the four truncated active spaces are also given in Supplementary Table 21.

Supplementary Table 21: The four truncated active spaces and the full (994o, 423e) active space. Integers are number of orbitals included in each active space. Number of frozen orbitals is counted with respect to the (994o, 423e) active space. Energies are in Hartrees.

active space	occ >1	occ ≈ 1	occ <1	frozen	E_{UHF} (high spin)	E_{UMP2} (high spin)
(117o, 105e)	35	35	47	159	-22127.108167	-22127.960360
(180o, 143e)	54	35	91	140	-22127.119741	-22128.792668
(285o, 217e)	91	35	159	103	-22127.140496	-22130.559716
(404o, 277e)	121	35	248	73	-22127.146440	-22132.433149
(994o, 423e)	194	35	765	0	-22127.157903	-22136.129826

Note that for all broken-symmetry (low-spin) UHF, UCCSD, and UCCSD(T) computations for these four larger active spaces (see below), the dd-COSMO effect is always computed using the high-spin UHF density matrix.

3.2.2 Energy of broken-symmetry solutions

To properly sample UHF solutions in the larger active spaces, we performed UHF calculations using a variety of different initial guess density matrices, followed by a second-order Newton procedure for orbital optimization. For each of the 35 broken-symmetry spin couplings, we consider: (a) 2250 possible initial diagonal density matrices; (b) around 351 unique UHF solutions in the 76o active space projected to the larger active spaces; and (c) around 24 low-energy UHF solutions in the smaller active space projected to larger active spaces.

For each of the 35 broken-symmetry spin couplings, we identified 24 low-energy UHF solutions from all unique UHF solutions. UCCSD and UCCSD(T) are performed using each of these 24 low-energy UHF as references (some high-energy spin isomers and high-UHF-energy references are skipped due to computational limitations). The lowest UHF energy, the lowest UCCSD energy, and the UCCSD(T) energy corresponding to the lowest UCCSD energy, for BS7-BS10 spin isomers in the 285o and 404o active spaces, are listed in Supplementary Table 22 and plotted in Supplementary Figure 15. We see that the UHF, UCCSD, and UCCSD(T) energy differences between the 285o and 404o active spaces are within a few kcal/mol and BS7, BS8, and BS10 are found to be low-energy spin isomers.

Supplementary Table 22: The lowest UHF energy, the lowest UCCSD energy, and the UCCSD(T) energy corresponding to the lowest UCCSD energy, for BS7-BS10 spin isomers in the (285o, 217e) and (404o, 277e) active spaces.

	(285o, 217e) active space			(404o, 277e) active space		
spin isomer	E_{UHF}	E_{UCCSD}	$E_{\mathrm{UCCSD(T)}}$	E_{UHF}	E_{UCCSD}	$E_{\mathrm{UCCSD(T)}}$
BS9-A	-22127.219879	-22130.861812	-22131.063735	-22127.232790	-22132.645370	-22132.903818
BS10-A	-22127.225183	-22130.870124	-22131.082960	-22127.234246	-22132.654036	-22132.914820
BS10-B	-22127.224936	-22130.871301	-22131.083389	-22127.234825	-22132.654566	-22132.915952
BS10-C	-22127.216069	-22130.859955	-22131.072868	-22127.230860	-22132.649132	-22132.908483
BS9-B	-22127.215523	-22130.851258	-22131.061052	-22127.229049	-22132.639196	-22132.897251
BS10-D	-22127.216123	-22130.865234	-22131.072463	-22127.227189	-22132.652592	-22132.908899
BS10-E	-22127.222899	-22130.864230	-22131.074418	-22127.236699	-22132.651886	-22132.910451
BS10-F	-22127.222076	-22130.867418	-22131.076992	-22127.237848	-22132.654382	-22132.912626
BS9-C	-22127.214306	-22130.856354	-22131.063087	-22127.224737	-22132.642246	-22132.898505
BS8-A	-22127.208606	-22130.867979	-22131.077202	-22127.227487	-22132.654885	-22132.913317
BS8-B	-22127.215925	-22130.870860	-22131.079594	-22127.231970	-22132.654821	-22132.913686
BS7-A	-22127.212998	-22130.873173	-22131.082204	-22127.230591	-22132.660383	-22132.918028
BS8-C	-22127.209400	-22130.864752	-22131.072786	-22127.225124	-22132.652915	-22132.909707
BS7-B	-22127.221262	-22130.866538	-22131.078580	-22127.237505	-22132.655413	-22132.914718
BS8-D	-22127.212274	-22130.868166	-22131.077023	-22127.221349	-22132.652052	-22132.911143
BS7-C	-22127.218461	-22130.873955	-22131.086843	-22127.236277	-22132.660328	-22132.921591
BS8-E	-22127.216441	-22130.875050	-22131.086915	-22127.228840	-22132.658308	-22132.916494
BS8-F	-22127.214312	-22130.874957	-22131.085784	-22127.222811	-22132.656077	-22132.917963

To include the effect of dynamical correction beyond the 404-orbital active space, we compute the composite energy as

$$E_{\mathrm{UCCSD}}^{\text{composite}} = E_{\mathrm{UCCSD}}^{(404\text{o},277\text{e})} + E_{\mathrm{UMP2}}^{(994\text{o},423\text{e})} - E_{\mathrm{UMP2}}^{(404\text{o},277\text{e})} \tag{S6}$$

$$E_{\mathrm{UCCSD(T)}}^{\text{composite}} = E_{\mathrm{UCCSD(T)}}^{(404\text{o},277\text{e})} + E_{\mathrm{UMP2}}^{(994\text{o},423\text{e})} - E_{\mathrm{UMP2}}^{(404\text{o},277\text{e})} \tag{S7}$$

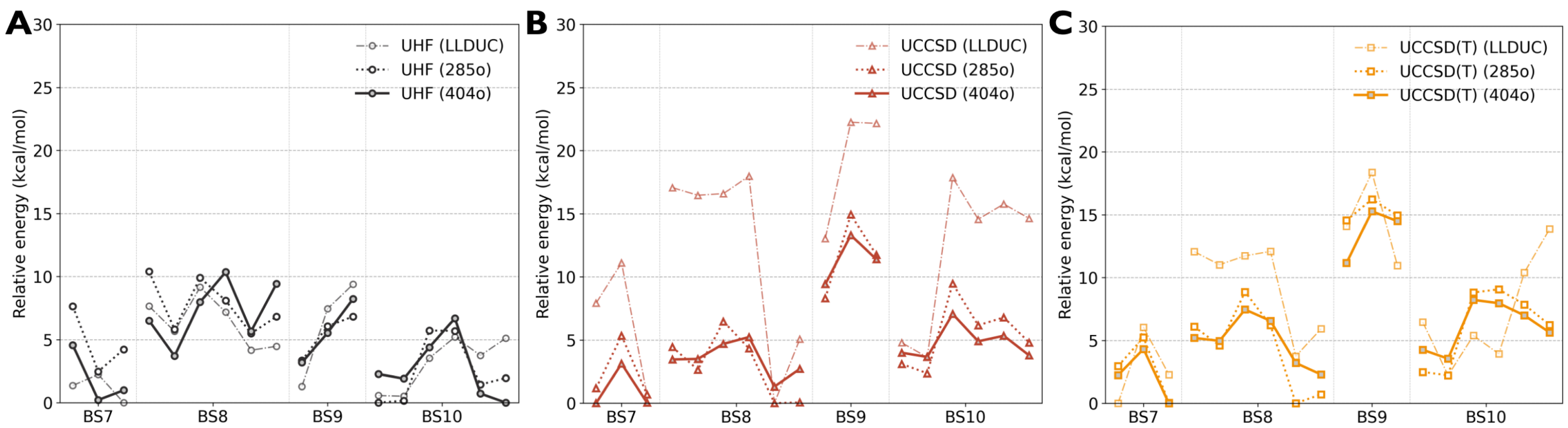

Supplementary Figure 15: **Low-energy spin isomers in different active spaces.** **A** The lowest UHF energy, **B** the lowest UCCSD energy, **C** and the UCCSD(T) energy corresponding to the lowest UCCSD energy, for BS7-BS10 spin isomers in the (285o, 217e) and (404o, 277e) active spaces.

To determine the UHF reference for UMP2 in the (994o, 423e) active space we use the projected reference corresponding to the lowest CCSD energy in the (404o, 277e) active space as the initial guess. The UMP2 composite correction and corrected UCCSD and UCCSD(T) energies are listed in Supplementary Table 23.

Supplementary Table 23: The UHF and UMP2 energies in the (994o, 423e) active space, the UMP2 energy in the (404o, 277e) active space, and the composite UCCSD and UCCSD(T) energies. $\Delta E_{\mathrm{UMP2}} = E^{(994o,423e)}_{\mathrm{UMP2}} - E^{(404o,277e)}_{\mathrm{UMP2}}$.

	(994o, 423e) active space		(404o, 277e)	composite		
spin isomer	E_{UHF}	E_{UMP2}	E_{UMP2}	ΔE_{UMP2}	$E^{\mathrm{composite}}_{\mathrm{UCCSD}}$	$E^{\mathrm{composite}}_{\mathrm{UCCSD(T)}}$
BS9-A	-22127.241126	-22136.335626	-22132.613577	-3.722049	-22136.367419	-22136.625867
BS10-A	-22127.242223	-22136.337368	-22132.617133	-3.720235	-22136.374271	-22136.635055
BS10-B	-22127.239027	-22136.337714	-22132.616968	-3.720746	-22136.375311	-22136.636698
BS10-C	-22127.249909	-22136.335728	-22132.609092	-3.726636	-22136.375768	-22136.635119
BS9-B	-22127.241007	-22136.329132	-22132.606886	-3.722246	-22136.361442	-22136.619497
BS10-D	-22127.252549	-22136.338013	-22132.615450	-3.722563	-22136.375155	-22136.631462
BS10-E	-22127.245289	-22136.344946	-22132.617834	-3.727112	-22136.378998	-22136.637563
BS10-F	-22127.248065	-22136.343599	-22132.620606	-3.722993	-22136.377375	-22136.635619
BS9-C	-22127.248769	-22136.328206	-22132.607131	-3.721075	-22136.363321	-22136.619580
BS8-A	-22127.249320	-22136.343744	-22132.618995	-3.724749	-22136.379635	-22136.638067
BS8-B	-22127.244322	-22136.341049	-22132.619851	-3.721197	-22136.376018	-22136.634883
BS7-A	-22127.255926	-22136.344732	-22132.620802	-3.723930	-22136.384314	-22136.641958
BS8-C	-22127.253498	-22136.340116	-22132.614982	-3.725134	-22136.378050	-22136.634841
BS7-B	-22127.246846	-22136.344087	-22132.618938	-3.725149	-22136.380563	-22136.639867
BS8-D	-22127.240197	-22136.335782	-22132.614788	-3.720994	-22136.373047	-22136.632138
BS7-C	-22127.242612	-22136.349251	-22132.622766	-3.726485	-22136.386813	-22136.648076
BS8-E	-22127.255794	-22136.343758	-22132.622896	-3.720862	-22136.379170	-22136.637356
BS8-F	-22127.240501	-22136.336337	-22132.618368	-3.717969	-22136.374047	-22136.635932

3.2.3 Fe oxidation states

To investigate dynamical correlation effects on the assignment of oxidation states, we compute the electron population and nuclear density on Fe atoms in the larger active spaces, and compare the results with those obtained from the LLDUC model. The results are shown in Supplementary Figures 16. The ordering of Fe centers in large active spaces is shown in Supplementary Figures 17.

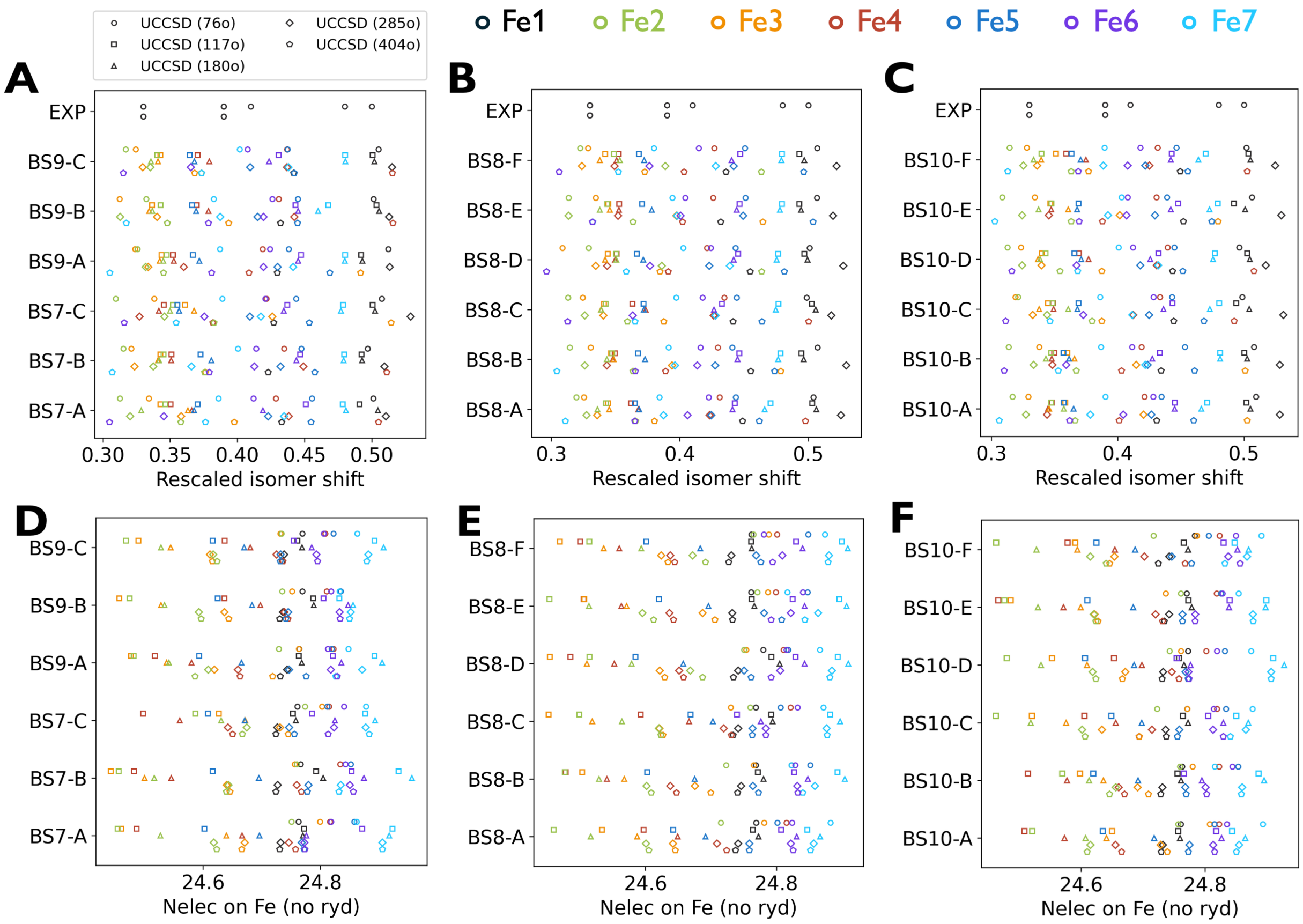

Supplementary Figure 16: **Isomer shift and electron population in different active spaces. A,B,C** Rescaled isomer shift (computed using electron density at Fe nucleus) for selected low-energy spin isomers (BS7-BS10) computed at UCCSD level with different active space models. **D,E,F** Electron population on Fe (computed using meta-Löwdin without Rydberg contributions) for selected low-energy spin isomers (BS7-BS10) computed at UCCSD level with different active space models.

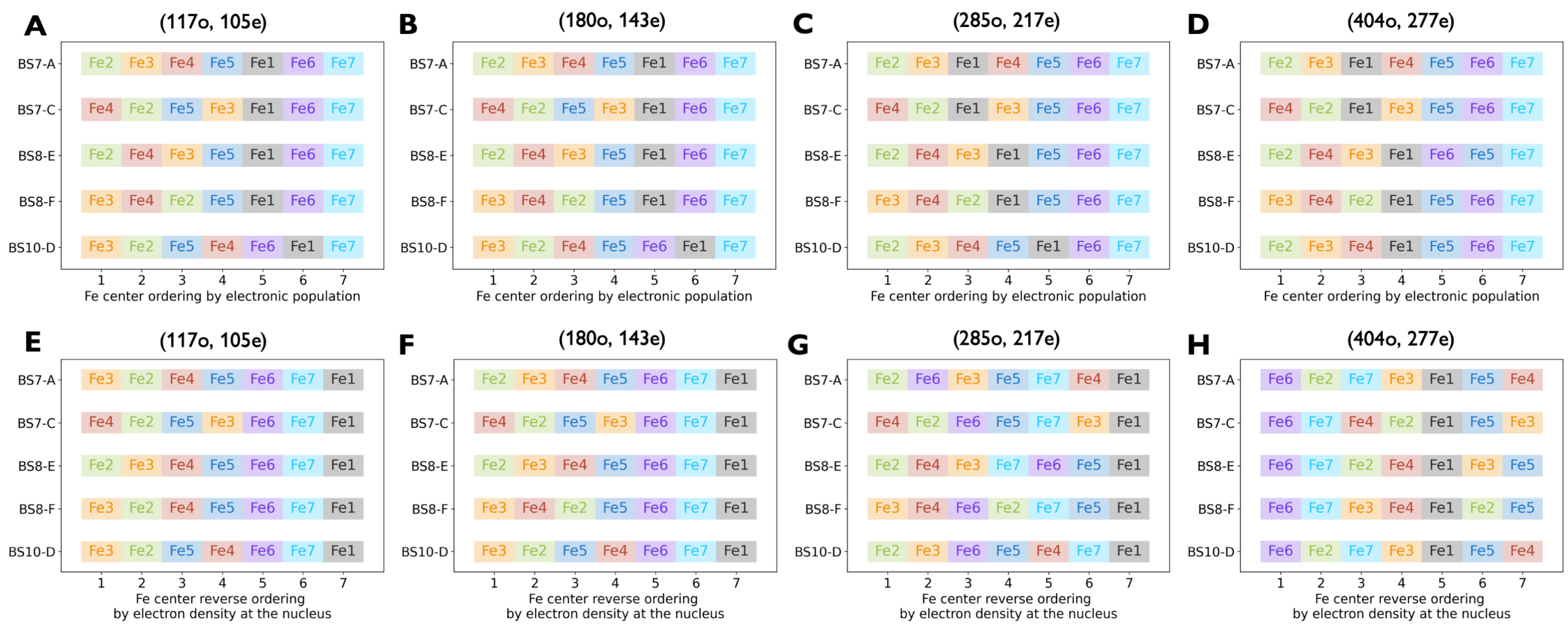

Supplementary Figure 17: **Ordering of isomer shift and electron population in different active spaces. A,B,C,D** Ordering of rescaled isomer shift (computed using electron density at Fe nucleus) for selected low-energy spin isomers computed at UCCSD level with different active space models. **E,F,G,H** Ordering of electron population on Fe (computed using meta-Löwdin without Rydberg contributions) for selected low-energy spin isomers computed at UCCSD level with different active space models.

3.2.4 Natural orbital occupancy

In Supplementary Figure 18 we show the comparison between the natural orbital occupancy computed at the UCCSD level of theory with different active spaces, for some representative low-energy spin-isomers. We found a good agreement for the occupancy in the strongly-correlated region among different active spaces. For less strongly correlated orbitals, the large active spaces show a larger deviation from exact double occupancy due to the inclusion of correlating virtual orbitals into the active space.

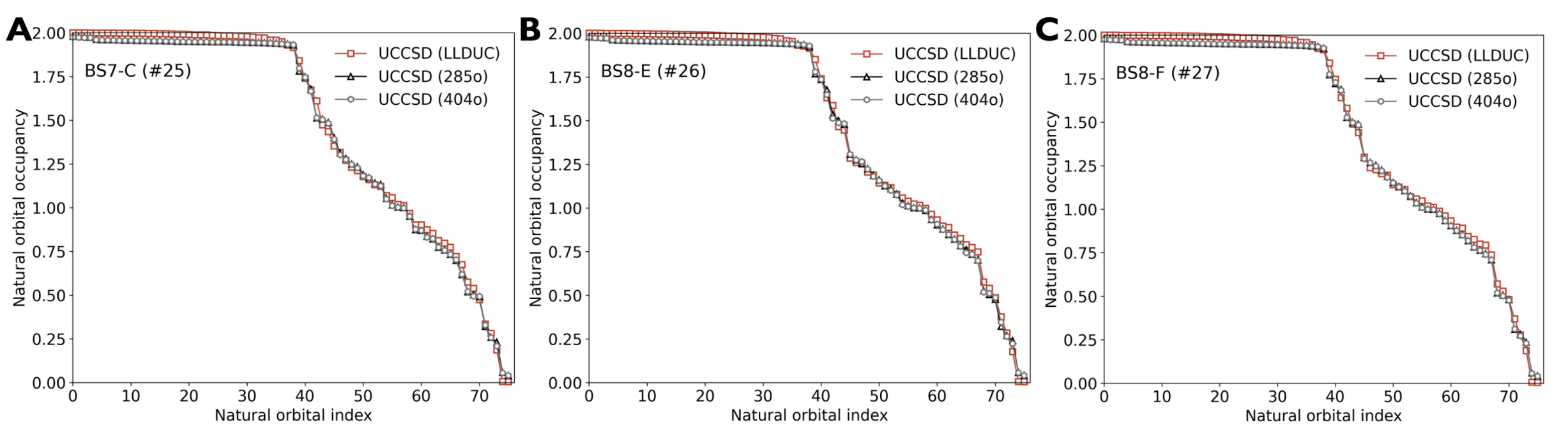

Supplementary Figure 18: **UCCSD natural orbital occupancy computed for spin isomers BS7-C, BS8-E, and BS8-F in the LLDUC, (285o, 217e), and (404o, 277e) active spaces.** For 285 and 404 orbital active spaces, only occupancy of the 76-orbital subspace corresponding to the LLDUC region is plotted.

3.3 Active space model from QM/MM

3.3.1 Molecular dynamics

The MD model was prepared from the crystal structure (PDB ID: 3U7Q (*6*)) with OpenMM (*58*) and GFN2-xTB (*59*) through a Python interface provided by ASH (*60*). The full heterotetramer of the Mo-Fe protein was included. The imidazole molecules from the buffer were removed, while the co-crystallized Mg^{2+}, Ca^{2+}, water, P-clusters, and FeMo-co were all kept. The Cys residues that coordinate with the FeMo-co and P-clusters were modeled as deprotonated, and the His residues that are close to the FeMo-co (H195:A and H195:C) were modeled as single-protonated on the ϵ-nitrogen. The homocitrate was modelled in a singly protonated state where a proton was shared between the Mo-coordinating hydroxyl group and one carboxylate oxygen atom. All other residues were assumed to be in normal protonation states under pH 7. The system was solvated in a cubic water box with a side length of ∼140 Å, and 0.1 M of sodium chloride was added.

The force field parameters for the P-cluster and FeMo-co were obtained from the universal force field (*61*) with atomic charges derived from GFN2-xTB (*59*). In xTB calculations, the P-cluster was assumed to be in the resting state, i.e., $Fe_8S_7^-$ with total spin $S = 0$, and the FeMo-co was in $Fe_7MoS_9C^-$ with $S = 3/2$. The rest of the system was described by the Amber14 force field (*62*) with TIP3P-FB water (*63*).

The system was energy minimized, equilibrated under 1 bar and 300 K for 20 ns, and with a fixed volume under 300 K for 300 ns. During minimization and MD, the geometry of the P-cluster, the FeMo-co, and a few nearby residues, as well as crystal water, were kept frozen and not moved. The frozen atoms around FeMo-co (225 atoms in total) were the same ones used to define a truncated cluster model used in the previous high-spin DFT (*9*) to derive the LLDUC model (denoted as LLDUC DFT hereafter). As such, the effects from protein and solvation water can be separated from the influence of a potential geometry change of the cluster, subject to the force field quality. All MD simulations were performed by OpenMM 8.1, using the middle Langevin integrator with a 4 fs timestep, hydrogen-involving bonds constrained, and repartitioned hydrogen masses (1.5 amu).

3.3.2 QM/MM potential

The full periodic QM/MM electrostatic potential on the FeMo-co bound in chain A was computed for the MD-sampled geometries. The MM charges were taken from their force field values, and modelled as Gaussian-distributed charges with radii taken from their covalent (*102*) and ionic (*103*) radius values. The long-range electrostatics was computed with the QM/MM-Multipole approach (*50*), using a 25 Å cutoff for the short-range electrostatics beyond quadrupoles. The electrostatics generated by the periodic QM images were also included, and their charge distribution was modeled using the LLDUC DFT density (*9*). Due to NPT equilibration, the structure of FeMo-co was slightly rescaled (RMSD=

0.057 Å for the set of 225 atoms, including FeMo-co and a few nearby residues and water from the crystal structure (and thus from the structure used in the LLDUC model). To maintain consistency, the crystal structure to generate the LLDUC model was substituted in the MD-sampled geometries for the QM/MM potential calculations. The QM/MM potential was computed every 2 ps in the same atomic basis as LLDUC DFT. The potential was first block-averaged in 2-ns-long chunks, and we computed the electrostatic energy of the LLDUC DFT density under the averaged potential to form a time series $\{e_t\}$. The time t_0 that minimized the standard error of $\{e_t | t \geq t_0\}$ was 40 ns and determined as the initial equilibration time. The standard error of e on the last 260 ns was 0.66 kcal/mol per atom. The largest absolute-valued matrix element in the standard error of the potential was 1.5 mHa, and the mean absolute value of all elements was 8×10^{-7} Hartrees.

3.3.3 Active space model

The same procedure as LLDUC was followed to derive an active space model, with the COSMO potential replaced by the sampled QM/MM potential. A high-spin DFT calculation ($S = 35/2$) with spin-free X2C and the B3LYP functional was performed, and the spin-averaged DFT natural orbitals were split-localized using Pipek-Mezey localization (*83*), then all the C 2s, 2p, Fe 3d, Mo 4d, and S 3p orbitals, along with two ligand orbitals, were selected to be in the active space, yielding a 76o active space.

3.3.4 Energy of broken-symmetry solutions

To sample UHF solutions for the 76o active space model with the QM/MM potential, for each of the 35 broken-symmetry spin couplings, we consider: (a) 2250 possible initial diagonal density matrices; (b) around 351 unique UHF solutions in the 76o LLDUC active space projected to the 76o active space with QM/MM potential. We identified 24 low-energy UHF solutions from unique UHF solutions obtained from (a) and (b). UCCSD and UCCSD(T) are performed using each the 24 low-energy UHF as references. The lowest UHF energy, the lowest UCCSD energy and the UCCSD(T) energy corresponding to the lowest UCCSD energy, for all spin isomers in the 76o active space model with QM/MM potential, are listed in Supplementary Table 24 and plotted in Supplementary Figure 19. We see that the UHF and UCCSD energies are mostly correlated across the LLDUC and QM/MM models. The UCCSD(T) energies show slightly larger fluctuations than UCCSD, partially caused by the sensitivity in the (T) correction when the T_1 diagnostic is large (see Supplementary Sec. 3.1.2).

4 4Fe-4S clusters

We examine the accuracy of the spin unrestricted DMRG and high-order CC approaches for FeMo-co models by investigating their performance on the smaller 2Fe(II)-2Fe(III) iron-sulfur cubane cluster. This has Fe atoms in

Supplementary Table 24: UHF, UCCSD, and UCCSD(T) energies with the best reference for each spin isomer of the 76o active space model with QM/MM potential. Energies are in Hartrees.

spin isomer	E_{UHF}	E_{UCCSD}	$E_{\mathrm{UCCSD(T)}}$	CCSD T_1 diagnostic
BS3-A	-1128.749828	-1129.051423	-1129.109900	0.08939
BS3-B	-1128.747568	-1129.050914	-1129.110334	0.09382
BS9-A	-1128.763149	-1129.058648	-1129.110599	0.09186
BS10-A	-1128.764822	-1129.075438	-1129.128793	0.09264
BS10-B	-1128.759424	-1129.075676	-1129.126760	0.09846
BS3-C	-1128.735494	-1129.041570	-1129.091090	0.08658
BS10-C	-1128.760007	-1129.056808	-1129.129654	0.13623
BS9-B	-1128.752469	-1129.047591	-1129.108324	0.12632
BS10-D	-1128.753904	-1129.061313	-1129.130782	0.14911
BS10-E	-1128.760105	-1129.055396	-1129.114975	0.10091
BS10-F	-1128.758531	-1129.068612	-1129.128012	0.08651
BS9-C	-1128.750223	-1129.049912	-1129.107509	0.10196
BS6-A	-1128.739521	-1129.037462	-1129.089403	0.09699
BS6-B	-1128.734076	-1129.035167	-1129.089990	0.09890
BS6-C	-1128.730396	-1129.049856	-1129.098651	0.09151
BS2	-1128.741752	-1129.057890	-1129.103049	0.09519
BS8-A	-1128.755322	-1129.060500	-1129.115902	0.09005
BS8-B	-1128.756467	-1129.058977	-1129.117878	0.09125
BS7-A	-1128.759225	-1129.068524	-1129.126342	0.09288
BS8-C	-1128.751086	-1129.057622	-1129.115664	0.10431
BS7-B	-1128.761941	-1129.068528	-1129.127390	0.11206
BS8-D	-1128.754527	-1129.054647	-1129.114224	0.11251
BS5-A	-1128.739661	-1129.022878	-1129.089226	0.19220
BS5-B	-1128.739819	-1129.024689	-1129.080697	0.10866
BS4-A	-1128.743246	-1129.048552	-1129.103613	0.09069
BS7-C	-1128.760625	-1129.080084	-1129.128564	0.08787
BS8-E	-1128.756306	-1129.075725	-1129.125222	0.08719
BS8-F	-1128.756161	-1129.072127	-1129.123413	0.09122
BS5-C	-1128.746947	-1129.036154	-1129.096812	0.15114
BS4-B	-1128.754599	-1129.054236	-1129.142001	0.10089
BS5-D	-1128.746839	-1129.043997	-1129.099306	0.08231
BS4-C	-1128.754485	-1129.059842	-1129.115663	0.14681
BS5-E	-1128.748739	-1129.048206	-1129.123704	0.09940
BS5-F	-1128.746401	-1129.051020	-1129.144301	0.11775
BS1	-1128.710228	-1128.980490	-1129.026409	0.10471

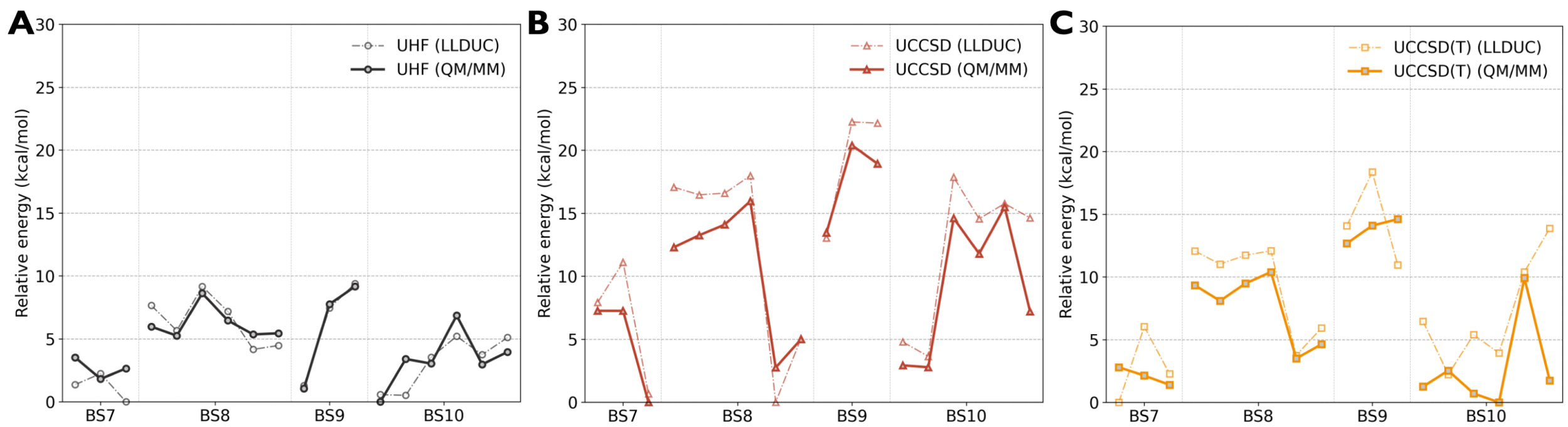

Supplementary Figure 19: **Low-energy spin isomers with QM/MM potential.** **A** The lowest UHF energy, **B** the lowest UCCSD energy, **C** and the UCCSD(T) energy corresponding to the lowest UCCSD energy, for BS7-BS10 spin isomers in the 76o active space model with QM/MM potential.

different oxidation states and near-degenerate low-energy spin configurations, representing typical computational challenges in these iron-sulfur systems. The (36o, 54e) active space model was defined in Ref. (*22*) and the integral file can be obtained from https://github.com/zhendongli2008/Active-space-model-for-Iron-Sulfur-Clusters/blob/main/Fe2S2_and_Fe4S4/Fe4S4/fe4s4. Note that the core energy -8105.56038966 Hartrees for this active space model is not included in the reported total energy.

4.1 Spin-adapted DMRG and extrapolations

First we compute the ground-state energy for one of the spin isomers of the (36o, 54e) active space model of $[Fe_4S_4(SCH_3)_4]^{2-}$ using spin-adapted DMRG up to a large bond dimension $D = 20000$ (number of spin multiplets) starting from MPS initial guesses generated using spin-projected DMRG, (*22*) which gives variational upper bounds of $E^{\text{SA-DMRG}}_{\text{variational}} = -327.244142, -327.246083$, and -327.246141 Hartrees, for the BS1, BS2, and BS3 states initial guesses, respectively. From the one- and two-particle density matrices, we identify the SA-DMRG state starting from the "BS1" spin isomer guess as corresponding to the configuration [Fe1↑, Fe2↑, Fe3↓, Fe4↓] (see Supplementary Figure 20), which in the subsequent section, we name "BS1" even though no symmetry is broken. (This is because the broken symmetry solution is obtained by mixing spin-adapted states in a specific tower of states associated with that spin isomer, and the spin-adapted solution is the ground-state within that tower of states.) The SA-DMRG solution starting from the "BS2" and "BS3" initial guesses converges to the same state which is a mixture of BS2 and BS3 spin patterns. Hence, in the remainder of this subsection we do not treat "BS2" and "BS3" as distinct SA-DMRG solutions.

Starting from the $D = 20000$ solution, we perform discarded weight based extrapolation for energies from a reverse schedule with bond dimensions $D = 18000$ to $D = 14000$, which gives an extrapolated ground-state energy

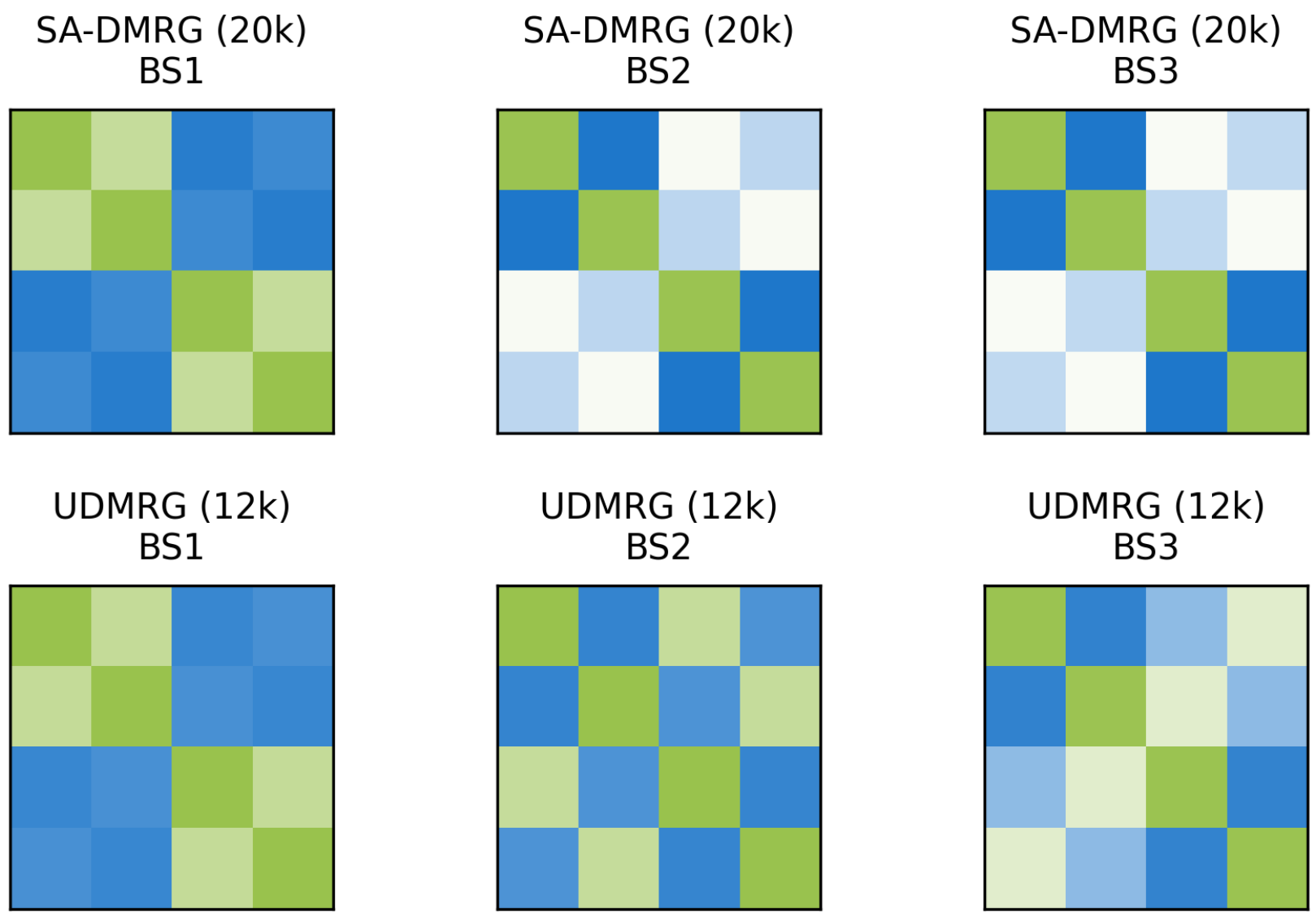

Supplementary Figure 20: **The spin correlation pattern computed for the SA-DMRG state at $D = 20000$ starting from different initial guesses, and the three UDMRG states at $D = 12000$ for the (36o, 54e) active space model of the 2Fe(II)-2Fe(III) iron-sulfur cubane cluster.**

estimate of $E_{\text{extrap}}^{\text{SA-DMRG}} = -327.245342$ and -327.248524 Hartrees for BS1 and BS3, respectively. We list spin-adapted DMRG energies in Supplementary Table 25. The energy extrapolation for spin-adapted DMRG is shown in Supplementary Figure 21. We also perform bond dimension extrapolation using $\exp[-\kappa(\log D)^2]$ fitting, to give $E_{\text{extrap}}^{\text{SA-DMRG}} = -327.245287$ and -327.248841 Hartrees for BS1 and BS3, which agrees very well with the extrapolated energy using maximal discarded weights. We will compare the energy obtained using spin-adapted DMRG to those from various broken symmetry estimates used in this work.

Entanglement-minimized orbitals. We also performed spin-adapted DMRG with entanglement-minimized orbitals (EMO) (*31*) and a large bond dimension ($D = 20000$) to further reduce the error in the ground-state energy estimate. The EMOs were optimized with $D = 100$ for each spin isomer. The EMO-DMRG calculations were performed using the spin-adapted DMRG implementation in the FOCUS package with the support of GPU acceleration (*35*). We obtain tighter variational upper bound energies at $D = 20000$ as $E_{\text{variational}}^{\text{EMO-DMRG}} = -327.244733, -327.246852$ and -327.247036 Hartrees, for BS1, BS2, and BS3, respectively. With the $\exp[-\kappa(\log D)^2]$ extrapolation using the forward schedule sweep energy data (up to bond dimension $D = 20000$), we have $E_{\text{extrap}}^{\text{EMO-DMRG}} = -327.246726, -327.248835$ and -327.248858 Hartrees, for BS1, BS2, and BS3, respectively.

Supplementary Table 25: Spin-adapted DMRG energies and discarded weights for the 2FeII-2FeIII iron-sulfur cubane cluster active space model.

initial guess	schedule type	D	energy (Hartree)	max discarded weight
BS1	forward	20000	-327.244142	1.52×10^{-5}
	reverse	18000	-327.244025	1.99×10^{-5}
		17000	-327.243945	2.19×10^{-5}
		16000	-327.243850	2.35×10^{-5}
		15000	-327.243744	2.47×10^{-5}
		14000	-327.243626	2.61×10^{-5}
	extrapolated	∞	-327.245342	
BS2	forward	20000	-327.246083	3.36×10^{-5}
BS3	forward	20000	-327.246141	4.07×10^{-5}
	reverse	18000	-327.245860	4.24×10^{-5}
		17000	-327.245704	4.57×10^{-5}
		16000	-327.245528	4.87×10^{-5}
		15000	-327.245332	5.16×10^{-5}
		14000	-327.245113	5.45×10^{-5}
	extrapolated	∞	-327.248524	

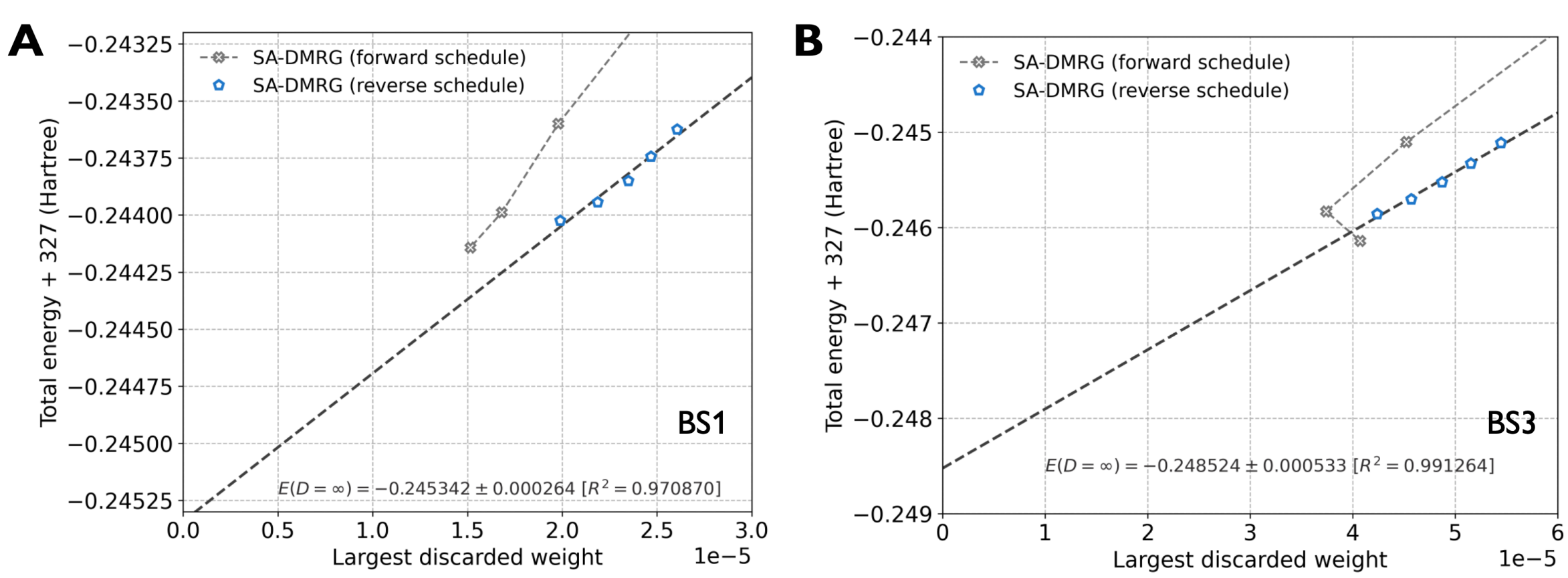

Supplementary Figure 21: **The spin-adapted DMRG energy extrapolation for the “BS1” and “BS3” spin isomers of 2FeII-2FeIII iron-sulfur cubane cluster active space model.** Note that the largest discarded weight from forward-schedule DMRG can typically be uninformative, as the state is not fully converged.

4.2 UHF, UCC, and UDMRG energies

We next consider the broken-symmetry treatment for the 2Fe(II)-2Fe(III) iron-sulfur cubane cluster, following the same protocol discussed in Sec. 3.1.2 to enumerate UHF solutions and filter references for UCC and UDMRG calculations. The 4Fe–4S cluster has three different spin isomers, denoted BS1, BS2, and BS3. We identified 11, 9, and 6 unique low-energy UHF solutions respectively for the three BS states (denoted BSn-m), with their UHF, UCCSD, and UCCSD(T) energies listed in Supplementary Table 26. We found that the ranking based on UHF energies is very different from that based on UCCSD energies. Noticeably, the BS2-8 state has the lowest UCCSD and UCCSD(T) energies, but its UHF energy is the highest among all UHF local minima. In contrast, the UCCSD(T) energies mostly track the UCCSD energies for this system.

Supplementary Table 26: Broken-symmetry UHF energies, and UCCSD and UCCSD(T) energies computed using different references for the 2FeII-2FeIII iron-sulfur cubane cluster active space model. The reference with the lowest UCCSD energies for each spin isomer is shown in bold.

spin configuration	reference	E_{UHF}	E_{UCCSD}	$E_{\mathrm{UCCSD(T)}}$
Fe1↑, Fe2↑, Fe3↓, Fe4↓	BS1-0	-327.084355	-327.197168	-327.215914
	BS1-1	-327.084217	-327.197124	-327.215909
	BS1-2	-327.083505	-327.199392	-327.215847
	BS1-3	-327.083499	-327.197465	-327.216088
	**BS1-4**	**-327.082953**	**-327.199448**	**-327.215860**
	BS1-5	-327.082836	-327.198511	-327.217398
	BS1-6	-327.082766	-327.197089	-327.215605
	BS1-7	-327.082759	-327.196287	-327.214866
	BS1-8	-327.082038	-327.195571	-327.214466
	BS1-9	-327.080859	-327.193691	-327.210521
	BS1-10	-327.079407	-327.173790	-327.184312
Fe1↑, Fe2↓, Fe3↑, Fe4↓	BS2-0	-327.084303	-327.200126	-327.216413
	BS2-1	-327.083894	-327.196582	-327.215204
	BS2-2	-327.083452	-327.199640	-327.215888
	BS2-3	-327.083187	-327.196116	-327.214926
	BS2-4	-327.082796	-327.196029	-327.215001
	BS2-5	-327.082733	-327.195510	-327.214555
	BS2-6	-327.081548	-327.194452	-327.211403
	BS2-7	-327.079922	-327.173458	-327.184170
	**BS2-8**	**-327.079094**	**-327.209283**	**-327.221857**
Fe1↑, Fe2↓, Fe3↓, Fe4↑	**BS3-0**	**-327.089868**	**-327.199165**	**-327.213268**
	BS3-1	-327.088966	-327.198048	-327.211723
	BS3-2	-327.088874	-327.198034	-327.212002
	BS3-3	-327.088412	-327.197841	-327.211844
	BS3-4	-327.080834	-327.191994	-327.209547
	BS3-5	-327.080211	-327.192248	-327.209510

4.3 Estimation of exact energies

We now consider higher-order UCC and UDMRG for the three spin isomers with references corresponding to the lowest UCCSD energies, which are BS1-4, BS2-8, and BS3-0. Their UHF and UCC (up to UCCSDTQ) energies are listed in Supplementary Table 27. We plot the UCC and UDMRG energies (relative to the "BS3" SA-DMRG energy $E_{\text{extrap}}^{\text{SA-DMRG}}$) in Supplementary Figure 22. We find that BS2 remains the lowest energy state at the UCCSDT level, but the ordering changes at the UCCSDTQ and UDMRG level. The energy spread of spin isomers decreases with increasing correlation level.

We list the UDMRG energies for the three BS states in Supplementary Table 28, and plot the bond dimension extrapolation in Supplementary Figure 23. For BS1, the UDMRG extrapolated energy of -327.244309 Hartrees is slightly higher than the SA-DMRG extrapolated energy ($\sim$1 mHa) even with a modest extrapolation distance ($\sim$3 mHa), which gives a sense of the extrapolation accuracy in these challenging systems. In BS2 and BS3, the UDMRG extrapolated energies are 1.0 mHa and 3.6 mHa below the UCCSDTQ energies. This suggests that the pentuples and higher correlations are negative. As an additional evidence, the extrapolated EMO-DMRG energies are 0.6 mHa, 4.3 mHa, and 2.5 mHa below the UCCSDTQ energies, for BS1, BS2, and BS3, respectively.

We next carry out an estimation of the exact energy from UCC using a Fe dimer reference system as we did for FeMo-co (see Sec. 3.1.9). For this we take the (20o, 31e) Fe(III)–Fe(II) dimer active space model (see Sec. 3.1.4), and we show the correlation energy increments, ratios, and predicted exact energies for BS1, BS2, and BS3, in Supplementary Table 27. We note that the correlation increment ratios are much less uniform for the cubane than they are for FeMo-co. This suggests the dimer is less good of a model system here.

Taking the consensus prediction as the average of the UCC and UDMRG predictions, we finally obtain -327.246177 ± 0.001868 Hartrees, -327.245781 ± 0.000240 Hartrees, -327.249366 ± 0.000653 Hartrees, for BS1, BS2, and BS3, respectively. More conservatively, we can estimate the energy to lie between the UCCSDTQ upper bounds and the lowest energy extrapolations i.e. the ranges $[-327.246078, -327.248046]$, $[-327.244556, -327.246022]$, and $[-327.246379, -327.250019]$ Hartrees for BS1, BS2, BS3 respectively, or -327.247062 ± 0.000984 Hartrees for BS1, -327.245289 ± 0.000733 Hartrees for BS2, and -327.248199 ± 0.001820 Hartrees for BS3. The energy difference between the composite UCC/UDMRG estimate and the EMO-DMRG extrapolation is 0.3 mHa, 3.5 mHa, 0.6 mHa for BS1, BS2, and BS3, respectively. Overall, the close correspondence between the UCC and UDMRG extrapolations, and the tightness of the UCCSDTQ upper bounds, suggests that our protocol predicts the exact energy with estimated errors of $\sim$1 kcal/mol.

Supplementary Table 27: UHF, UCC, and post quadruples correction for the UCC energy for BS1, BS2, and BS3 states computed using references with the lowest UCCSD energies (BS1-4, BS2-8, and BS3-0) for the 2FeII-2FeIII iron-sulfur active space model. ΔE represents the D, T, Q and post-Q correlation increments. Estimated quantities are labeled in bold. Absolute energies are in Hartrees shifted by -116.0 Hartrees (dimer) or -327.0 Hartrees (2FeII-2FeIII).

spin isomer	theory	E_{dimer}	$\Delta E_{\mathrm{dimer}}$	E_{4Fe}	ΔE_{4Fe}	$\Delta E_{\mathrm{4Fe}}/\Delta E_{\mathrm{dimer}}$
BS1	UHF	-0.322099		-0.082953		
	UCCSD	-0.361322	-0.039224	-0.199448	-0.116494	2.97
	UCCSD(T)	-0.367095		-0.215860		
	UCCSDT	-0.372305	-0.010983	-0.237763	-0.038315	3.49
	UCCSDTQ	-0.373827	-0.001522	-0.246078	-0.008314	5.46
	exact	-0.374322	-0.000495	**-0.248046**	**-0.001968**	**3.97**
BS2	UHF	-0.322099		-0.079094		
	UCCSD	-0.361322	-0.039224	-0.209283	-0.130189	3.32
	UCCSD(T)	-0.367095		-0.221857		
	UCCSDT	-0.372305	-0.010983	-0.240411	-0.031128	2.83
	UCCSDTQ	-0.373827	-0.001522	-0.244556	-0.004145	2.72
	exact	-0.374322	-0.000495	**-0.246022**	**-0.001466**	**2.96**
BS3	UHF	-0.322099		-0.089868		
	UCCSD	-0.361322	-0.039224	-0.199165	-0.109298	2.79
	UCCSD(T)	-0.367095		-0.213268		
	UCCSDT	-0.372305	-0.010983	-0.233939	-0.034774	3.17
	UCCSDTQ	-0.373827	-0.001522	-0.246379	-0.012440	8.17
	exact	-0.374322	-0.000495	**-0.248712**	**-0.002332**	**4.71**

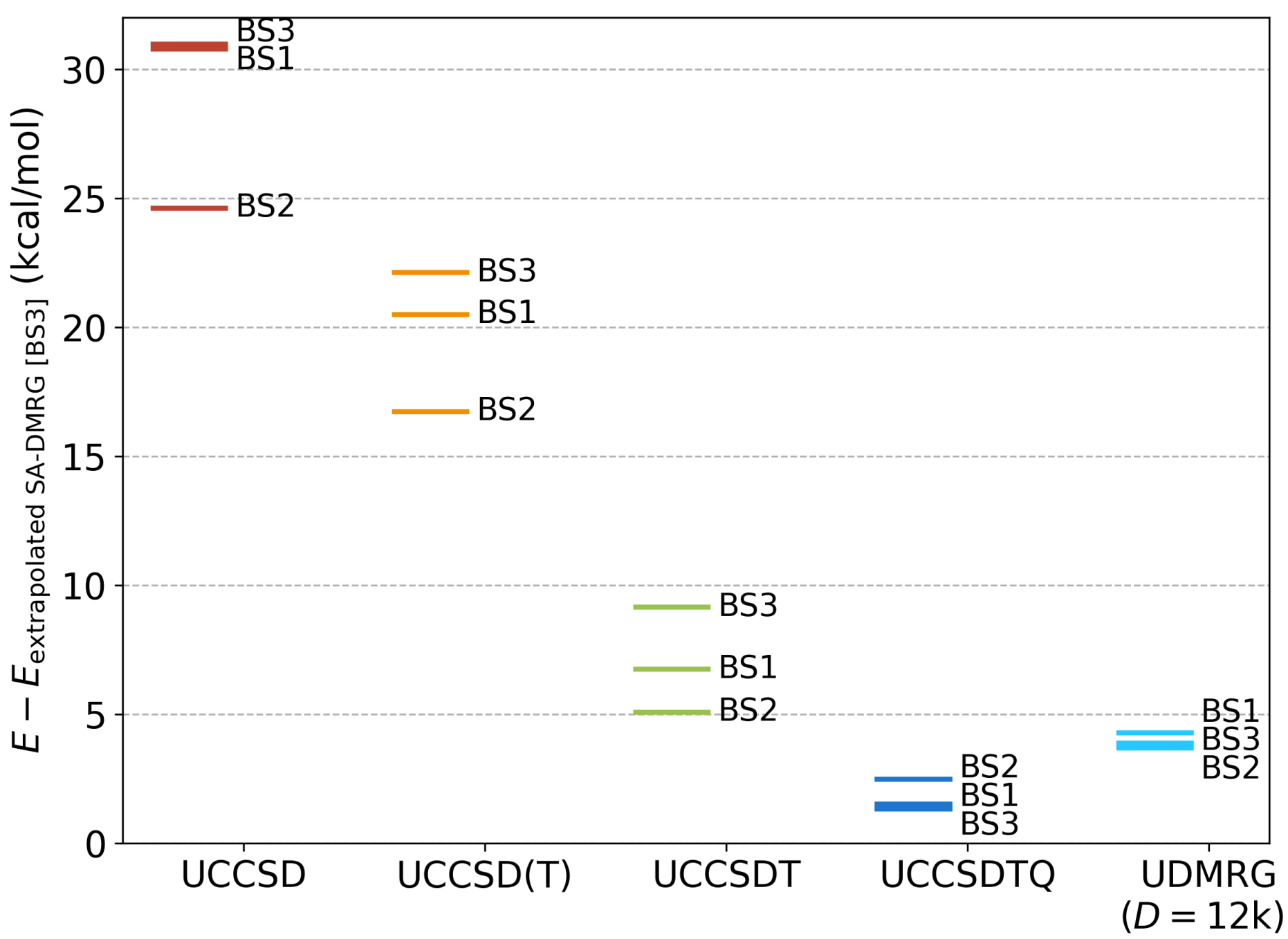

Supplementary Figure 22: **UCC and UDMRG energy error (estimated using the difference from BS3 SA-DMRG energy $E_{\mathrm{extrap}}^{\mathrm{SA\text{-}DMRG}}$) for the 2FeII-2FeIII iron-sulfur cubane cluster active space model.**

Supplementary Table 28: UDMRG energies and discarded weights for the 2FeII-2FeIII active space model. $\Delta E_{\text{UDMRG}} = E_{\text{UDMRG}} - E_{\text{extrap}}^{\text{SA-DMRG}}$. $E_{\text{extrap}}^{\text{SA-DMRG}}$ for "BS2" is taken to be the same as that for "BS3". Energies are in Hartrees.

spin isomer	schedule type	D	E_{UDMRG}	max discarded weight	ΔE_{UDMRG}
BS1	forward	12000	-327.241697	2.75×10^{-5}	+0.003646
	reverse	10000	-327.241407	3.69×10^{-5}	
	reverse	9000	-327.241208	4.12×10^{-5}	
	reverse	8000	-327.240971	4.49×10^{-5}	
	reverse	7000	-327.240688	4.82×10^{-5}	
	reverse	6000	-327.240340	5.19×10^{-5}	
	reverse	5000	-327.239894	5.63×10^{-5}	
	reverse	4500	-327.239627	5.81×10^{-5}	
	reverse	4000	-327.239304	6.12×10^{-5}	
	reverse	3500	-327.238913	6.47×10^{-5}	
	reverse	3000	-327.238426	6.89×10^{-5}	
	$\exp[-\kappa(\log D)^2]$ fit	∞	-327.244309		+0.001033
BS2	forward	12000	-327.242634	3.35×10^{-5}	+0.005889
	reverse	10000	-327.242327	4.29×10^{-5}	
	reverse	9000	-327.242123	4.61×10^{-5}	
	reverse	8000	-327.241885	4.89×10^{-5}	
	reverse	7000	-327.241604	5.15×10^{-5}	
	reverse	6000	-327.241264	5.43×10^{-5}	
	reverse	5000	-327.240831	5.79×10^{-5}	
	reverse	4500	-327.240576	5.90×10^{-5}	
	reverse	4000	-327.240267	6.12×10^{-5}	
	reverse	3500	-327.239892	6.42×10^{-5}	
	reverse	3000	-327.239426	6.81×10^{-5}	
	$\exp[-\kappa(\log D)^2]$ fit	∞	-327.245541		+0.002983
BS3	forward	12000	-327.242319	8.29×10^{-5}	+0.006204
	reverse	10000	-327.241750	9.50×10^{-5}	
	reverse	9000	-327.241403	9.74×10^{-5}	
	reverse	8000	-327.241009	1.00×10^{-4}	
	reverse	7000	-327.240555	1.03×10^{-4}	
	reverse	6000	-327.240017	1.07×10^{-4}	
	reverse	5000	-327.239355	1.12×10^{-4}	
	reverse	4500	-327.238975	1.12×10^{-4}	
	reverse	4000	-327.238523	1.15×10^{-4}	
	reverse	3500	-327.237985	1.20×10^{-4}	
	reverse	3000	-327.237326	1.25×10^{-4}	
	$\exp[-\kappa(\log D)^2]$ fit	∞	-327.250019		-0.001496

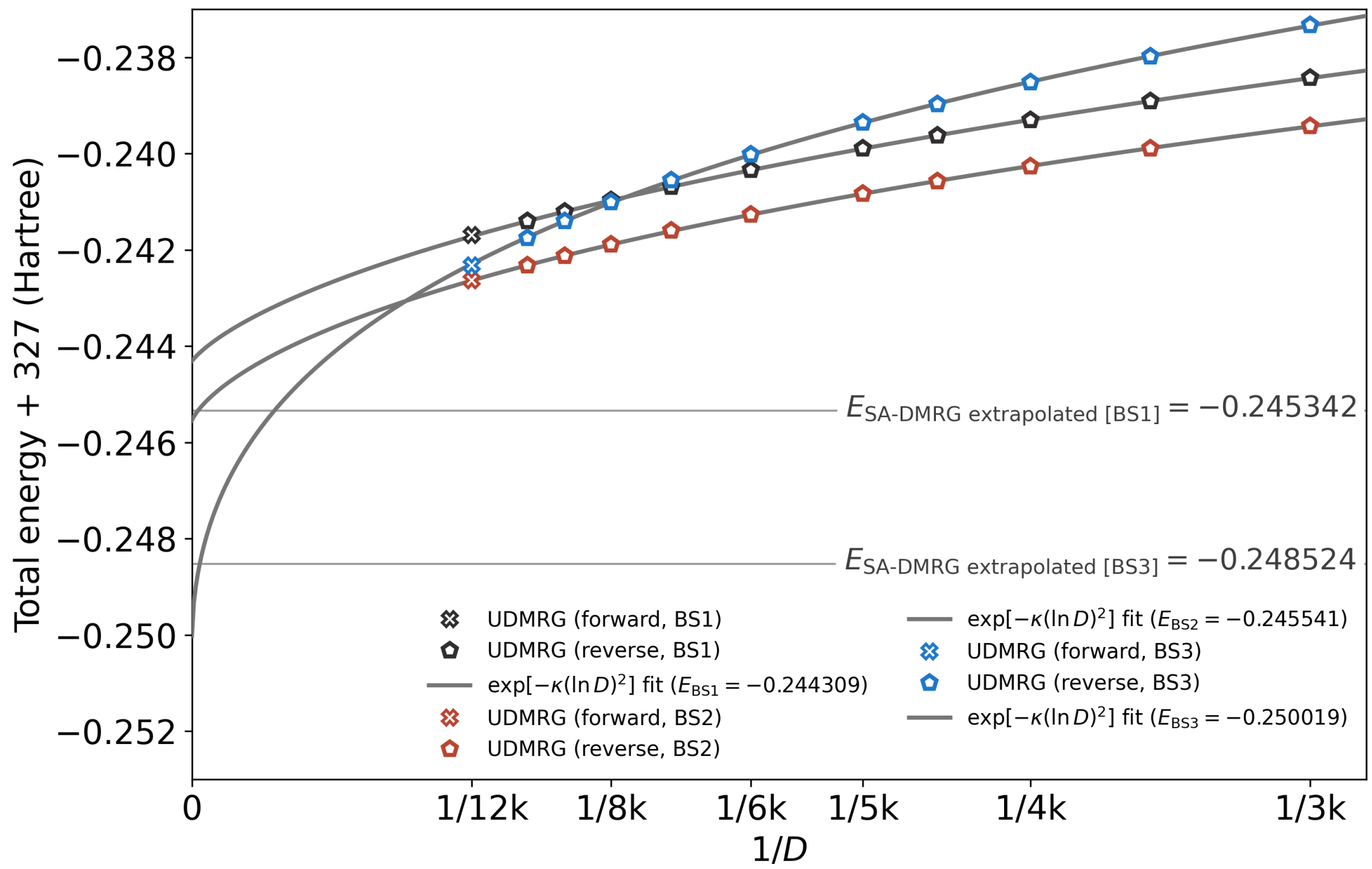

Supplementary Figure 23: **The $\exp[-\kappa(\log D)^2]$ fitting for UDMRG energies for the (36o, 54e) active space model of the 2Fe(II)-2Fe(III) iron-sulfur cubane cluster.**

5 Oxidation state calibration

We performed broken-symmetry DFT and correlated wavefunction calculations on 2Fe-2S clusters ($Fe_2S_2(SCH_3)_4{}^{3-}$) and a model of the P-cluster to establish a scale to correlate iron oxidation states with the electronic structure, and to test the sensitivity to computational settings. For Mo(III) we use $[\mathrm{MoFe_3S_4(SH)_6}]^{3-}$ to set the scale for the oxidation states.

5.1 Description of Meta-Löwdin procedure

Meta-Löwdin population analysis is a form of Löwdin population analysis where the atomic basis is first projected (using a reference set of atomic natural orbitals) so that individual atomic orbitals have core, valence, and Rydberg character. The Löwdin analysis is then carried out in the core, valence, and Rydberg spaces separately (*64*).

5.2 Meta-Löwdin population sensitivity

We tested the robustness of the meta-Löwdin population on 2Fe-2S clusters by simulating an extensive combination of total charge and spin states, computational basis sets, and active space sizes (controlled by the number of frozen core orbitals). Meta-Löwdin analysis shows that the iron populations are sensitive to the computational basis, which can be

eliminated by only counting the electrons in the core and valence space (and thus not the Rydberg contributions). For example, in the high-spin Fe(III)-Fe(II) state (total charge = -3 and $S = 9/2$), meta-Löwdin analysis on MP2/aug-cc-pwCVTZ density gives 24.98 and 24.93 electrons on the two irons, while giving 24.29 and 24.77 for the MP2/TZP-DKH density. After removing Rydberg contributions, meta-Löwdin analysis gives 23.96 and 24.52 on MP2/aug-cc-pwCVTZ and 24.00 and 24.55 on MP2/TZP-DKH, so the basis dependency is largely suppressed and the Fe(III)-Fe(II) charge ordering is consistently retained.

5.3 Fe electron population scale

We first tested if electron populations on the 2Fe-2S cluster are transferable to the P-cluster using DFT. Calculations were performed in ORCA 6.1.0 with the following input:

```
! UKS B3LYP NORI old-TZVP DKH2 DEFGRID2 TightSCF NOCOSX SlowConv CPCMC
```

to best reproduce the settings in Ref. (*16*) using an older version of ORCA. Our meta-Löwdin analysis, without Rydberg contributions, gives 24.94 for both irons in high-spin, both ferrous 2Fe-2S (total charge = -4 and $S = 4$). The same DFT and population analysis gives close to 24.97 on average over the eight irons in the P-cluster normal state (total charge = -4 and $S = 4$; geometry obtained from Supplementary Section 4.1 of Ref. (*23*)).

To establish the scales of Fe^{2+} and Fe^{3+} electron populations, we performed UCCSD on the 2Fe-2S cluster in both Fe(II)-Fe(II) (total charge = -4) and Fe(III)-Fe(III) (total charge = -2) states. We performed calculations using different basis sets, with and without frozen core, both with high-spin and low-spin, and obtained the average Fe^{3+} and Fe^{2+} populations and standard deviations: 24.53 ± 0.04 and 24.83 ± 0.02, respectively. All the calculations used the geometry from supplementary Table 1 of Ref. (*32*). For $Fe^{2.5+}$, we first performed high-spin ($S = 9/2$) calculations with a total charge of -3 on the same geometry. Due to the small geometric asymmetry, the populations on the two irons are not exactly the same. To make the two irons strictly equivalent, we additionally optimized the geometry using B3LYP with ddCOSMO, and then performed UCCSD with a total charge of -3 and $S = 9/2$, both with enforced C2h symmetry. The resulting population statistics over all the $Fe^{2.5+}$ data is 24.66 ± 0.04. These values are then scaled by the ratio between the DFT P-cluster charge and the 2Fe-2S charge (24.97/24.94) to better connect to FeMo-co oxidation states.

5.4 Nucleus electron density sensitivity

In general, the electron density values at the Fe nuclei are sensitive to the computational basis and active space sizes. However, we found that the changes correspond to constant shifts and scaling factors, and thus do not change the ordering between irons. We demonstrate this in Supplementary Figure 24.

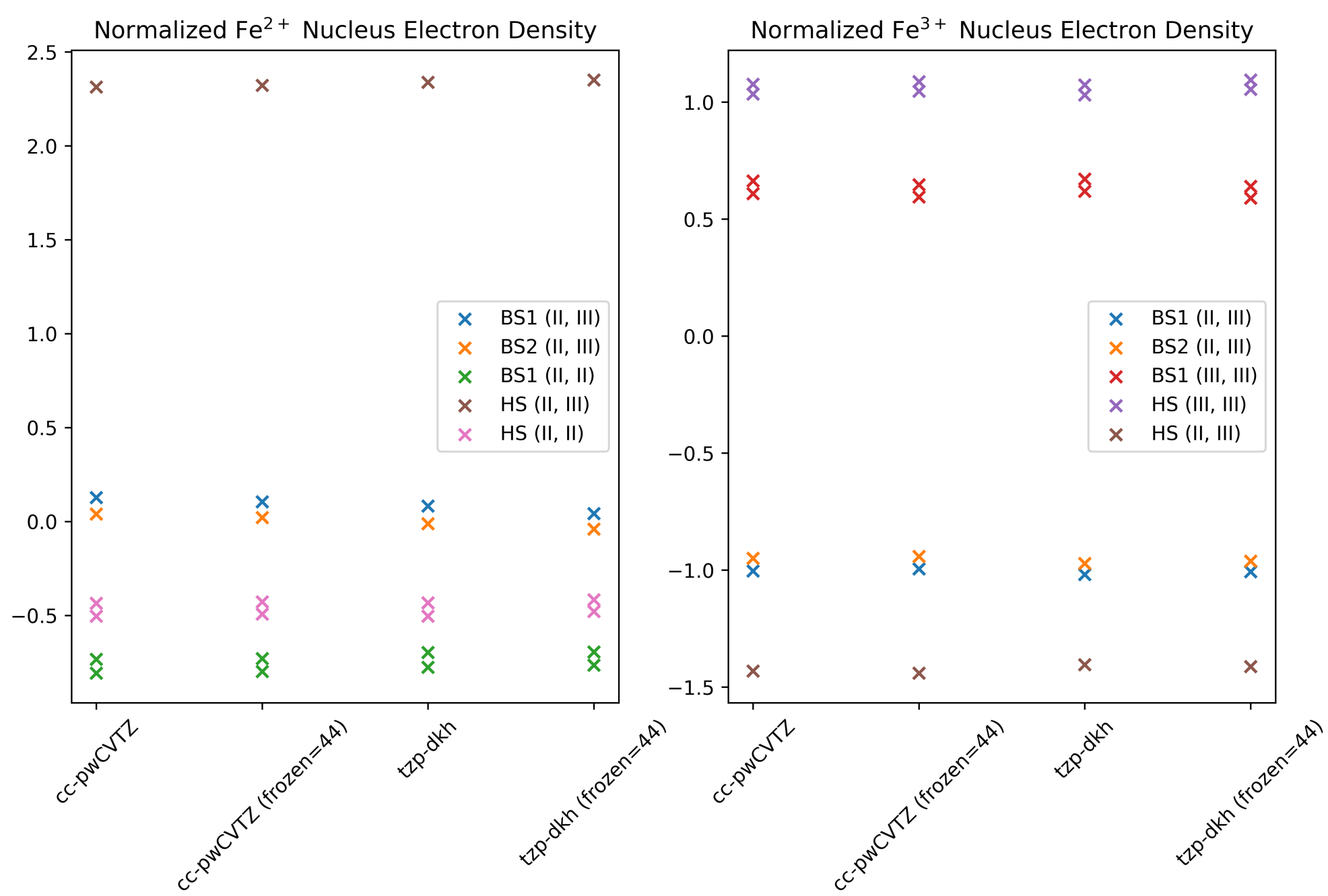

Supplementary Figure 24: **Nucleus electron density sensitivity in [2Fe2S].** Electron density values for Fe^{2+} and Fe^{3+} at the iron nuclei in 2Fe-2S clusters from UCCSD calculations in the high spin state (labeled "HS") or broken spin symmetry states (labeled "BSn" with n indicating the iron that is spin-flipped), with different iron basis sets (def2-SVP basis fixed for other atoms), with and without 44 frozen core orbitals, and different total charges (-2 for "III, III", -3 for "II, III" and -4 for "II, II"). The values are normalized by subtracting the mean and scaled by the standard deviation to show only the iron ordering.

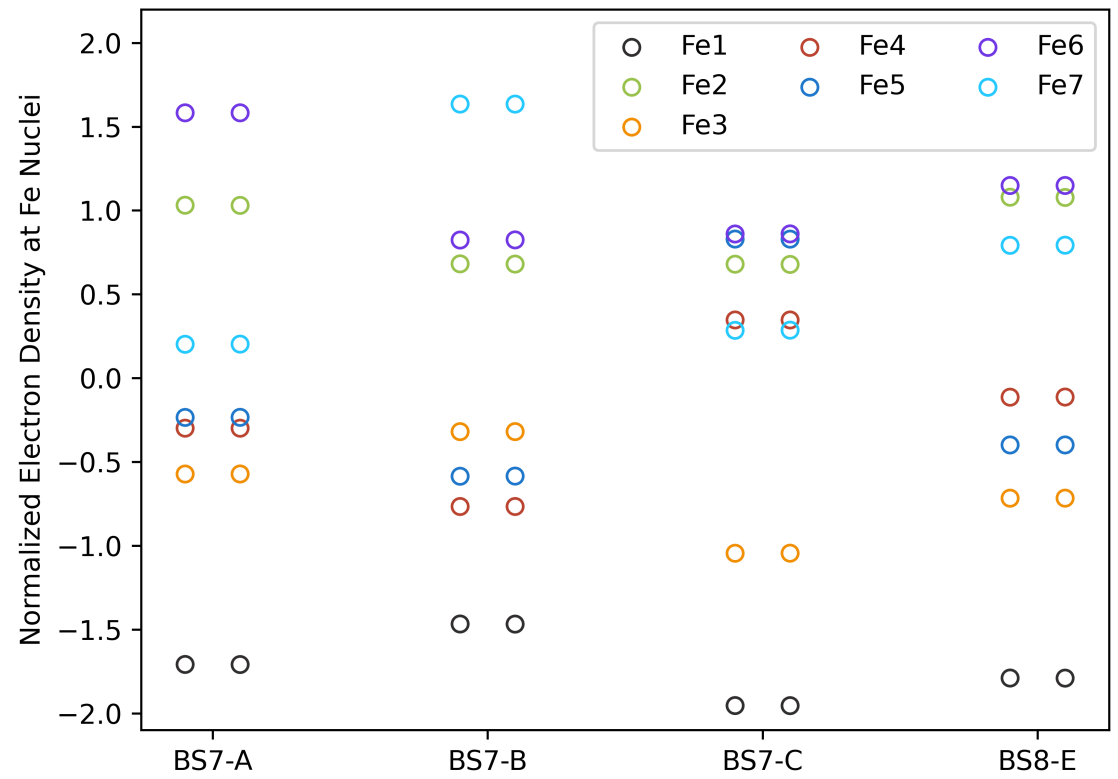

Supplementary Figure 25: **Nucleus electron density sensitivity in FeMo-co.** Electron density values at the iron nuclei in FeMo-co from broken-symmetry DFT calculations, with and without the relativistic picture change. The values are normalized by subtracting the mean and scaled by the standard deviation to show only the iron ordering.

We further checked if details of the relativistic treatment, in particular, the relativistic picture change, can affect the iron ordering. We performed the test on FeMo-co using broken-symmetry DFT. We used the same ORCA input as above while setting the dielectric constant for the implicit solvent to 4.0 to represent the protein environment. The DFT was first converged for high spin ($S_z = 35/2$) and then spin-flipped to generate initial guesses for symmetry-broken solutions targeting $S_z = 3/2$. Calculations were performed on a DFT-optimized geometry previously used for computing Mössbauer isomer shifts (*16*). We focused on a few low-lying spin isomers, namely BS7-A, BS7-B, BS7-C, and BS8-E. Again, the effect of the picture change only introduces a global shift and scaling, as shown in Supplementary Figure 25.

5.5 Mo electron population scale

To establish the scale of the Mo^{3+} electron population, we performed BS-DFT (with the B3LYP functional (*93–95*)), UHF, and UHF/UCCSD for $[MoFe_3S_4(SH)_6]^{3-}$ ($S = 3/2$), which is believed to have the Mo(III) oxidation state (*17, 65*). We use the same basis sets for Mo, Fe, S, and H as those used for the construction of LLDUC model (*9*). UCCSD was performed with core orbitals frozen and using density fitting with an even tempered Gaussian density fitting basis as implemented in PySCF (*85*). The computed Meta-Löwdin populations without Rydberg contributions and the comparison with electron populations in FeMo-co are listed in Supplementary Table 29. We find that at the UCCSD level of theory, the electron populations on Mo in $[MoFe_3S_4(SH)_6]^{3-}$ and the FeMo-co model are very similar.

Supplementary Table 29: The Meta-Löwdin populations without Rydberg contributions at the Fe and Mo sites for $[\mathrm{MoFe_3S_4(SH)_6}]^{3-}$ and BS7-C, BS8-E, and BS8-F states for the FeMo-co (404o, 277e) active space model.

model		theory	Mo	Fe1	Fe2	Fe3	Fe4	Fe5	Fe6	Fe7
$[\mathrm{MoFe_3S_4(SH)_6}]^{3-}$		UKS	40.75	25.04	25.04	24.97				
		UHF	40.44	24.72	24.19	24.66				
		UCCSD	40.60	24.84	24.75	24.73				
FeMo-co	BS7-C	UCCSD	40.58	24.72	24.67	24.75	24.65	24.76	24.81	24.88
	BS8-E		40.58	24.73	24.61	24.71	24.64	24.81	24.79	24.84
	BS8-F		40.58	24.73	24.69	24.63	24.64	24.78	24.79	24.88

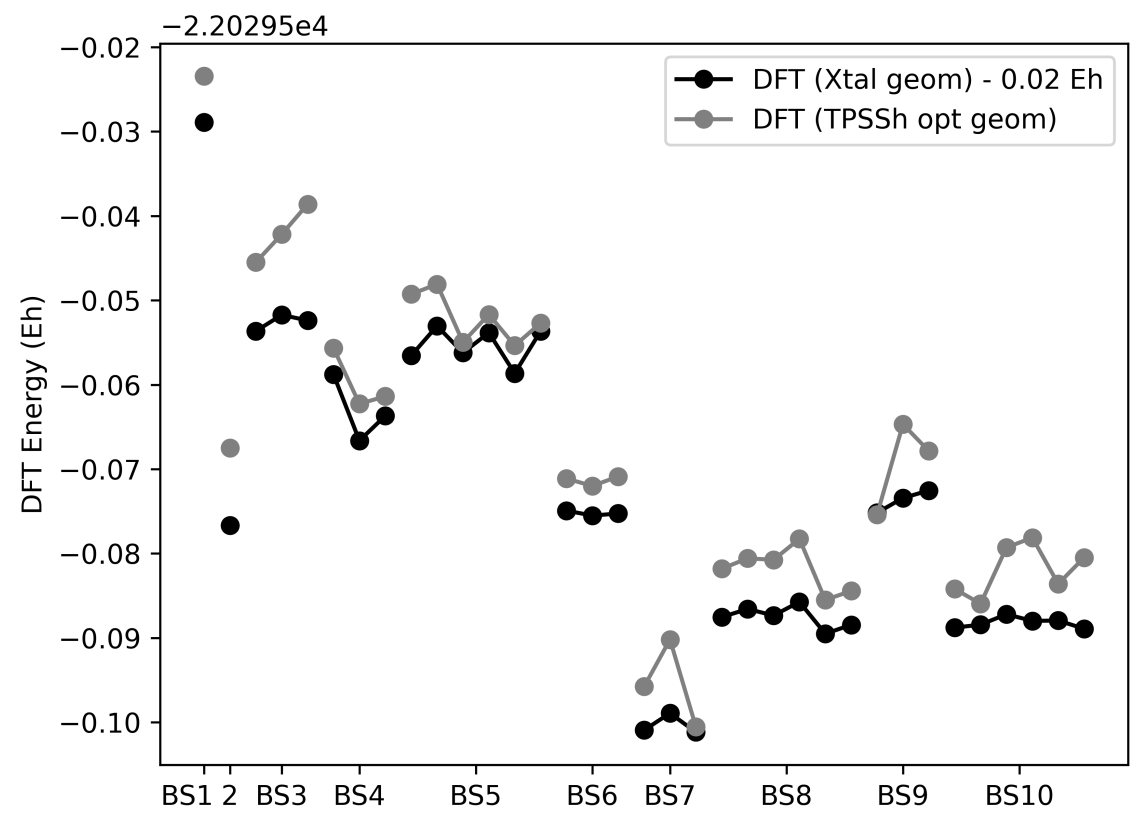

Supplementary Figure 26: **Cluster geometry dependence of BS-DFT energies of spin isomers.**

6 Cluster geometry dependence

The QM/MM MD studies primarily studied the effect of protein fluctuations. We further use broken-symmetry DFT to check the dependence of the energies, iron electron populations, and nucleus electron densities on the FeMo-co cluster geometry. We used the same broken-symmetry DFT protocol as above. Two geometries were considered, a crystal structure cutout (which was used to derive the LLDUC model), and a DFT-optimized geometry (using the TPSSh functional), both taken from Section 4 of the supporting information of Ref. (*16*).

As shown in Supplementary Figure 26, the qualitative energy landscape of spin isomers is consistent between the two geometries, while geometry optimization on the BS7-C (BS7-235) potential energy surface as performed in Ref. (*16*) increases the energy gap between BS7-C and other isomers.

We further examine the iron ordering dependence on geometry, according to the population and nuclear electron density. As shown in Supplementary Figure 27, the general ordering is consistent between the two geometries, particularly in the BS7-C isomer, used for oxidation assignments previously (*16*).

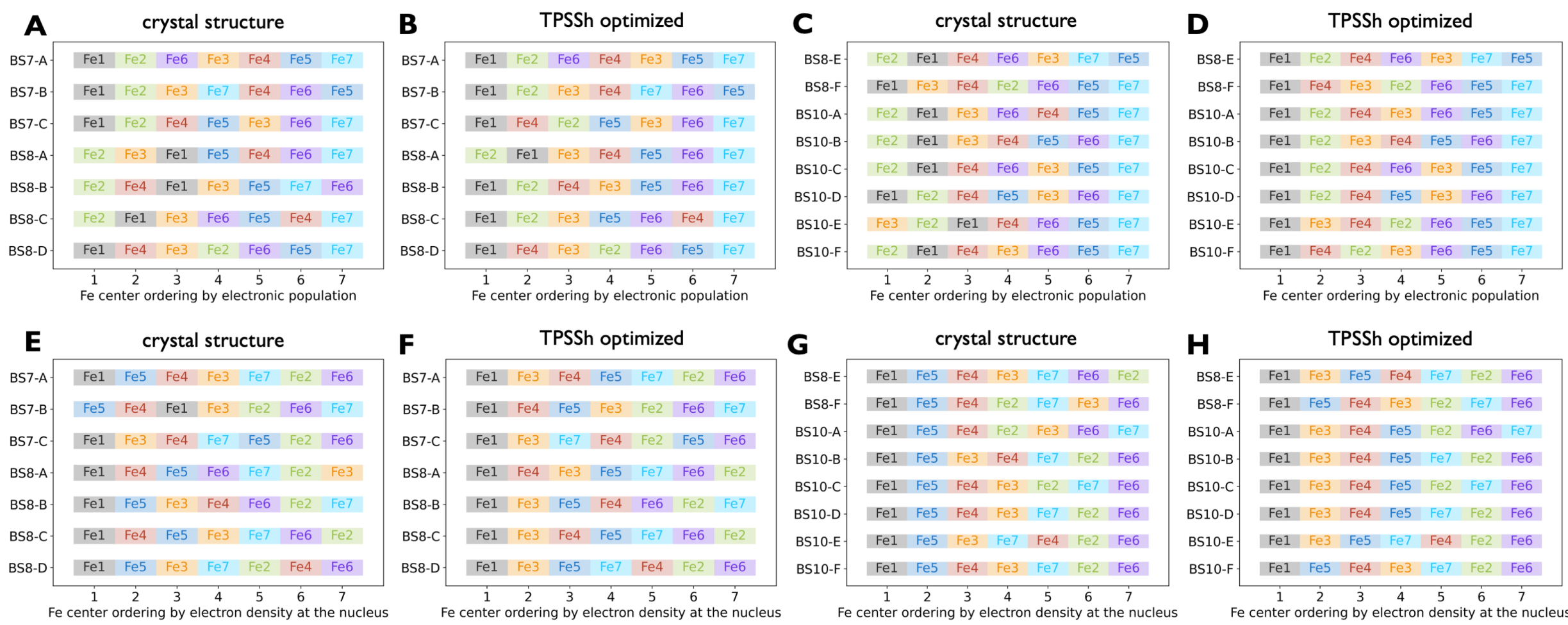

Supplementary Figure 27: **Geometry dependence of the Fe center ordering.** **A,B** Fe center ordering by meta-Löwdin populations for BS7 and BS8-A/B/C/D, computed using the crystal and TPSSh optimized structures, respectively. **C,D** Fe center ordering by meta-Löwdin populations for BS8-E/F and BS10, computed using the crystal and TPSSh optimized structures, respectively. **E,F** Fe center ordering by electron density at the nucleus for BS7 and BS8-A/B/C/D, computed using the crystal and TPSSh optimized structures, respectively. **G,H** Fe center ordering by electron density at the nucleus for BS8-E/F and BS10, computed using the crystal and TPSSh optimized structures, respectively.

7 Model Hamiltonians and qualitative electronic structure

7.1 Model for mixed valence 2Fe-2S clusters

We use a multi-orbital Kanamori model to examine the stabilization of spin states in mixed-valence [2Fe-2S] clusters. The many-body Hamiltonian reads

$$H = \sum_i \sum_\mu \sum_\sigma \varepsilon_{i\mu} n_{i\mu\sigma} + \sum_{i\neq j} \sum_{\mu\nu} \sum_\sigma t_{ij}^{\mu\nu} c_{i\mu\sigma}^\dagger c_{i\nu\sigma}$$
$$+ \sum_i \Big(U \sum_\mu n_{i\mu\uparrow} n_{i\mu\downarrow} + (U-2J) \sum_{\mu<\nu} \sum_{\sigma\sigma'} n_{i\mu\sigma} n_{i\nu\sigma'} - J \sum_{\mu\neq\nu} S_{i\mu}\cdot S_{i\nu} + J \sum_{\mu\neq\nu} c_{i\mu\uparrow}^\dagger c_{i\mu\downarrow}^\dagger c_{i\nu\downarrow} c_{i\nu\uparrow} \Big) , \quad \text{(S8)}$$

where orbital energies ε and hopping integrals t are derived from DFT calculations for a model system taken from Ref. 32, and the Hund's coupling J is set to 0.3 eV. By varying the on-site Coulomb repulsion U from 3 eV to 10 eV, we observe stabilization of all possible spin states, as shown in Supplementary Figure 28. The result for $U = 3$ eV is consistent with the DMRG calculation for a 32-orbital active space model (*32*), which predicts an $S = 1/2$ ground state, however increasing U leads to intermediate- and high-spin ground states.

Typical U values for Fe range from 3 to 6 eV. While double exchange in mixed valence dimers is sometimes used

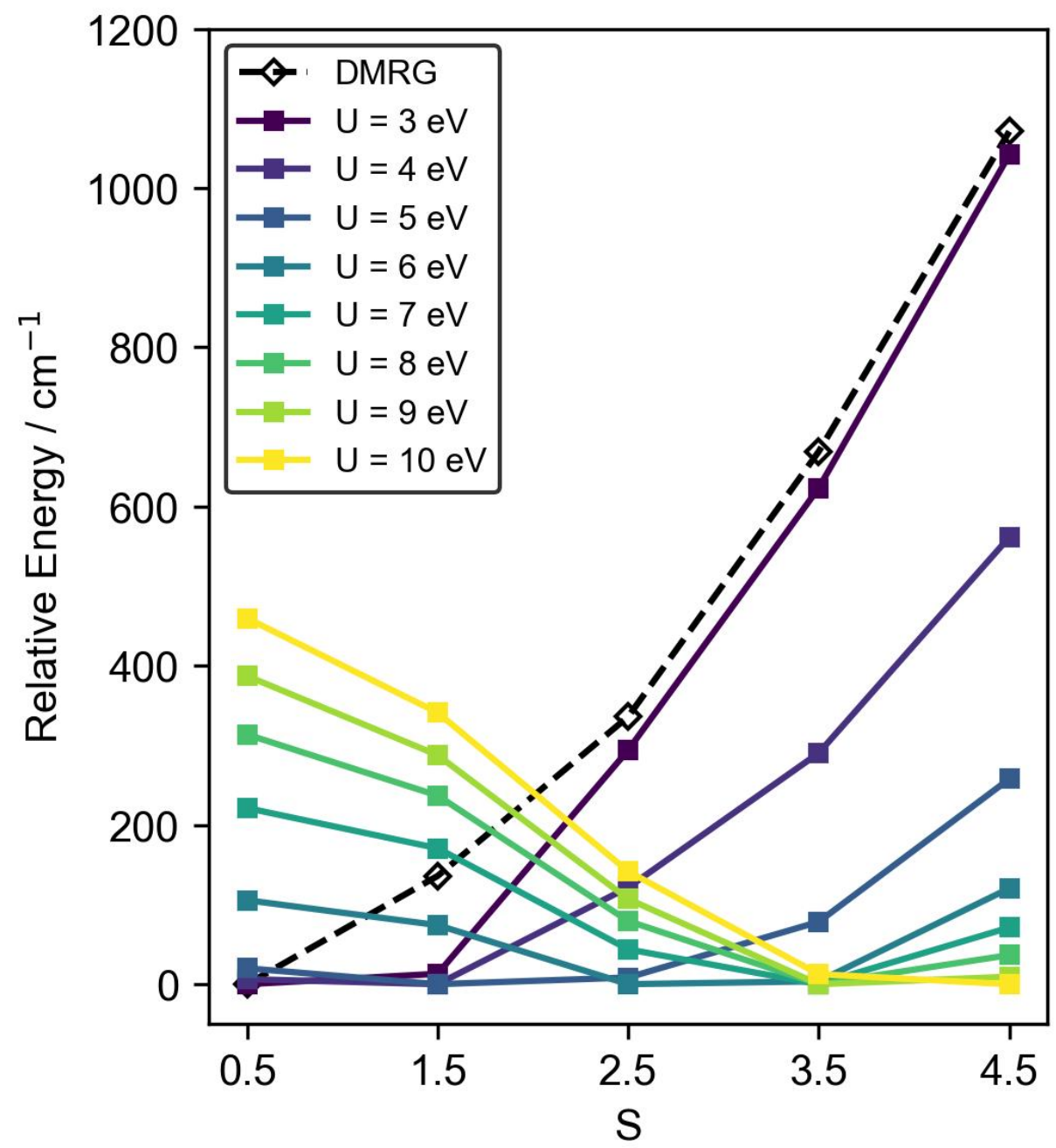

Supplementary Figure 28: **Relative energies of different spin states obtained from a 10-orbital model Hamiltonian for on-site Coulomb repulsion U ranging from 3 eV to 10 eV.** The reference DMRG result is taken from Ref. 32 for a 32-orbital active space.

to suggest a ferromagnetic alignment of neighbouring Fe centers, we see that the ground-state spin configuration is governed by a delicate balance among competing interactions, namely, antiferromagnetic coupling, double exchange, and vibronic coupling, etc. In FeMo-co, the delocalization over multiple Fe centers further complicates a deduction of the relative alignment of neighbouring Fe centers simply from the mixed valence nature.

7.2 Toy Heisenberg model for FeMo-co

7.2.1 Hierarchy of energies in the Heisenberg-like models

We first briefly recall the theoretical terminology used to describe different excitations and the theoretical basis of spontaneous symmetry breaking in Heisenberg-like models, as first elucidated by Anderson's treatment of the antiferromagnetic state (*48, 104*).

It is known that the ground-states of spin systems on certain lattices can have long-range spin-order and that such ground-states can be described by a spontaneously symmetry broken state that is not an eigenstate of S^2. The basis for this was first described by Anderson. In such systems, there is a set of low-lying excitations whose energy spacing goes like $1/V$, where V is the volume of the system. As $V \to \infty$, this gap vanishes, and it is therefore valid to consider a symmetry broken eigenstate that is a mixture of such excitations. To give rise to a long-range spin order

(such as antiferromagnetic long-range order) the set of states that are mixed together must have the same correlations as $V \to \infty$. This set of states is known as the 'tower of states' and the symmetry broken ground-state lies in the manifold of this tower of states.

There is another set of excitations in the spin system that is associated with the Goldstone mode, or spin-wave, excitations. These are also gapless in a system with spontaneous symmetry breaking, but they can be distinguished from the above tower of states because the gap scales like $1/L$, the linear dimension of the problem. These excitations are referred to as magnons.

As discussed in Ref. (*48*), the magnon excitations modify the order of the ground-state. They are the excitations that are measured in spectroscopy. The tower of states can be better thought of as a degeneracy of the ground-state. By mixing the tower of states to obtain a spontaneously symmetry broken state, one obtains a physically realistic ground-state.

In this language, in FeMo-co the manifold of spin-pure eigenstates that mix to form a given broken symmetry isomer is the 'tower of states', while the gap between spin isomer energies is analogous to (one or more) magnon excitations.

7.2.2 Numerical simulations

Hamiltonian. To achieve a better qualitative understanding of the spin coupling in FeMo-co and the consequences of the broken-symmetry treatment, we consider a toy model for FeMo-co with a Heisenberg type interaction

$$\hat{H} = \sum_{\langle ij \rangle}^{n} J_{ij}\, \hat{S}_i \cdot \hat{S}_j + E_0 \tag{S9}$$

where E_0 is an energy constant (for fitting purposes), $i, j = 1, 2, \cdots, 7$ representing 7 Fe sites (all with a local spin $S = 2$) , and $j = 8$ representing 1 Mo site (with a local spin $S = \frac{1}{2}$). In this setup, the charge state is different from the E_0 state, and charge fluctuations and electron hopping for Fe sites are ignored. However, the use of a spin model allows the exact solutions to be obtained by simple diagonalization, and this is sufficient to illustrate the hierarchy of states in the model. We further assume a topology with C_3 symmetry, with the six independent J_{ij} parameters listed in Supplementary Table 30.

Parameter fitting. We determine the J_{ij} and E_0 parameters using least squares fitting of the UCCSD(T) energies (and alternately UHF or UCCSD energies) computed in the LLDUC model for the 35 spin isomers. To compute the Heisenberg model Hamiltonian energy for fitting purposes, we assume that, regardless of the level of theory (i.e. UHF or UCC), every BS state corresponds to a pure Ising state. This means, we set $\langle \hat{S}_i \cdot \hat{S}_j \rangle = 4.0$ and -6.0 for FM and AFM Fe–Fe coupling respectively, and $\langle \hat{S}_i \cdot \hat{S}_j \rangle = 1.0$ and -1.5 for FM and AFM Fe–Mo coupling

Supplementary Table 30: The independent J_{ij} parameters used in the toy Heisenberg model for FeMo-co.

type	notation	(i, j) pairs					
left terminal	J_{LT}	(1,2)	(1,3)	(1,4)			
left cubane	J_{LC}	(2,3)	(2,4)	(3,4)			
right cubane	J_{RC}	(5,6)	(5,7)	(6,7)			
right (Mo) terminal	J_{RT}	(5,8)	(6,8)	(7,8)			
central (axial)	J_{CA}	(2,6)	(3,7)	(4,5)			
central (cross)	J_{CC}	(2,5)	(2,7)	(3,5)	(3,6)	(4,6)	(4,7)

respectively. The FM or AFM coupling is determined using the 1- and 2-particle density matrices of the UHF states. We list the Heisenberg model parameters determined using different levels of theory in Supplementary Table 31. The energy fitting error is shown in Supplementary Figure 29. The small energy fluctuation within each BS family for the Heisenberg model is due to the Fe–Mo coupling sign mismatch between the BS initial guess and the actual UHF states (this only appears in a few spin isomers). Since the Heisenberg model assumes C_3 symmetry, the fitting error mostly measures the variation of the LLDUC model energies within each of the BS families.

Supplementary Table 31: The Heisenberg model J_{ij} (in cm^{-1}) and E_0 (in mHa, shifted by -22140 Hartrees) parameters determined using the UHF, UCCSD, or UCCSD(T) energies computed in the LLDUC model. Root mean square error (RMSE) of the fitting is given in mHa.

theory	J_{LT}	J_{LC}	J_{RC}	J_{RT}	J_{CA}	J_{CC}	E_0	RMSE
UHF	219.03	304.86	54.29	247.49	77.91	13.43	41.83	24.52
UCCSD	564.78	538.70	-14.90	135.02	185.82	48.45	-242.23	44.99
UCCSD(T)	581.95	586.48	32.16	117.97	168.21	23.91	-295.32	37.01

Ground and excited states. In the following, we focus on solving for the ground and excited states of the model with parameters fitted to UCCSD(T) energies. In Supplementary Table 32 we list the energies of the lowest eight eigenstates found using state-averaged spin-adapted DMRG (with maximal bond dimension $D = 800$, which produces the exact result for this problem) for $S = 1/2, 3/2, \cdots, 13/2$, respectively. The ground state energy for the model is -22140.335969 Hartrees, with $S = 3/2$. The $S = 1/2$ and $S = 5/2$ states are found to be 0.63 and 0.39 mHa higher in energy, respectively, indicating a small spin gap in the model. Note that as the Heisenberg model does not correspond to the same charge state as the E0 state, the spin of the ground-state here does not imply anything about the true ground-state spin of FeMo-co in the LLDUC model.

Approximate solutions. Because the gap between different pure S eigenstates is small, then in an approximate MPS (which is not spin-adapted), different S eigenstates will mix for a given S_z state. This mixing is expected to persist until we reach a bond dimension compatible with resolving the spin gap. Thus we study the deviation of $\langle S^2 \rangle$ from $S(S+1)$ in approximate broken-symmetry solutions using non-spin-adapted DMRG with a series of low MPS bond dimensions. The results are shown in Supplementary Figure 30. We find that the amount of spin contamination

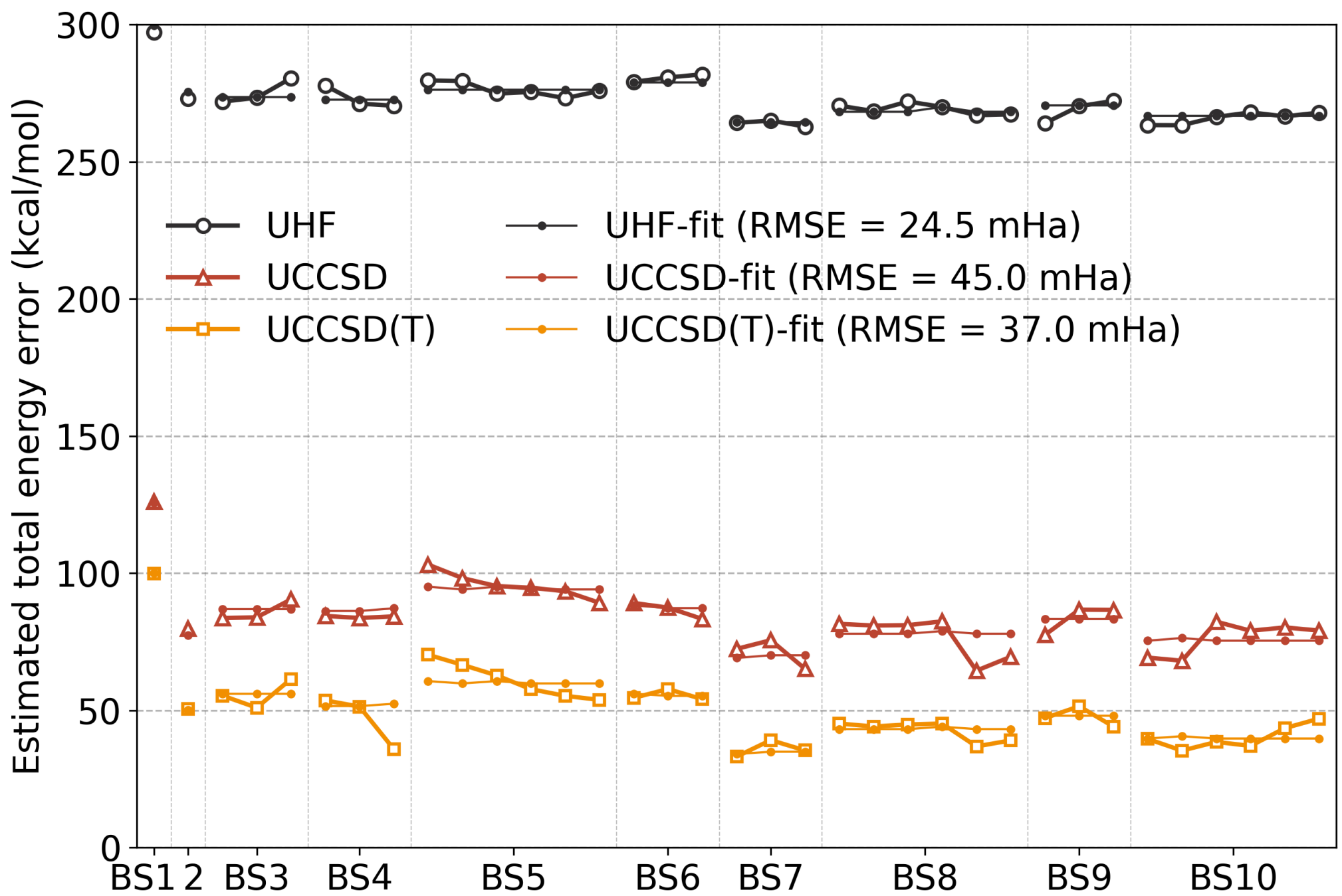

Supplementary Figure 29: **Comparison between the predicted and the actual energies, for the Heisenberg model fitted using UHF, UCCSD, and UCCSD(T) energies.**

Supplementary Table 32: The Heisenberg model eigenstate energies (in mHa, relative to the $S = 3/2$ ground state energy -22140.335969 Hartrees) determined using state-averaged spin-adapted DMRG.

S	eigenstate energies							
1/2	0.626	0.626	0.943	1.244	1.244	1.362	1.630	1.630
3/2	0.000	0.246	0.246	0.747	1.058	1.058	1.105	1.432
5/2	0.389	0.389	0.892	1.193	1.301	1.345	1.345	1.528
7/2	1.220	1.220	1.292	2.238	2.238	2.290	2.290	2.726
9/2	2.500	2.666	2.775	2.775	3.588	3.588	3.675	4.421
11/2	4.818	4.818	5.198	5.198	5.249	5.338	5.536	5.654
13/2	8.046	8.046	8.481	8.481	8.542	9.025	9.025	9.115

Supplementary Table 33: The energy and $\langle S_z \rangle$ expectation at Fe and Mo sites of the low-energy Heisenberg model eigenstates determined using non-spin-adapted DMRG. Energies are in mHa relative to the $S = 3/2$ ground state energy.

(S, S_z)	energy	Fe1	Fe2	Fe3	Fe4	Fe5	Fe6	Fe7	Mo8
(3/2,1/2)	0.000	0.44	-0.15	-0.15	-0.15	0.20	0.20	0.20	-0.10
(3/2,1/2)	0.246	0.40	-0.07	-0.31	-0.01	0.05	0.13	0.41	-0.10
(3/2,1/2)	0.246	0.40	-0.19	0.05	-0.25	0.34	0.26	-0.02	-0.10
(3/2,3/2)	0.000	1.32	-0.44	-0.44	-0.44	0.60	0.60	0.60	-0.30
(3/2,3/2)	0.246	1.21	0.16	-0.71	-0.60	0.84	-0.07	0.98	-0.30
(3/2,3/2)	0.246	1.21	-0.93	-0.05	-0.17	0.32	1.23	0.19	-0.30

(measured by the difference between $\langle S^2 \rangle$ and $S(S+1)$) decreases rapidly once the energy error is close to or smaller than the spin gap, which is 0.389 mHa in this model. When the MPS bond dimension is large enough, the pure-spin is recovered for the ground state.

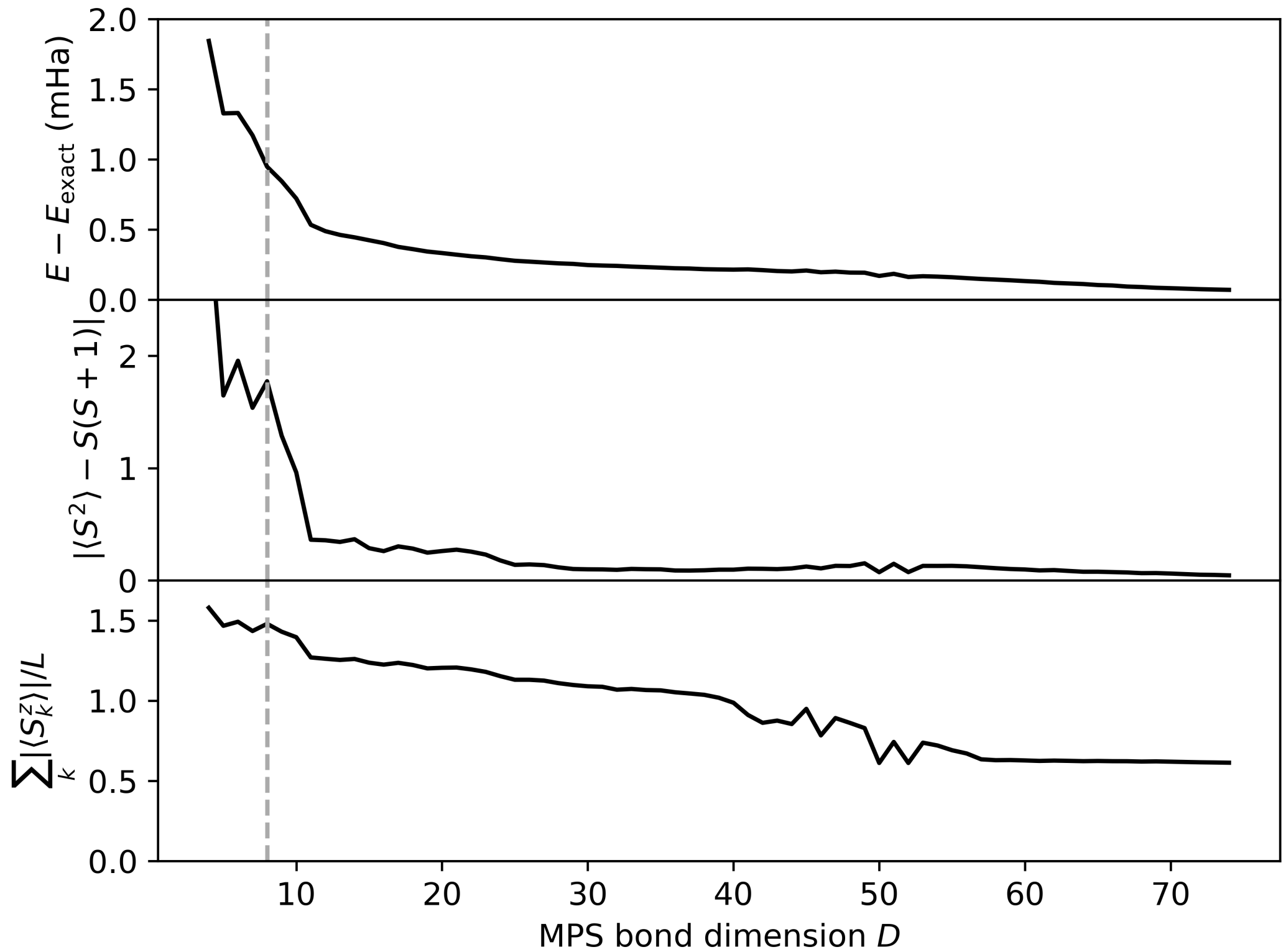

Supplementary Figure 30: **The energy error, difference between $\langle S^2 \rangle$ and $S(S+1)$ (with $S = 3/2$), and average $\langle S_z \rangle$ on each site for the Heisenberg model ground state computed using non-spin-adapted DMRG at finite MPS bond dimensions.** The dashed gray line indicates the MPS bond dimension with 1 mHa energy error.

The nature of spin isomers. Based on the eigenstates for this Heisenberg model, we now briefly discuss the nature of spin isomers in FeMo-co models. Supplementary Figure 31 shows the spin correlation computed for the low-energy states, using exact spin-adapted DMRG (SA-DMRG) and approximate non-spin-adapted DMRG (NSA-DMRG, with small MPS bond dimension). When we use approximate NSA-DMRG, two types of symmetry breaking appear: (i) total spin symmetry, as can be seen by the non-half-integer $\langle S \rangle$ values; and (ii) C_3 spatial symmetry, as the energy of the C_3-equivalent BS8-A, BS8-D, and BS8-E states are no longer degenerate. The origin of (i) is the fact that in an approximate non-spin-adapted theory, states with different S, but with otherwise similar correlations, mix to overcome the representation limitation of the ansatz. The origin of (ii) is the one-dimensional asymmetric ordering of C_3-equivalent sites in DMRG (but note that in FeMo-co this C_3 symmetry is actually broken anyway).

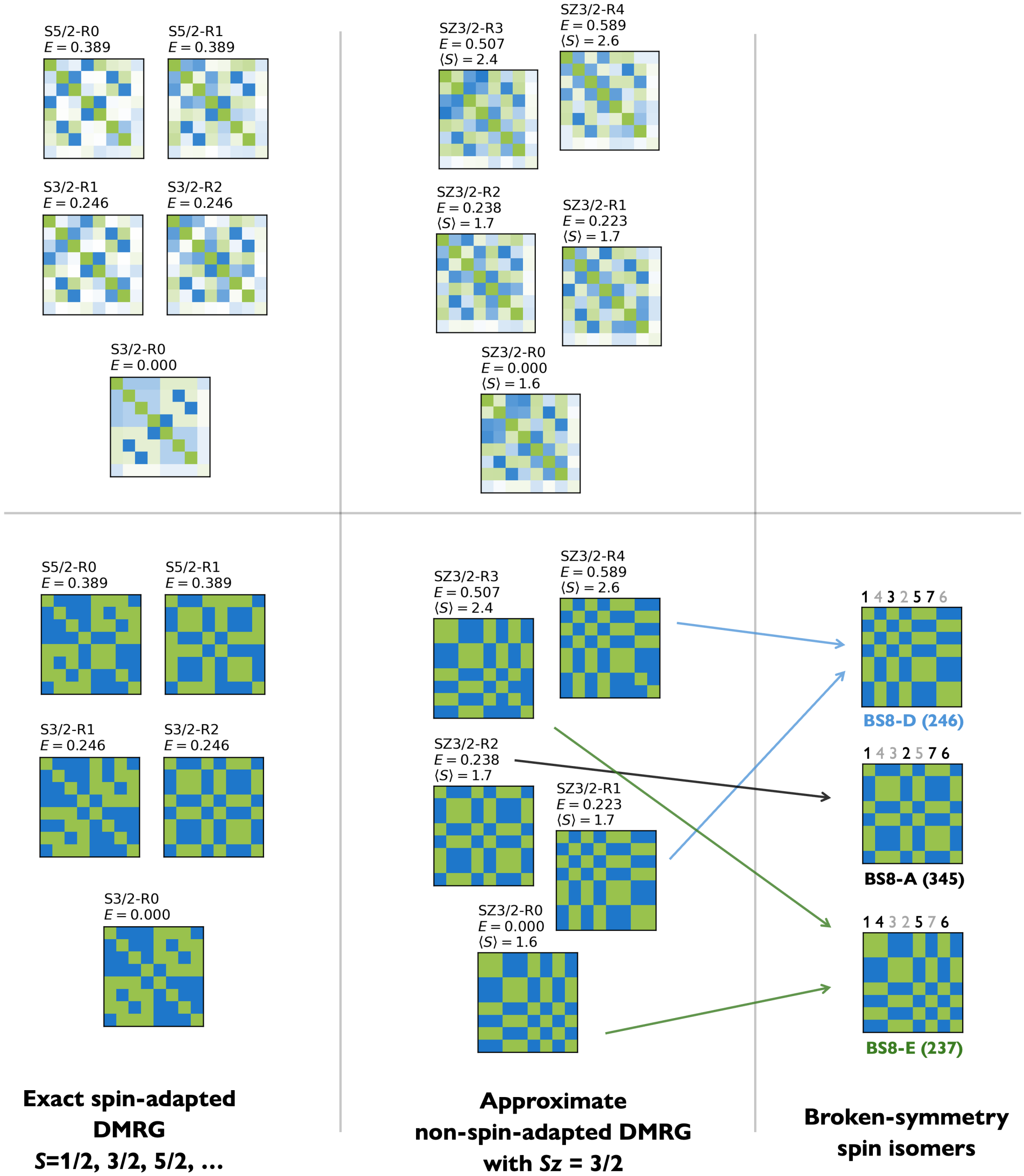

Supplementary Figure 31: **Spin correlation (upper panel) and its sign (lower panel) between Fe and Mo sites in low-energy states computed using exact spin-adapted DMRG and exact and approximate non-spin-adapted DMRG.** Site ordering in spin correlation plots is the computational ordering: Fe1-Fe4-Fe3-Fe2-Fe5-Fe7-Fe6-Mo8, to clearly show the one-dimensional entanglement structure. The ith eigenstate (counting from zero) of $S = 3/2$ and $S_z = 3/2$ states are denoted as "S3/2-Ri" and "SZ3/2-Ri", respectively. Energies are in millihartrees, relative to the ground state.

The relationship between the correlations in the spin-adapted eigenstates, the approximate non-spin-adapted eigenstates, and the mean-field broken symmetry spin isomers can be seen from the correlation functions and the colors. While the correlations are slightly hard to interpret in the pure spin eigenstates due to mixing from the exact C_3 symmetry, one can see some of the BS8 correlations already (e.g. BS8A). These become very clear in the non-spin-adapted approximate eigenstates with fixed S_z, which are then clearly connected to the mean-field spin isomers. Thus the approximate MPS calculations of spin isomers can be viewed as representing a “tower” of pure spin eigenstates with a similar spin coupling pattern, in a small energy window, analogous to the theoretical structure of states in Heisenberg models originally proposed by Anderson (*48*).

REFERENCES

1. H. Beinert, R. H. Holm, E. Münck, Iron-sulfur clusters: Nature's modular, multipurpose structures. *Science* **277**, 653–659 (1997).

2. K. Tanifuji, Y. Ohki, Metal–sulfur compounds in N_2 reduction and nitrogenase-related chemistry. *Chem. Rev.* **120**, 5194–5251 (2020).

3. J. Kim, D. C. Rees, Crystallographic structure and functional implications of the nitrogenase molybdenum–iron protein from *Azotobacter vinelandii*. *Nature* **360**, 553–560 (1992).

4. O. Einsle, F. A. Tezcan, S. L. A. Andrade, B. Schmid, M. Yoshida, J. B. Howard, D. C. Rees, Nitrogenase MoFe-protein at 1.16 Å resolution: A central ligand in the FeMo-cofactor. *Science* **297**, 1696–1700 (2002).

5. H.-I. Lee, P. M. C. Benton, M. Laryukhin, R. Y. Igarashi, D. R. Dean, L. C. Seefeldt, B. M. Hoffman, The interstitial atom of the nitrogenase FeMo-cofactor: ENDOR and ESEEM show it is not an exchangeable nitrogen. *J. Am. Chem. Soc.* **125**, 5604–5605 (2003).

6. T. Spatzal, M. Aksoyoglu, L. Zhang, S. L. A. Andrade, E. Schleicher, S. Weber, D. C. Rees, O. Einsle, Evidence for interstitial carbon in nitrogenase FeMo cofactor. *Science* **334**, 940–940 (2011).

7. K. M. Lancaster, M. Roemelt, P. Ettenhuber, Y. Hu, M. W. Ribbe, F. Neese, U. Bergmann, S. DeBeer, X-ray emission spectroscopy evidences a central carbon in the nitrogenase iron-molybdenum cofactor. *Science* **334**, 974–977 (2011).

8. M. Reiher, N. Wiebe, K. M. Svore, D. Wecker, M. Troyer, Elucidating reaction mechanisms on quantum computers. *Proc. Natl. Acad. Sci. U.S.A.* **114**, 7555–7560 (2017).

9. Z. Li, J. Li, N. S. Dattani, C. J. Umrigar, G. K.-L. Chan, The electronic complexity of the ground-state of the FeMo cofactor of nitrogenase as relevant to quantum simulations. *J. Chem. Phys.* **150**, 024302 (2019).

10. K. Wan, M. Berta, E. T. Campbell, Randomized quantum algorithm for statistical phase estimation. *Phys. Rev. Lett.* **129**, 030503 (2022).

11. D. W. Berry, C. Gidney, M. Motta, J. R. McClean, R. Babbush, Qubitization of arbitrary basis quantum chemistry leveraging sparsity and low rank factorization. *Quantum* **3**, 208 (2019).

12. M. Luo, J. I. Cirac, Efficient simulation of quantum chemistry problems in an enlarged basis set. *PRX Quantum* **6**, 010355 (2025).

13. G. H. Low, R. King, D. W. Berry, Q. Han, A. E. DePrince, A. F. White, R. Babbush, R. D. Somma, N. C. Rubin, Fast quantum simulation of electronic structure by spectral amplification. *Phys. Rev. X* **15**, 041016 (2025).

14. C. Van Stappen, L. Decamps, G. E. Cutsail III, R. Bjornsson, J. T. Henthorn, J. A. Birrell, S. De Beer, The spectroscopy of nitrogenases. *Chem. Rev.* **120**, 5005–5081 (2020).

15. E. Münck, H. Rhodes, W. H. Orme-Johnson, L. C. Davis, W. J. Brill, V. K. Shah, Nitrogenase. VIII. Mössbauer and EPR spectroscopy. The MoFe protein component from *Azotobacter vinelandii* OP. *Biochim. Biophys. Acta* **400**, 32–53 (1975).

16. R. Björnsson, F. Neese, S. DeBeer, Revisiting the mössbauer isomer shifts of the FeMoco cluster of nitrogenase and the cofactor charge. *Inorg. Chem.* **56**, 1470–1477 (2017).

17. R. Björnsson, F. A. Lima, T. Spatzal, T. Weyhermüller, P. Glatzel, E. Bill, O. Einsle, F. Neese, S. De Beer, Identification of a spin-coupled Mo(III) in the nitrogenase iron–molybdenum cofactor. *Chem. Sci.* **5**, 3096–3103 (2014).

18. J. K. Kowalska, J. T. Henthorn, C. van Stappen, C. Trncik, O. Einsle, D. Keavney, S. DeBeer, X-ray magnetic circular dichroism spectroscopy applied to nitrogenase and related models: Experimental evidence for a spin-coupled molybdenum (III) center. *Angew. Chem. Int. Ed. Engl.* **58**, 9373–9377 (2019).

19. H. E. Stanley, T. A. Kaplan, High-temperature expansions-the classical Heisenberg model. *Phys. Rev. Lett.* **16**, 981–983 (1966).

20. T. Lovell, R. A. Torres, W. G. Han, T. Liu, D. A. Case, L. Noodleman, Metal substitution in the active site of nitrogenase MFe7S9 (M= Mo^{4+}, V^{3+}, Fe^{3+}). *Inorg. Chem.* **41**, 5744–5753 (2002).

21. L. Cao, U. Ryde, Influence of the protein and DFT method on the broken-symmetry and spin states in nitrogenase. *Int. J. Quantum Chem.* **118**, e25627 (2018).

22. Z. Li, G. K.-L. Chan, Spin-projected matrix product states: Versatile tool for strongly correlated systems. *J. Chem. Theory Comput.* **13**, 2681–2695 (2017).

23. Z. Li, S. Guo, Q. Sun, G. K.-L. Chan, Electronic landscape of the P-cluster of nitrogenase as revealed through many-electron quantum wavefunction simulations. *Nat. Chem.* **11**, 1026–1033 (2019).

24. S. Lee, J. Lee, H. Zhai, Y. Tong, A. M. Dalzell, A. Kumar, P. Helms, J. Gray, Z. H. Cui, W. Liu, M. Kastoryano, R. Babbush, J. Preskill, D. R. Reichman, E. T. Campbell, E. F. Valeev, L. Lin, G. K. L. Chan, Evaluating the evidence for exponential quantum advantage in ground-state quantum chemistry. *Nat. Commun.* **14**, 1952 (2023).

25. B. Benediktsson, R. Björnsson, QM/MM study of the nitrogenase MoFe protein resting state: Broken-symmetry states, protonation states, and QM region convergence in the FeMoco active site. *Inorg. Chem.* **56**, 13417–13429 (2017).

26. L. Cao, U. Ryde, Extremely large differences in DFT energies for nitrogenase models. *Phys. Chem. Chem. Phys.* **21**, 2480–2488 (2019).

27. S. J. Yoo, H. C. Angove, V. Papaefthymiou, B. K. Burgess, E. Münck, Mössbauer study of the MoFe protein of nitrogenase from *Azotobacter vinelandii* using selective ^{57}Fe enrichment of the M-centers. *J. Am. Chem. Soc.* **122**, 4926–4936 (2000).

28. E. D. Badding, S. Srisantitham, D. A. Lukoyanov, B. M. Hoffman, D. L. M. Suess, Connecting the geometric and electronic structures of the nitrogenase iron–molybdenum cofactor through site-selective ^{57}Fe labelling. *Nat. Chem.* **15**, 658–665 (2023).

29. J. T. Henthorn, R. J. Arias, S. Koroidov, T. Kroll, D. Sokaras, U. Bergmann, D. C. Rees, S. DeBeer, Localized electronic structure of nitrogenase FeMoco revealed by selenium K-edge high resolution x-ray absorption spectroscopy. *J. Am. Chem. Soc.* **141**, 13676–13688 (2019).

30. T. Spatzal, J. Schlesier, E. M. Burger, D. Sippel, L. Zhang, S. L. A. Andrade, D. C. Rees, O. Einsle, Nitrogenase FeMoco investigated by spatially resolved anomalous dispersion refinement. *Nat. Commun.* **7**, 10902 (2016).

31. Z. Li, Entanglement-minimized orbitals enable faster quantum simulation of molecules. *Phys. Rev. Lett.* **135**, 210601 (2025).

32. S. Sharma, K. Sivalingam, F. Neese, G. K.-L. Chan, Low-energy spectrum of iron–sulfur clusters directly from many-particle quantum mechanics. *Nat. Chem.* **6**, 927–933 (2014).

33. J. M. Montgomery, D. A. Mazziotti, Strong electron correlation in nitrogenase cofactor, FeMoco. *J. Phys. Chem. A* **122**, 4988–4996 (2018).

34. Z. Li, Expressibility of comb tensor network states (CTNS) for the P-cluster and the FeMo-cofactor of nitrogenase. *Electron. Struct.* **3**, 014001 (2021).

35. C. Xiang, W. Jia, W.-H. Fang, Z. Li, Distributed multi-GPU ab initio density matrix renormalization group algorithm with applications to the P-cluster of nitrogenase. *J. Chem. Theory Comput.* **20**, 775–786 (2024).

36. J. Brabec, J. Brandejs, K. Kowalski, S. Xantheas, Ö. Legeza, L. Veis, Massively parallel quantum chemical density matrix renormalization group method. *J. Comput. Chem.* **42**, 534–544 (2021).

37. A. Menczer, M. van Damme, A. Rask, L. Huntington, J. Hammond, S. S. Xantheas, M. Ganahl, Ö. Legeza, Parallel implementation of the Density Matrix Renormalization Group method achieving a quarter petaFLOPS performance on a single DGX-H100 GPU node. *J. Chem. Theory Comput.* **20**, 8397–8404 (2024).

38. S. R. White, Density matrix formulation for quantum renormalization groups. *Phys. Rev. Lett.* **69**, 2863–2866 (1992).

39. S. R. White, Density-matrix algorithms for quantum renormalization groups. *Phys. Rev. B* **48**, 10345–10356 (1993).

40. G. K.-L. Chan, M. Head-Gordon, Highly correlated calculations with a polynomial cost algorithm: A study of the density matrix renormalization group. *J. Chem. Phys.* **116**, 4462–4476 (2002).

41. A. O. Mitrushenkov, G. Fano, F. Ortolani, R. Linguerri, P. Palmieri, Quantum chemistry using the density matrix renormalization group. *J. Chem. Phys.* **115**, 6815–6821 (2001).

42. S. Wouters, D. Van Neck, The density matrix renormalization group for ab initio quantum chemistry. *Eur. Phys. J. D* **68**, 272 (2014).

43. S. Keller, M. Dolfi, M. Troyer, M. Reiher, An efficient matrix product operator representation of the quantum chemical Hamiltonian. *J. Chem. Phys.* **143**, 244118 (2015).

44. A. Baiardi, M. Reiher, The density matrix renormalization group in chemistry and molecular physics: Recent developments and new challenges. *J. Chem. Phys.* **152**, 040903 (2020).

45. F. Verstraete, T. Nishino, U. Schollwöck, M. C. Bañuls, G. K. Chan, M. E. Stoudenmire, Density matrix renormalization group, 30 years on. *Nat. Rev. Phys.* **5**, 273–276 (2023).

46. R. J. Bartlett, M. Musiał, Coupled-cluster theory in quantum chemistry. *Rev. Mod. Phys.* **79**, 291–352 (2007).

47. I. Shavitt, R. J. Bartlett, *Many-body Methods in Chemistry and Physics: MBPT and Coupled-cluster Theory* (Cambridge Univ. Press, 2010).

48. H. Tasaki, Long-range order, “Tower” of states, and symmetry breaking in lattice quantum systems. *J. Stat. Phys.* **174**, 735–761 (2019).

49. L. Noodleman, C. Y. Peng, D. A. Case, J.-M. Mouesca, Orbital interactions, electron delocalization and spin coupling in iron-sulfur clusters. *Coord. Chem. Rev.* **144**, 199–244 (1995).

50. C. Li, G. K.-L. Chan, Accurate QM/MM molecular dynamics for periodic systems in GPU4PySCF with applications to enzyme catalysis. *J. Chem. Theory Comput.* **21**, 803–816 (2025).

51. J. A. Pople, J. S. Binkley, R. Seeger, Theoretical models incorporating electron correlation. *Int. J. Quantum Chem.* **10**, 1–19 (1976).

52. L. Cao, O. Caldararu, U. Ryde, Protonation and reduction of the FeMo cluster in nitrogenase studied by quantum mechanics/molecular mechanics (QM/MM) calculations. *J. Chem. Theory Comput.* **14**, 6653–6678 (2018).

53. C. Li, G. K.-L. Chan, Predictive free energy simulations through hierarchical distillation of quantum Hamiltonians. *Proc. Natl. Acad. Sci. U.S.A.* **123**, e2529120123 (2026).

54. H. Zhai, G. K.-L. Chan, Low communication high performance ab initio density matrix renormalization group algorithms. *J. Chem. Phys.* **154**, 224116 (2021).

55. H. Zhai, H. R. Larsson, S. Lee, Z. H. Cui, T. Zhu, C. Sun, L. Peng, R. Peng, K. Liao, J. Tölle, J. Yang, S. Li, G. K.-L. Chan, BLOCK2: A comprehensive open source framework to develop and apply state-of-the-art DMRG algorithms in electronic structure and beyond. *J. Chem. Phys.* **159**, 234801 (2023).

56. Q. Sun, X. Zhang, S. Banerjee, P. Bao, M. Barbry, N. S. Blunt, N. A. Bogdanov, G. H. Booth, J. Chen, Z. H. Cui, J. J. Eriksen, Y. Gao, S. Guo, J. Hermann, M. R. Hermes, K. Koh, P. Koval, S. Lehtola, Z. Li, J. Liu, N. Mardirossian, J. D. McClain, M. Motta, B. Mussard, H. Q. Pham, A. Pulkin, W. Purwanto, P. J. Robinson, E. Ronca, E. R. Sayfutyarova, M. Scheurer, H. F. Schurkus, J. E. T. Smith, C. Sun, S. N. Sun, S. Upadhyay, L. K. Wagner, X. Wang, A. White, J. D. Whitfield, M. J. Williamson, S. Wouters, J. Yang, J. M. Yu, T. Zhu, T. C. Berkelbach, S. Sharma, A. Y. Sokolov, G. K.-L. Chan, Recent developments in the PySCF program package. *J. Chem. Phys.* **153**, 024109 (2020).

57. S. Sharma, G. K.-L. Chan, Spin-adapted density matrix renormalization group algorithms for quantum chemistry. *J. Chem. Phys.* **136**, 124121 (2012).

58. P. Eastman, R. Galvelis, R. P. Peláez, C. R. A. Abreu, S. E. Farr, E. Gallicchio, A. Gorenko, M. M. Henry, F. Hu, J. Huang, A. Krämer, J. Michel, J. A. Mitchell, V. S. Pande, J. P. G. L. M. Rodrigues, J. Rodriguez-Guerra, A. C. Simmonett, S. Singh, J. Swails, P. Turner, Y. Wang,

I. Zhang, J. D. Chodera, G. de Fabritiis, T. E. Markland, OpenMM 8: Molecular dynamics simulation with machine learning potentials. *J. Phys. Chem. B* **128**, 109–116 (2024).

59. C. Bannwarth, S. Ehlert, S. Grimme, GFN2-xTB—An accurate and broadly parametrized self-consistent tight-binding quantum chemical method with multipole electrostatics and density-dependent dispersion contributions. *J. Chem. Theory Comput.* **15**, 1652–1671 (2019).

60. R. Björnsson, "ASH: A Python-based multiscale modelling program," in *12th Triennial Congress of the World Association of Theoretical and Computational Chemists* (*WATOC 2020*) (WATOC, 2022).

61. A. K. Rappé, C. J. Casewit, K. S. Colwell, W. A. Goddard III, W. M. Skiff, UFF, a full periodic table force field for molecular mechanics and molecular dynamics simulations. *J. Am. Chem. Soc.* **114**, 10024–10035 (1992).

62. J. A. Maier, C. Martinez, K. Kasavajhala, L. Wickstrom, K. E. Hauser, C. Simmerling, ff14SB: Improving the accuracy of protein side chain and backbone parameters from ff99SB. *J. Chem. Theory Comput.* **11**, 3696–3713 (2015).

63. L.-P. Wang, T. J. Martinez, V. S. Pande, Building force fields: An automatic, systematic, and reproducible approach. *J. Phys. Chem. Lett.* **5**, 1885–1891 (2014).

64. Q. Sun, G. K.-L. Chan, Exact and optimal quantum mechanics/molecular mechanics boundaries. *J. Chem. Theory Comput.* **10**, 3784–3790 (2014).

65. M. Cook, M. Karplus, Electronic structure of the $[MoFe_3S_4(SH)_6]^{3-}$ ion: A broken-symmetry metal–sulfur cluster. *J. Chem. Phys.* **83**, 6344–6366 (1985).

66. T. Lovell, J. Li, T. Liu, D. A. Case, L. Noodleman, FeMo cofactor of nitrogenase: A density functional study of states MN, MOX, MR, and MI. *J. Am. Chem. Soc.* **123**, 12392–12410 (2001).

67. H. Zhai, Data for classical quantum chemical solution of the FeMo-cofactor model to Chemical Accuracy and its Implications, Zenodo (2026); 10.5281/zenodo.22116121.

68. The PySCF Developers, The PySCF package, version 2.12.0, Zenodo (2026); 10.5281/zenodo.22116646.

69. H. Zhai, G. K.-L. Chan, block-hczhai/block2-preview (p0.5.4rc16). arXiv:2601.04621, Zenodo (2026); 10.5281/zenodo.22115816.

70. H. Zhai, G. K.-L. Chan, hczhai/hast-ucc. arXiv:2601.04621, Zenodo (2026); 10.5281/zenodo.22116003.

71. P. Zanello, Structure and electrochemistry of proteins harboring iron-sulfur clusters of different nuclearities. Part V. Nitrogenases. *Coord. Chem. Rev.* **398**, 113004 (2019).

72. H. Q. Huang, M. Kofford, F. B. Simpson, G. D. Watt, Purification, composition, charge, and molecular weight of the FeMo cofactor from azotobacter vinelandii nitrogenase. *J. Inorg. Biochem.* **52**, 59–75 (1993).

73. H. Jiang, O. K. Svensson, U. Ryde, Quantum mechanical calculations of redox potentials of the metal clusters in nitrogenase. *Molecules* **28**, 65 (2023).

74. W.-T. Jin, M. Yang, S.-S. Zhu, Z.-H. Zhou, Bond-valence analyses of the crystal structures of FeMo/V cofactors in FeMo/V proteins. *Acta Crystallogr. D Struct. Biol.* **76**, 428–437 (2020).

75. I. Dance, Computational investigations of the chemical mechanism of the enzyme nitrogenase. *Chembiochem* **21**, 1671–1709 (2020).

76. S. Raugei, L. C. Seefeldt, B. M. Hoffman, Critical computational analysis illuminates the reductive-elimination mechanism that activates nitrogenase for N2 reduction. *Proc. Natl. Acad. Sci. U.S.A.* **115**, E10521–E10530 (2018).

77. I. Dance, Survey of the geometric and electronic structures of the key hydrogenated forms of FeMo-co, the active site of the enzyme nitrogenase: Principles of the mechanistically significant coordination chemistry. *Inorganics* **7**, 8 (2019).

78. M. Torbjörnsson, U. Ryde, Comparison of the accuracy of DFT methods for reactions with relevance to nitrogenase. *Electron. Struct.* **3**, 034005 (2021).

79. B. Benediktsson, R. Björnsson, Analysis of the geometric and electronic structure of spin-coupled iron–sulfur dimers with broken-symmetry DFT: Implications for FeMoco. *J. Chem. Theory Comput.* **18**, 1437–1457 (2022).

80. K. Honjo, T. Kamimura, K. Tada, R. Kishi, Y. Kitagawa, Theoretical study of local environmental effect on electronic structure of M-cluster. *Eur. J. Inorg. Chem.* **28**, e202500016 (2025).

81. C. Van Stappen, B. Benediktsson, A. Rana, A. Chumakov, Y. Yoda, D. Bessas, L. Decamps, R. Bjornsson, S. De Beer, Structural correlations of nitrogenase active sites using nuclear resonance vibrational spectroscopy and QM/MM calculations. *Faraday Discuss.* **243**, 253–269 (2023).

82. G. K.-L. Chan, A. Keselman, N. Nakatani, Z. Li, S. R. White, Matrix product operators, matrix product states, and ab initio density matrix renormalization group algorithms. *J. Chem. Phys.* **145**, 014102 (2016).

83. J. Pipek, P. G. Mezey, A fast intrinsic localization procedure applicable for ab initio and semiempirical linear combination of atomic orbital wave functions. *J. Chem. Phys.* **90**, 4916–4926 (1989).

84. R. Olivares-Amaya, W. Hu, N. Nakatani, S. Sharma, J. Yang, G. K.-L. Chan, The ab-initio density matrix renormalization group in practice. *J. Chem. Phys.* **142**, 034102 (2015).

85. Q. Sun, T. C. Berkelbach, N. S. Blunt, G. H. Booth, S. Guo, Z. Li, J. Liu, J. D. McClain, E. R. Sayfutyarova, S. Sharma, S. Wouters, G. K.-L. Chan, PySCF: The Python-based simulations of chemistry framework. *WIREs Comput. Mol. Sci.* **8**, e1340 (2018).

86.. Legeza, G. Fáth, Accuracy of the density-matrix renormalization-group method. *Phys. Rev. B* **53**, 14349–14358 (1996).

87. K. Okunishi, Y. Hieida, Y. Akutsu, Universal asymptotic eigenvalue distribution of density matrices and corner transfer matrices in the thermodynamic limit. *Phys. Rev. E* **59**, R6227–R6230 (1999).

88. G. K.-L. Chan, P. W. Ayers, E. S. Croot III, On the distribution of eigenvalues of grand canonical density matrices. *J. Stat. Phys.* **109**, 289–299 (2002).

89. D. W. Berry, Y. Tong, T. Khattar, A. White, T. I. Kim, G. H. Low, S. Boixo, Z. Ding, L. Lin, S. Lee, G. K.-L. Chan, R. Babbush, N. C. Rubin, Rapid initial-state preparation for the quantum simulation of strongly correlated molecules. *PRX Quantum* **6**, 020327 (2025).

90. H. Schurkus, D.-T. Chen, H.-P. Cheng, G. K.-L. Chan, J. Stanton, Theoretical prediction of magnetic exchange coupling constants from broken-symmetry coupled cluster calculations. *J. Chem. Phys.* **152**, 234115 (2020).

91. H. Zhai, S. Lee, Z. H. Cui, L. Cao, U. Ryde, G. K.-L. Chan, Multireference protonation energetics of a dimeric model of nitrogenase iron–sulfur clusters. *J. Phys. Chem. A* **127**, 9974–9984 (2023).

92. S. Lee, H. Zhai, S. Sharma, C. J. Umrigar, G. K.-L. Chan, Externally corrected CCSD with renormalized perturbative triples (R-ecCCSD (T)) and the density matrix renormalization group and selected configuration interaction external sources. *J. Chem. Theory Comput.* **17**, 3414–3425 (2021).

93. A. D. Becke, Density-functional exchange-energy approximation with correct asymptotic behavior. *Phys. Rev. A* **38**, 3098–3100 (1988).

94. C. Lee, W. Yang, R. G. Parr, Development of the Colle-Salvetti correlation-energy formula into a functional of the electron density. *Phys. Rev. B* **37**, 785–789 (1988).

95. A. D. Becke, A new mixing of Hartree–Fock and local density-functional theories. *J. Chem. Phys.* **98**, 1372–1377 (1993).

96. F. E. Jorge, G. G. Camiletti, S. F. Machado, Contracted Gaussian basis sets for Douglas-Kroll-Hess calculations: Estimating scalar relativistic effects of some atomic and molecular properties. *J. Chem. Phys.* **130**, 064108 (2009).

97. E. Cancès, Y. Maday, B. Stamm, Domain decomposition for implicit solvation models. *J. Chem. Phys.* **139**, 054111 (2013).

98. F. Lipparini, Y. Maday, B. Stamm, E. Cancès, B. Mennucci, Fast domain decomposition algorithm for continuum solvation models: Energy and first derivatives. *J. Chem. Theory Comput.* **9**, 3637–3648 (2013).

99. F. Lipparini, G. Scalmani, L. Lagardère, B. Stamm, E. Cancès, Y. Maday, J. P. Piquemal, M. J. Frisch, B. Mennucci, Quantum, classical, and hybrid QM/MM calculations in solution: General implementation of the ddCOSMO linear scaling strategy. *J. Chem. Phys.* **141**, 184108 (2014).

100. A. Damle, L. Lin, L. Ying, Compressed representation of Kohn–Sham orbitals via selected columns of the density matrix. *J. Chem. Theory Comput.* **11**, 1463–1469 (2015).

101. C. Møller, M. S. Plesset, Note on an approximation treatment for many-electron systems. *Phys. Rev.* **46**, 618–622 (1934).

102. P. Pyykkö, M. Atsumi, Molecular single-bond covalent radii for elements 1–118. *Chem. A Eur. J.* **15**, 186–197 (2009).

103. R. D. Shannon, Revised effective ionic radii and systematic studies of interatomic distances in halides and chalcogenides. *Acta Crystallogr. A* **32**, 751–767 (1976).

104. P. W. Anderson, An approximate quantum theory of the antiferromagnetic ground state. *Phys. Rev.* **86**, 694–701 (1952).

105. F. Weigend, R. Ahlrichs, Balanced basis sets of split valence, triple zeta valence and quadruple zeta valence quality for H to Rn: Design and assessment of accuracy. *Phys. Chem. Chem. Phys.* **7**, 3297–3305 (2005).
